# Glycopyranosylidene-Spiro-Morpholinones: Evaluation of the Synthetic Possibilities Based on Glyculosonamide Derivatives and a New Method for the Construction of the Morpholine Ring

**DOI:** 10.3390/molecules27227785

**Published:** 2022-11-11

**Authors:** Nándor Kánya, Sándor Kun, László Somsák

**Affiliations:** Department of Organic Chemistry, University of Debrecen, PO Box 400, H-4002 Debrecen, Hungary

**Keywords:** morpholinone, spiro compound, glycopyranosylidene-spiro-morpholinone, ozonolysis

## Abstract

Glycosylidene-spiro-morpholin(on)es are scarcely described skeletons in the literature. In this work, we have systematically explored the synthetic routes towards such morpholinones based on the reactions of *O*-peracylated hept-2-ulopyranosonamide derivatives of D-*gluco* and D-*galacto* configuration. Koenigs–Knorr type glycosylation of 2-chloroethanol, allylic and propargylic alcohols by (glyculosylbromide)onamides furnished the expected glycosides. The 2-chloroethyl glycosides were ring closed to the corresponding spiro-morpholinones by treatment with K_2_CO_3_. The (allyl glyculosid)onamides gave diastereomeric mixtures of spiro-5-hydroxymorpholinones by ozonolysis and 5-iodomethylmorpholinones under iodonium ion mediated conditions. The ozonolytic method has not yet been known for the construction of morpholine rings, therefore, it was also extended to *O*-allyl mandelamide. The 5-hydroxymorpholinones were subjected to oxidation and acid catalyzed elimination reactions to give the corresponding morpholine-3,5-dions and 5,6-didehydro-morpholin-3-ones, respectively. Base induced elimination of the 5-iodomethylmorpholinones gave 5-methyl-2*H*-1,4-oxazin-3(4*H*)-ones. *O*-Acyl protecting groups of all of the above compounds were removed under Zemplén conditions. Some of the D-*gluco* configured unprotected compounds were tested as inhibitors of glycogen phosphorylase, but showed no significant effect.

## 1. Introduction

Spiro compounds are molecules which contain a bi- or tricyclic system with a shared single atom between two rings. These represent a fast growing class of molecules [1] of both natural and synthetic origin. The importance of such compounds is incontrovertible, since they, among others, often exhibit interesting biological properties [2,3,4,5]. Carbohydrate-derived spirocycles as well as spiro-iminosugars [6] are also an emerging class of compounds of high biological significance with well-known representatives such as the antifungal papulacandins, the antibiotic orthosomycins, the herbicidal hydantocidin, each of natural origin, and the synthetic tofogliflozin, the active ingredient of approved antidiabetic medications [7].

Morpholine [8,9] and its derivatives, especially morpholinones [10], are frequent constituents of approved and investigational drugs, and are considered as privileged heterocycles in drug design [8,11,12]. 

**Chart 1:**
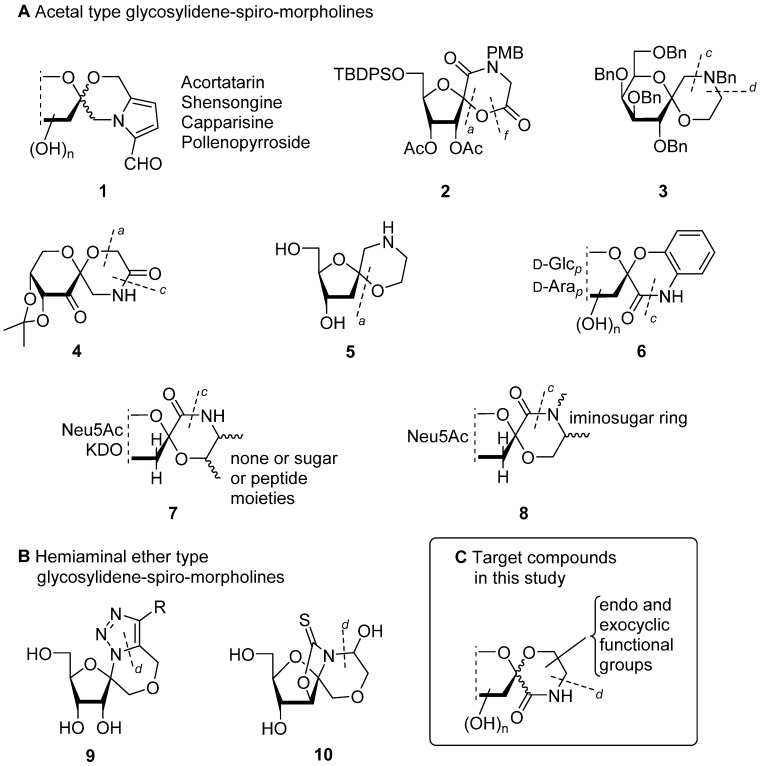
Glycosylidene-spiro-morpholines known from the literature (**A**,**B**), and target compounds of the present work (**C**). Dashed lines indicate ring closing bond formations used in the construction of the morpholin(on)e moieties (for detailed explanation, please, see text).

Glycosylidene-spiro-morpholines, depending on the position of the spiro carbon in the morpholine ring, can be classified as acetal and hemiaminal ether type compounds (Chart 1, **A** and **B**, respectively). Glycosylidene-spiro-morpholines **1** with an annulated formyl-pyrrole moiety were isolated from natural sources in the past decade and their structures, properties and syntheses were reviewed [13,14]. Several synthetic intermediates, such as **2** [15], **3** [16], **4** [17,18] and **5** [19], also contained this structural motif. 

Ring annulated derivatives, such as benzoxazinones [20,21] **6** and sialoconjugated spiro-morpholinones with no additional substituents [22] or with various sugar [23,24,25,26,27] or peptide [28] moieties (**7**) as well as iminosugars [29,30] (**8**) attached to the morpholine ring, were synthesized for diverse biological purposes. 

The synthetic methods applied to obtain the above compounds followed known bond formations for the construction of the morpholin(on)e ring. Simultaneous formations of two bonds were used in the syntheses of **2** (Baeyer–Villiger oxidation of a spiro pyrrolidin-2,4-dione to obtain bonds *a*,*f* [15]), **3** (ozonolysis of a 1-*C*-vinyl allyl glycoside followed by reductive amination to make bonds *c*,*d* [16]) and **4** (reaction of a sugar derived β-aminoalcohol with bromoacetylbromide toward bonds *a*,*c* [17,18]). Single bond forming ring closures of suitable precursors were applied in the syntheses of **5** (acid catalyzed OH addition to the double bond in a 1-(2-hydroxyethylaminomethyl) substituted *endo*-glycal to obtain bond *a* [19]), and **6**–**8** (nucleophilic substitution of the ester in the sialic acid by an amine obtained in situ from an azide, protected amine/iminosugar or nitro group to form bond *c* [20,21,22,23,24,25,26,27,28,29,30]). In the preparation of the much less known hemiaminal ether type spiro-morpholines (Chart 1B), intramolecular azide-alkyne cycloaddition was used to give bond *d* of **9** [31,32]. Similarly, bond *d* was formed from an aldehyde/ester and a cyclic thioamide to close the morpholine ring of **10** [33]. The formation of bond *d* is also known in the syntheses of morpholin-3-ones, some examples of ring closing reactions of α-(2-chloroethoxy)-amides under basic conditions are mentioned in a review [10].

In this study, we aimed at the investigation of synthetic possibilities of glycopyranosylidene-spiro-morpholinones (Chart 1C) based on readily available glyculopyranosonamide derivatives and explored the potential of reactions to form bond *d* of morpholin-3-ones. 

## 2. Results and Discussion

**Scheme 1:**
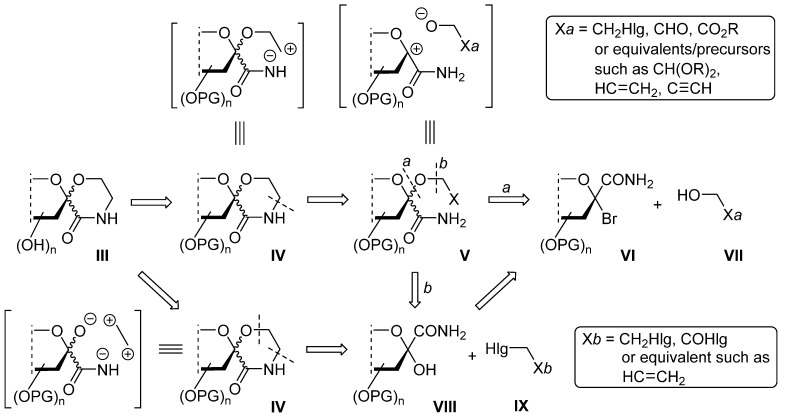
Retrosynthetic analysis of the target glycopyranosylidene-spiro-morpholinones.

A retrosynthetic analysis of the target compounds **III** (Scheme 1) allowed us to easily identify (glyculopyranosylbromide)onamides **VI** as starting materials which, on glycosylation of 2-EWG substituted ethanol derivatives **VII** (or equivalents), were expected to furnish suitable substrates **V** for morpholinone ring closure to obtain the *O*-protected target compounds **IV**. Alternatively, reactions of glyculopyranosonamides **VIII**, also to be obtained from **VI**, with dielectrophiles **IX** might furnish cyclizable intermediate **V**, apparently with the opposite anomeric configuration.

**Scheme 2:**
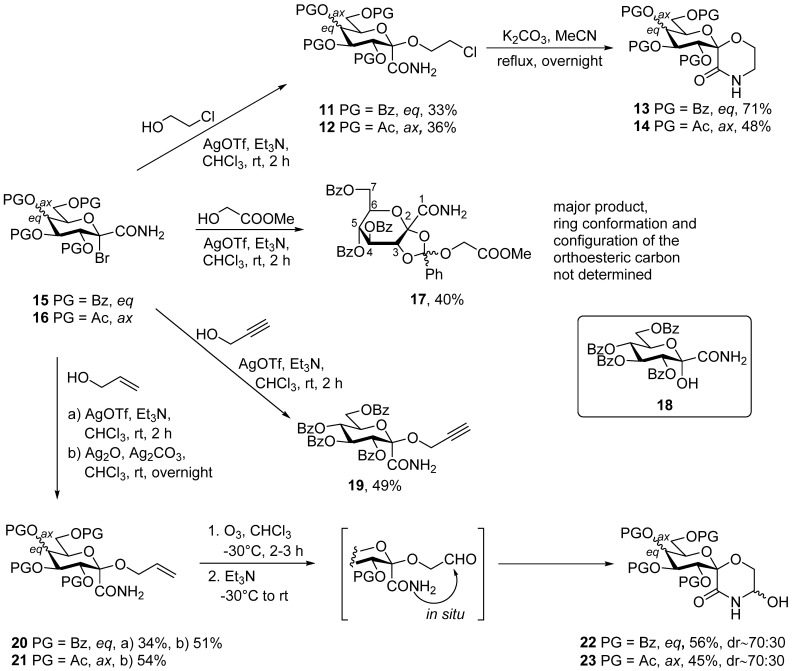
Synthetic studies towards *O*-peracylated glycopyranosylidene-spiro-morpholinones.

The *O*-peracylated (glyculopyranosylbromide)onamides **15** [34] and **16** [35] were obtained from the corresponding free sugars in five synthetic steps as described in the literature [36,37]. Glycosylation of simple alcohols with **15** and **16** was studied earlier [38], thus, under similar conditions using AgOTf as the promoter, the required 2-chloroethyl glycosides **11** and **12** were obtained in acceptable yields (Scheme 2). Ring closure of **11** and **12** was effected in the presence of K_2_CO_3_ in boiling acetonitrile to give spiro-morpholinones **13** and **14** in 71 and 48% yields, respectively. To form additional functional groups on the morpholinone ring, first the reaction of glycolic acid methyl ester and **15** was carried out under the above glycosylation conditions. One major compound was formed, nevertheless, this proved to be the orthoester type derivative **17** based on the ^1^H and ^13^C NMR spectra. Small coupling constants (1–3 Hz) in the pyranose H-3, H-4, H-5 multiplets clearly indicated an unusual conformation of the ring. ^13^C NMR of **17** showed the presence of an *O*-glycoside (C-2 at 102.2 ppm). A missing set of benzoyl peaks from the 165–170 ppm (carbonyl) and the 133–134 (*para* carbon relative to the carbonyl group) regions and the appearance of quaternary signals at 134.1 (phenyl carbon connected to the orthoesteric carbon) and 122.3 (orthoesteric carbon) suggested the depicted structure. We also considered glycolaldehyde or its precursor acetal as the next coupling partners, however, due to easier handling and a significantly lower price, allyl and propargyl alcohols, that can be regarded as latent carbonyl derivatives, were studied. Propargyl alcohol was glycosylated with **15** under the above conditions to give the corresponding glycoside **19** in acceptable yield. Glycosylation of allyl alcohol by **15** with the AgOTf promoter gave **20** in moderate yield (34%) and a significant amount (~10%) of the hydrolysis product **18** [34], therefore, we turned to Ag_2_O/Ag_2_CO_3_ as the promoter to give allyl glycosides **20** and **21** in better yields (>50%) and without competing hydrolysis. Silver(I) promoted *O*-glycosylations of **15** and **16** proceed with the inversion of the anomeric carbon, as proved by X-ray [38], NMR and TDDFT-ECD [20] methods. This was also observed in the present cases wherein the hydrogens (H-3: 5.9–6.6 ppm, H-5: 4.9–5.1 ppm for compounds **11**, **12**, **19**, **20**, **21**) on the α-side of the pyranose ring in ^4^*C*_1_ conformation characteristically shifted downfield due to the deshielding effect of the carbonyl group.

Several attempts to cyclize propargyl glycoside **19** to a morpholine ring (treatment with NaH; in the presence of Pd(OAc)_2_ with or without added PPh_3_ [39,40]) proved unsuccessful.

Allyl glycosides **20** and **21** were subjected to ozonolysis conditions and hydroxymorpholinones **22** and **23**, respectively, could be isolated from the reaction mixtures in medium yields. Compounds **22** and **23** were formed as diastereomeric mixtures whose ratio (Scheme 2) could be easily deduced from the ^1^H NMR spectra of the product mixtures. Since the final aim of this study was to arrive at stereochemically uniform products (vide infra), no attempt was made either to separate these isomers or to determine the configuration of C-5 of the heteroring. The ring closure can be understood by the intramolecular addition of the carboxamide nitrogen to the in situ formed aldehyde. Literature analogies for the synthesis of piperidines/δ-lactams via amide addition to aldehyde prepared in situ from allylic/vinylic moieties by OsO_4_/NaIO_4_ [41,42] and O_3_ [43] are known, but, to the best of our knowledge, morpholine rings have not yet been obtained in this manner.

**Scheme 3:**
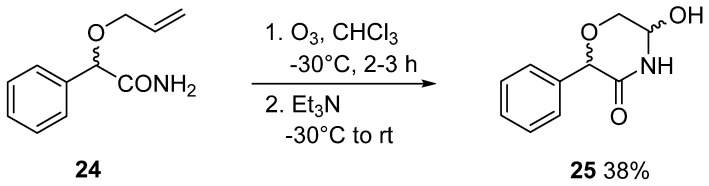
Extension of the ozonolytic morpholine ring closure to mandelamide derivative **24**.

This morpholine ring forming reaction was also applied to racemic *O*-allylated mandelamide **24** (for its synthesis, see the electronic supporting information) to give hydroxymorpholinones **25** in moderate yield (Scheme 3). Due to the presence of two stereogenic centers in **25**, the formation of four diastereomers can be expected. However, these form two pairs of enantiomers, therefore, in the ^1^H NMR spectrum only two series of signals can be observed in a 5:1 ratio.

**Scheme 4:**
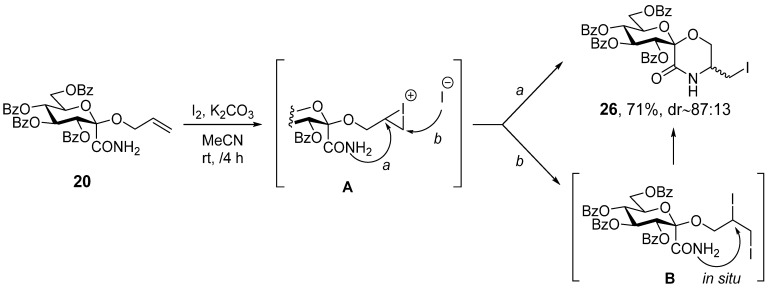
Iodocyclization of allyl glycoside **20**.

Olefinic carbons in the allyl moiety can also be turned into electrophilic centers by halogens or halonium ion equivalents such as *N*-halosuccinimides, and this has been utilized in morpholine/morpholinone ring closing transformations, too [44,45,46]. Thus, allyl glycoside **20** was treated by iodine in the presence of K_2_CO_3_ to give a good yield of iodomethyl spiro-morpholinones **26** as a mixture of diastereomers (Scheme 4). Separation of the stereoisomers or determination of the configuration of the new stereogenic center was not attempted. Formation of the morpholinone ring of **26** can be understood by a nucleophilic attack of the carboxamide nitrogen on the cyclic iodonium ion in **A** (route *a* in Scheme 3) or, alternatively, on the diiodo intermediate **B** (route *b*). According to Baldwin’s rules, attack of the nitrogen on the terminal carbon could also result in a 6-iodo-1,4-oxazepan-3-one derivative, but the appearance of a negative phase CH_2_ peak in the ^13^C J-MOD spectrum at 2.9 ppm can only correspond to the iodomethyl group of structure **26**. This observation also matches literature experiences [45].

We have also considered the transformation of gluculopyranosonamide **18** [34] to obtain the spiro-epimer of the glycopyranosylidene-spiro-morpholines obtained so far. Reactions with 1,2-dielectrophiles (BrCH_2_CH_2_Br, BrCH_2_COBr) and attempts for the allylation of the hindered OH group of **18** under several conditions (listed in Tables S1 and S2 in the supporting information) were tried, but remained unsuccessful.

**Scheme 5:**
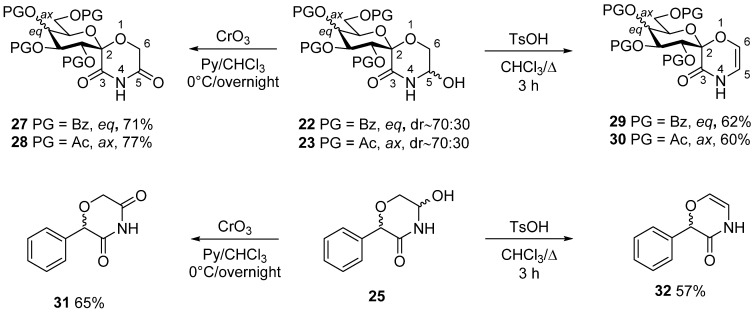
Oxidation and elimination reactions of hydroxymorpholinone derivatives.

Diastereomeric mixtures of **22**, **23** and **25** were subjected to further transformations to remove the stereogenic centers at C-5 of the morpholine rings (Scheme 5). Thus, oxidation of the hydroxymorpholinones **22**, **23** and **25** by CrO_3_ furnished the respective spiro-morpholine-diones **27**, **28** and **31** while acid catalyzed water elimination gave the unsaturated derivatives **29**, **30** and **32**, respectively. The transformations could be clearly followed based on the changes of the ^13^C NMR spectra. Disappearance of the hemiaminal C-5 (67–74 ppm) and appearance of new carbonyl (C-5: 165–171 ppm in dions **27**, **28**, **31**) and olefinic (C-5: 106–107 ppm, C-6: 126–129 ppm in unsaturated morpholinones **29**, **30**, **32**) peaks verified the formation of said compounds. Catalytic hydrogenation of **29** (H_2_ atmosphere, Pd/C in EtOAc at rt for 3 h) resulted in an 80% yield of **13** obtained earlier (Scheme 2) in a more straightforward way.

**Scheme 6:**
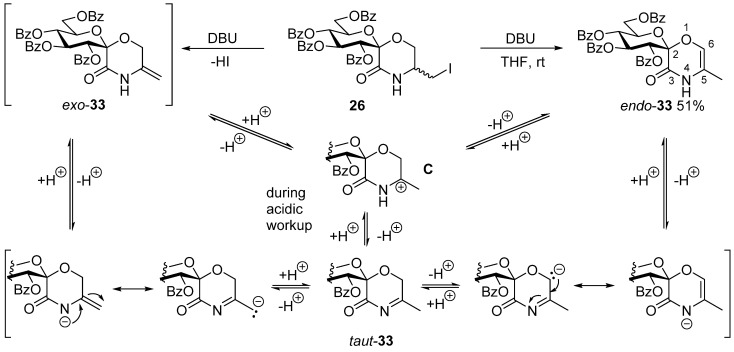
Hydrogen iodide elimination from **26** and a mechanistic rationale for the formation of the isolated product *endo*-**33**.

Iodomethyl morpholinone **26** underwent a hydrogen iodide elimination on the action of DBU, however, instead of the expected exomethylene derivative *exo*-**33**, compound *endo*-**33** with an endocyclic double bond was formed as evidenced by the ^1^H NMR singlets at 5.98 ppm (H-6) and 1.79 ppm (CH_3_) (Scheme 6). Though no experimental mechanistic studies were performed, two plausible pathways, based on protonation–deprotonation steps in an equilibrium, can be raised for the formation of *endo*-**33**. The primary product of the base induced elimination is probably *exo*-**33** which in a deprotonation–protonation sequence may lead to *taut*-**33**. A subsequent deprotonation–protonation may lead to *endo*-**33** which, containing the more substituted double bond as compared to *exo*-**33**, is the thermodynamically more stable product of the equilibration. Alternatively, protonation of *exo*-**33** during the acidic workup may give carbocation **C** as an intermediate of the equilibrium towards *endo*-**33** of lower energy.

**Scheme 7:**
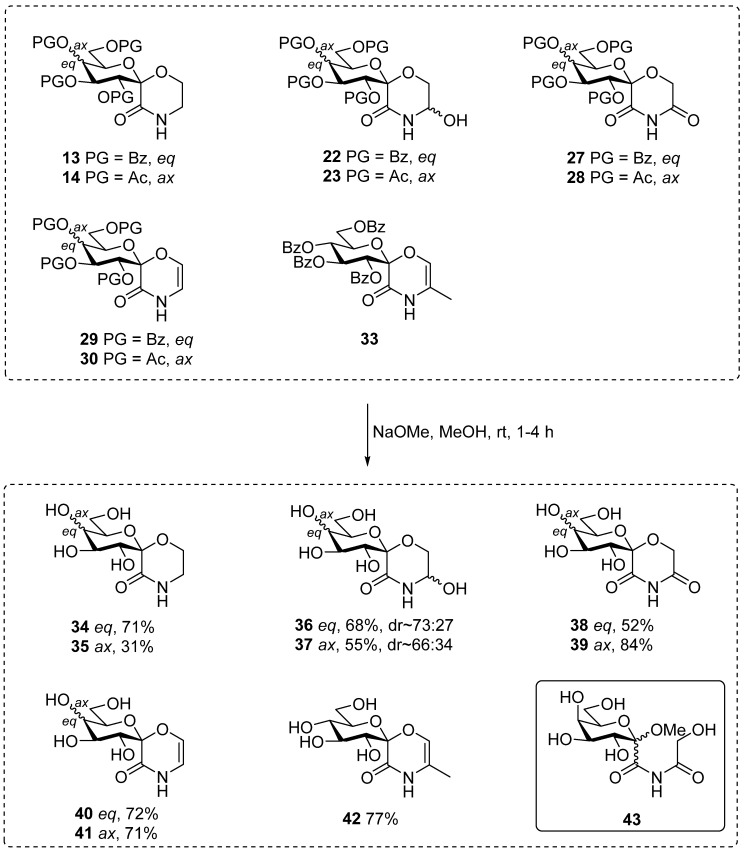
Deprotection of the synthesized spiro-morpholinones.

Finally, the protecting groups of the sugar derived spiro-morpholine derivatives **13**, **14**, **22**, **23**, **27**–**30** and (*endo*-)**33** were removed under standard Zemplén deacylation conditions to give the respective compounds **34**–**42** in good to acceptable yields (Scheme 7). In some cases, during silica gel column chromatographic purification of the products, the opening of the morpholine ring was observed to give the corresponding methyl glycoside type side product such as **43** as the result of an acid catalyzed transglycosylation. Except for the synthesis of compound **39** (15% of inseparable methyl glycoside **43** formation), this process could be completely suppressed with a small amount of triethylamine added to the chromatographic eluent.

Compounds **36**, **38** and **40** were assayed for glycogen phosphorylase inhibition and showed negligible inhibitory effect (20, 27 and 23% inhibition, respectively, in 625 µM).

## 3. Experimental

Melting points are uncorrected, and were measured with a Kofler hot-stage. Optical rotations were determined at room temperature using a Perkin-Elmer 241 polarimeter. ^1^H and ^13^C NMR spectra were recorded with a Bruker DRX 360 (^1^H: 360 MHz, ^13^C: 90 MHz) or a Bruker DRX 400 (^1^H: 400 MHz, ^13^C: 100 MHz) spectrometer. The 1D ROESY and HSQMBC measurements were recorded on a Bruker Avance II 500 (^1^H: 500 MHz, ^13^C: 125 MHz) spectrometer. Chemical shifts were referenced to Me_4_Si (^1^H) or to the residual solvent signals (^13^C). Mass spectra were recorded with a Bruker micrOTOF-Q and Thermo Accela LTQ XL or a Bruker maXis II UHR ESI-TOF (HRMS) spectrometer.

Thin layer chromatography was performed using DC-Alurolle Kieselgel F_254_ (Merck) plates and the spots were visualized under UV light (λ = 254 nm) and by gentle heating after staining the plate (staining solutions: 1% of anisaldehyde and 5% of cc. sulfuric acid in ethanol or 6% of vanilline and 1% of cc. sulfuric acid in ethanol). For the detection of bromine containing compounds, the plates were sprayed with a fluorescein solution (0.01% in ethanol) followed by a solution of hydrogen peroxide (1:1 mixture of 30% aqueous H_2_O_2_ and glacial acetic acid). Bromides appeared as a pink spot after gentle heating of the plate. For column chromatography, Kieselgel 60 (Merck, particle size: 0.063–0.200 mm) type silica gel was used. Anhydrous chloroform was prepared from commercial chloroform stabilized with 1% of EtOH. It was kept on anhydrous CaCl_2_ overnight, then filtered and distilled from P_2_O_5_ and the collected distillate was stored over molecular sieves (4Å). Other solvents were dried by conventional methods.

### 3.1. General Method I. for the Synthesis of C-(2,3,4,6-Tetra-O-Acyl-1-Alkoxy-D-Glycopyranosyl)Formamides *(**11**, **12**, **17**, **19**, **20**, **21**)*

In a flame dried round bottom flask, a bromoamide **15** or **16**, (0.5 mmol) was dissolved in anhydrous chloroform (5 mL). Triethylamine (2 equiv.), the corresponding alcohol (20 equiv.) and silver triflate (2 equiv.) were added and the flask was covered in tin foil. After 2 h of stirring at room temperature, no starting material could be detected by TLC. Evaporation of the solvent gave a syrup, which was purified by column chromatography.

### 3.2. General Method II. for the Synthesis of C-(2,3,4,6-Tetra-O-Acyl-1-Alkoxy-D-Glycopyranosyl)Formamides *(**17**, **20**)*

In a flame dried round bottom flask, a bromoamide **15** or **16**, (0.5 mmol) was dissolved in anhydrous chloroform (5 mL). Silver oxide (2 equiv.), silver carbonate (2 equiv.) and the corresponding alcohol (20 equiv.) were added and the mixture was stirred overnight. After TLC showed complete conversion, the mixture was filtered through a celite pad. Evaporation of the solvent gave a syrup, which was purified by column chromatography.

### 3.3. General Method III. for the Ring Closure Reactions of C-(2,3,4,6-Tetra-O-Acyl-1-(2-Chloroethoxy)-α-D-Glycopyranosyl)Formamides *(**11**, **12**)* into Spiro-Morpholin-3-Ones *(**13**, **14**)*

2-Chloroethyl glycoside (**11** or **12**) was dissolved in dry acetonitrile (~1 mL/50 μmol), and flame dried potassium carbonate (2 equiv.) was added. The mixture was stirred at reflux temperature until TLC showed full conversion. Insoluble materials were removed by filtration. The solvent was removed by evaporation and the residue was purified by column chromatography.

### 3.4. General Method IV. for the Ozonolysis of C-(2,3,4,6-Tetra-O-Acyl-1-Allyloxy-D-Glycopyranosyl)Formamides *(**20**, **21**)* and 2-Allyloxy-2-Phenylacetamide *(**24**)* to Yield 5-Hydroxymorpholin-3-Ones *(**22**, **23**, **25**)*

In a flame dried round bottom flask, an α-allyloxyamide (**20**, **21**, **24**, 0.8–1.4 mmol) was dissolved in dry chloroform (~1 mL/80 μmol), and the solution was cooled to −30 °C. Ozone was bubbled through the reaction mixture for 2–3 h. The progress of the reaction was monitored by TLC: a sample (1–2 drops) was taken from the mixture, diluted with chloroform and 1–2 drops of triethylamine were added then applied to a TLC plate. After total consumption of the starting material, the ozone flow was stopped, triethylamine (2 equiv.) was added and the mixture was allowed to reach room temperature. After evaporation of the solvent, the product was obtained by column chromatography (hexane/acetone 2:1).

### 3.5. General Method V. for the Synthesis of Morpholinediones *(**27**, **28**, **31**)*

In a flame dried round bottom flask, a 5-hydroxymorpholinone derivative (**22**, **23**, **25**, 0.5–0.8 mmol) was dissolved in dry chloroform (1 mL/0.1 mmol), the solution was cooled to 0 °C then pyridine (5 equiv.) and chromium(VI) oxide (5 equiv.) were added to the stirred solution. The solution was slowly allowed to warm up and stirred at room temperature overnight. The next day, TLC showed full conversion, and a brown precipitate appeared. The solution was diluted with chloroform (20 mL), filtered and concentrated. A few milliliters of toluene was also added and evaporated to remove most of the pyridine. The crude product was purified by column chromatography (hexane/acetone 4:1 → 3:1).

### 3.6. General Method VI. for the Synthesis of Unsaturated Morpholinones *(**29**, **30**, **32**)*

A 5-hydroxymorpholinone derivative (**22**, **23**, **25**, 0.2–0.5 mmol) was dissolved in dry chloroform (~1 mL/35 μmol), and *p*-toluenesulfonic acid (0.2 equiv.) was added. The solution was stirred and refluxed for 2–3 h, after which time TLC showed complete conversion. The solvent was evaporated, and the crude product was purified by column chromatography (hexane/acetone 3:1).

### 3.7. General Method VII. for the Removal of O-Acyl Groups to Yield Deprotected Compounds *(**34**–**42**)*

An *O*-peracylated spiro-morpholine derivative (**13**, **14**, **22**, **23**, **27**–**30**, **33**, 0.1–0.7 mmol) was dissolved in dry methanol (1 mL/~30–40 μmol), and pH was adjusted to ~10–11 with 1M NaOMe/MeOH solution (approx. 10–15 drops). The reaction mixture was stirred at room temperature for 1–4 h until TLC showed complete conversion. The solution was neutralized using acidic ion exchange resin (Amberlyst 15^®^) until pH ~6–7 was reached, the resin was filtered and the filtrate was concentrated. The products were purified by column chromatography (chloroform/methanol 8:1–3:1, triethylamine (0.5% *V*/*V*) was added to the eluents).

### 3.8. C-(2,3,4,6-Tetra-O-Benzoyl-1-(2-Chloroethoxy)-α-D-Glucopyranosyl)Formamide *(**11**)*

Prepared according to General method I. from **15** (300 mg, 0.43 mmol) and 2-chloroethanol. Column chromatography (hexane/acetone 3:1 → 2:1) gave 100 mg (33%), colorless oil. R_f_ = 0.34 (hexane/acetone 3:1). [α]_D_ = +54 (c = 0.50, CHCl_3_); ^1^H NMR (400 MHz, CDCl_3_) δ (ppm): 8.07 (2H, Ar), 7.95 (4H, Ar), 7.83 (2H, Ar), 7.59–7.25 (12H, Ar), 6.80 (1H, d, *J* = 3.2 Hz, NH), 6.63 (1H, t, *J* = 9.3 Hz, H-3), 5.92 (1H, brs, NH), 5.83 (1H, t, *J* = 9.8 Hz, H-4), 5.77 (1H, d, *J* = 9.2 Hz, H-2), 5.09 (1H, dt, *J* = 10.1, 3.2 Hz, H-5), 4.73 (1H, dd, *J* = 12.4, 2.8 Hz, H-6a), 4.42 (1H, dd, *J* = 12.4, 3.7 Hz, H-6b), 4.13–4.03 (2H, m, OC*H_2_*), 3.68–3.56 (2H, m C*H_2_*Cl). ^13^C NMR (100 MHz, CDCl_3_) δ (ppm): 169.3 (*C*ONH_2_), 166.2, 165.4 (2), 165.2 (4 × *C*=O), 133.7–128.4 (Ar), 97.7 (C-1), 72.7, 71.9, 69.7, 68.9 (C-2–C-5), 63.1, 62.5 (C-6, O*C*H_2_), 43.1 (*C*H_2_Cl). HRMS (positive mode, m/z): 724.1557 (calculated for C_37_H_32_ClNO_11_Na: 724.1556).

### 3.9. C-(2,3,4,6-Tetra-O-Acetyl-1-(2-Chloroethoxy)-α-D-Galactopyranosyl)Formamide *(**12**)*

Prepared according to General method I. from **16** (200 mg, 0.44 mmol) and 2-chloroethanol. Column chromatography (hexane/acetone 3:1 → 2:1) gave 100 mg (36%), colorless oil. R_f_ = 0.34 (hexane/acetone 3:1). [α]_D_ = +70 (c = 0.51, CHCl_3_); ^1^H NMR (400 MHz, CDCl_3_) δ (ppm): 6.74 (1H, d, *J* = 3.1 Hz, NH), 5.90 (1H, dd, *J* = 10.5, 3.3 Hz, H-3), 5.86 (brs, 1H, NH), 5.53 (1H, dd, *J* = 3.3, 1.2 Hz, H-4), 5.47 (1H, d, *J* = 10.5 Hz, H-2), 4.87 (1H, t, *J* = 6.9 Hz, H-5), 4.10 (2H, d, *J* = 6.6 Hz, OC*H_2_*), 4.06 (1H, dd, *J* = 11.2, 5.2 Hz, H-6a), 3.98 (1H, dd, *J* = 10.7, 5.4 Hz, H-6b), 3.71–3.62 (2H, m, C*H*_2_Cl), 2.17, 2.08, 2.04, 1.98 (4 × 3H, s, 4 × OCOC*H_3_*). ^13^C NMR (90 MHz, CDCl_3_) δ (ppm): 170.5, 170.1, 170.0, 169.8, 169.3 (5 × *C*=O), 97.7 (C-1), 71.6, 69.9, 67.9, 66.9 (C-2–C-5), 62.7, 61.5 (C-6, O*C*H_2_), 43.3 (*C*H_2_Cl), 20.9, 20.8, 20.7, 20.7 (4 × OCO*C*H_3_).

### 3.10. (1′S)-1′,5′-Anhydro-2′,3′,4′,6′-Tetra-O-Benzoyl-D-Glucitol-Spiro-[1′,2]-Morpholin-3-One *(**13**)*

Prepared according to General method III. from **11** (100 mg, 0.14 mmol). Reaction time: 30 h. Column chromatography (hexane/acetone 3:1) gave 60 mg (71%) colorless, amorphous solid. R_f_ = 0.42 (hexane/acetone 3:1). [α]_D_ = +17 (c = 0.25, CHCl_3_); ^1^H NMR (400 MHz, CDCl_3_) δ (ppm): 8.06–7.81 (8H, m Ar), 7.57–7.25 (12H, Ar), 6.70 (1H, t, *J* = 9.8 Hz, H-3′), 6.50 (1H, d, *J* = 3.9 Hz, NH), 5.81 (1H, t, *J* = 9.8 Hz, H-4′), 5.68 (1H, d, *J* = 9.9 Hz, H-2′), 5.0 (1H, dt, *J* = 10.0, 3.5 Hz, H-5′), 4.65 (1H, dd, *J* = 12.3, 2.9 Hz, H-6′a), 4.44 (1H, dd, *J* = 12.3, 4.1 Hz, H-6′b), 4.26 (1H, td, *J* = 11.9, 3.0 Hz, H-6a), 3.88 (1H, dd, *J* = 11.9, 3.8 Hz, H-6b), 3.49 (1H, td, *J* = 12.1, 4.2 Hz, H-5a), 3.23 (1H, dt, *J* = 12.2, 3.2 Hz, H-5b). ^13^C NMR (100 MHz, CDCl_3_) δ (ppm): 166.4, 165.7, 165.5, 165.4, 165.2 (5 × *C*=O), 133.5–128.3 (Ar), 97.5 (C-1′), 73.8, 72.4, 72.4, 69.5 (C-2′–C-5′), 63.1 (C-6′), 59.3 (C-6), 41.5 (C-5). HSQMBC NMR: ^3^*J*_H-2′, CO_ = 5.0 Hz; HRMS (positive mode, m/z): 688.1790 (calculated value for C_37_H_31_NO_11_Na: 688.1789).

### 3.11. (1′S)-2′,3′,4′,6′-Tetra-O-Acetyl-1′,5′-Anhydro-D-Galactitol-Spiro-[1′,2]-Morpholin-3-One *(**14**)*

Prepared according to General method III. from **12** (100 mg, 0.22 mmol). Reaction time: 24 h. Column chromatography (hexane/acetone 3:1) gave 44 mg (48%), colorless, amorphous solid. R_f_ = 0.35 (hexane/acetone 3:1). [α]_D_ = +29 (c = 0.40, CHCl_3_); ^1^H NMR (400 MHz, CDCl_3_) δ (ppm): 7.18 (1H, brs, NH), 5.93 (1H, dd, *J* = 10.6, 3.5 Hz, H-3′), 5.52 (1H, dd, *J* = 3.5, 1.1 Hz, H-4′), 5.38 (1H, d, *J* = 10.6 Hz, H-2′), 4.75 (1H, ddd, *J* = 7.6, 6.2, 1.4 Hz, H-5′), 4.23 (1H, td, *J* = 12.0, 3.1 Hz, H-6a), 4.18–4.08 (2H, m, H-6b, H-6′a), 3.91 (1H, dd, *J* = 11.9, 4.0 Hz, H-6′b), 3.58 (1H, td, *J* = 12.2, 4.2 Hz, H-5a), 3.27 (1H, dt, *J =* 12.4, 3.6 Hz, H-5b), 2.17, 2.09, 2.04, 1.96 (4 × 3H, s, 4 × OCOC*H_3_*). ^13^C NMR (100 MHz, CDCl_3_) δ (ppm): 170.5, 170.4, 170.1, 169.6 (4 × *C*=O), 165.6 (C-3), 97.4 (C-1′), 71.5, 70.4, 70.1, 67.4 (C-2′–C-5′), 61.6, 59.2 (C-6, C-6′), 41.3 (C-5), 20.9, 20.8 (2), 20.8 (4 × OCO*C*H_3_).

### 3.12. 4,5,7-Tri-O-Benzoyl-2,3-O-[α-(2-Methoxy-2-Oxoethoxy)Benzylidene]-α-D-Gluco-Hept-2-Ulopyranosonamide *(**17**)*

Prepared according to General method I. from **15** (300 mg, 0.43 mmol) and methyl glycolate. Column chromatography (hexane/ethyl acetate 7:3 → 6:4) gave 122 mg (40%), colorless oil. Preparation according to General method II. from **15** (300 mg, 0.43 mmol) and methyl glycolate gave 102 mg (34%). R_f_ = 0.28 (hexane/ethyl acetate 1:1). [α]_D_ = +45 (c = 1.00, CHCl_3_); ^1^H NMR (400 MHz, CDCl_3_) δ (ppm): 8.04–7.20 (20H, m, Ar), 7.10 (1H, s, N*H*), 5.78 (1H, dd, *J* = 3.1, 1.0 Hz, H-2), 5.67 (1H, s, N*H*), 5.55 (1H, dt, *J* = 8.6, 1.2 Hz, H-5), 5.24 (1H, dd, *J* = 3.1, 1.3 Hz, H-4), 4.60 (1H, dd, *J* = 12.2, 2.9 Hz, H-7a), 4.44 (1H, dd, *J* = 12.2, 4.9 Hz, H-7b), 4.13 (1H, m, H-5), 4.11 (1H, d, *J* = 16.0 Hz, C*H*_2_COOCH_3_), 4.04 (1H, d, *J* = 16.0 Hz, C*H*_2_COOCH_3_), 3.71 (3H, s, CH_2_COOC*H_3_*). ^13^C NMR (90 MHz, CDCl_3_) δ (ppm): 169.6, 168.4, 166.2, 165.2, 164.6 (5 × *C*=O), 134.1–122.3 (Ar), 102.1 (C-2), 74.3, 69.6, 68.7, 67.9 (C-3–C-6), 63.9, 62.1 (C-7, *C*H_2_COOCH_3_), 52.1 (COO*C*H_3_). HRMS (positive mode, m/z): 734.1840 (calculated value for C_38_H_33_NO_13_Na: 734.1844).

### 3.13. C-(2,3,4,6-Tetra-O-Benzoyl-1-Propargyloxy-α-D-Glucopyranosyl)Formamide *(**19**)*

Prepared according to General method I. from **15** (150 mg, 0.21 mmol) and propargyl alcohol. Column chromatography (hexane/acetone 9:1 → 4:1) gave 71 mg (49%), colorless oil. R_f_ = 0.42 (hexane/acetone 3:1). [α]_D_ = +38 (c = 0.30, CHCl_3_); ^1^H NMR (360 MHz, CDCl_3_) δ (ppm): 8.07–7.84 (8H, m, Ar), 7.59–7.28 (12H, m, Ar), 6.76 (1H, d, *J* = 3.1 Hz, NH), 6.57 (1H, t, *J* = 9.1 Hz, H-3), 5.84–5.78 (3H, m, H-2, H-4, NH), 5.11 (1H, dt, *J* = 9.6, 3.4 Hz, H-5), 4.71 (1H, dd, *J* = 12.3, 2.9 Hz, H-6a), 4.54 (2H, dd, *J* = 2.6, 1.3 Hz, -OC*H_2_*-C≡CH), 4.46 (1H, dd, *J* = 12.4, 3.9 Hz, H-6b), 2.42 (1H, t, *J* = 2.4 Hz, -OCH_2_-C≡C*H*). ^13^C NMR (90 MHz, CDCl_3_) δ (ppm): 168.9, 166.2, 165.5, 165.4, 165.0 (5 × *C*=O), 133.7–128.4 (Ar), 97.7 (C-1), 78.6 (-OCH_2_-C≡*C*H), 75.1 (-OCH_2_-*C*≡CH), 72.8, 71.8, 69.6, 68.8 (C-2–C-5), 62.7 (C-6), 51.6 (-O*C*H_2_-C≡CH).

### 3.14. C-(1-Allyloxy-2,3,4,6-Tetra-O-Benzoyl-α-D-Glucopyranosyl)Formamide *(**20**)*

Prepared according to General method I. from **15** (100 mg, 0.14 mmol) and allyl alcohol to give 45 mg (43%) white powder. Preparation according to General method II. gave 540 mg (51%). R_f_ = 0.16 (hexane/acetone 3:1). [α]_D_ = +49 (c = 0.48, CHCl_3_); ^1^H NMR (360 MHz, CDCl_3_) δ (ppm): 8.08–7.85 (8H, m, Ar), 7.58–7.25 (12H, m, Ar), 6.79 (1H, d, *J* = 3.2 Hz, NH), 6.60 (1H, t, *J* = 9.1 Hz, H-3), 6.12 (1H, d, *J* = 3.2 Hz, NH), 5.92–5.81 (3H, m, H-2, H-4, -OCH_2_-C*H*=CH_2_), 5.24 (1H, dd, *J* = 17.3, 1.5 Hz, -OCH_2_-CH=C*H_2_*), 5.14 (1H, dd, *J* = 10.9, 1.3 Hz, -OCH_2_-CH=C*H_2_*), 5.13 (1H, m, H-5), 4.73 (1H, dd, *J* = 12.3, 2.8 Hz, H-6a), 4.42 (1H, dd, *J* = 12.3, 3.60 Hz, H-6b), 4.36 (2H, d, *J* = 5.7 Hz, -OC*H_2_*-CH=CH_2_). ^13^C NMR (90 MHz, CDCl_3_) δ (ppm): 169.7 (*C*ONH_2_), 166.2, 165.5, 165.4, 165.1 (4 × *C*=O), 133.6–128.3 (Ar and -OCH_2_-*C*H=CH_2_), 117.9 (-OCH_2_-CH=*C*H_2_), 97.7 (C-1), 72.5, 72.2, 69.7, 68.9 (C-2–C-5), 64.7, 62.7 (C-6, O*C*H_2_-CH=CH_2_).

### 3.15. C-(2,3,4,6-Tetra-O-Acetyl-1-Allyloxy-α-D-Galactopyranosyl)Formamide *(**21**)*

Prepared according to General method I. from **16** (1.0 g, 2.20 mmol) and allyl alcohol. Yield: 513 mg (54%), colorless, white powder. R_f_ = 0.48 (hexane/acetone = 2:1). [α]_D_ = +56 (c = 0.44, CHCl_3_); ^1^H NMR (400 MHz, CDCl_3_) δ (ppm): 6.72 (1H, d, *J* = 3.1 Hz, NH), 6.25 (1H, d, *J* = 3.0 Hz, NH), 5.89 (1H, dd, *J* = 10.4, 3.4 Hz, H-3), 5.96–5.86 (1H, m, -OCH_2_-C*H*=CH_2_), 5.55 (1H, d, *J* = 10.5 Hz, H-2), 5.52 (1H, dd, *J* = 3.2, 1.2 Hz, H-4), 5.29 (1H, dd, *J* = 17.2, 1.5 Hz, -OCH_2_-CH=C*H_2_*), 5.20 (1H, dd, *J* = 10.1, 1.4 Hz, -OCH_2_-CH=C*H_2_*), 4.88 (1H, td, *J* = 7.7, 6.8, 1.2 Hz, H-5), 4.34 (1H, dd, *J* = 12.0, 5.7 Hz, -OC*H_2_*-CH=CH_2_), 4.24 (1H, dd, *J* = 12.0, 6.1 Hz, -OC*H_2_*-CH=CH_2_), 4.13 (1H, dd, *J* = 11.2, 7.0 Hz, H-6a), 4.08 (1H, dd, *J* = 11.2, 6.4 Hz, H-6b), 2.17, 2.07, 2.04, 1.97 (4 × 3H, s, 4 × OCOC*H_3_*). ^13^C NMR (100 MHz, CDCl_3_) δ (ppm): 170.5, 170.0, 169.9, 169.8 (2) (4 × *C*=O), 133.8 (-OCH_2_-*C*H=CH_2_), 117.9 (-OCH_2_-CH=*C*H_2_), 97.6 (C-1), 71.3, 70.0, 67.5, 65.7 (C-2–C-5), 63.9, 61.5 (C-6, -O*C*H_2_-CH=CH_2_), 20.8, 20.8, 20.7, 20.70 (4 × OCO*C*H_3_).

### 3.16. (1′S)-1′,5′-Anhydro-2′,3′,4′,6′-Tetra-O-Benzoyl-D-Glucitol-Spiro-[1′,2]-5-Hydroxymorpholin-3-One *(**22**)*

Prepared according to General method IV. from **20** (540 mg, 0.79 mmol). Yield: 294 mg (56%), colorless, fine powder, diastereomeric ratio (based on the ^1^H NMR integrals): ~70:30. R_f_ = 0.28 (hexane/acetone 2:1). (*Only the peaks of the major diastereomer are listed here, because not all signals of the minor component were separately visible*) ^1^H NMR (400 MHz, CDCl_3_) δ (ppm): 8.06–7.83 (8H, m, Ar), 7.56–7.05 (12H, Ar), 6.74 (1H, t, *J* = 9.8 Hz, H-3′), 5.83 (1H, t, *J* = 9.8 Hz, H-4′), 5.71 (1H, d, *J* = 10.0 Hz, NH), 5.20 (1H, dd, *J* = 8.2, 6.0 Hz, H-5′), 4.95 (1H, d, *J* = 9.8 Hz, H-2′), 4.75 (1H, brs, OH), 4.70 (1H, dd, *J* = 13.1, 3.1 Hz, H-6′a), 4.45 (1H, td, *J* = 12.4, 3.8 Hz, H-5), 4.43 (1H, dd, *J* = 12.3, 3.7 Hz, H-6′b), 4.06 (1H, dd, *J* = 11.3, 8.3 Hz, H-6a), 3.97 (1H, dd, *J* = 11.7, 4.8 Hz, H-6b). ^13^C NMR (100 MHz, CDCl_3_) δ (ppm): 166.8, 166.3, 165.8, 165.4, 165.1 (5 × *C*=O), 133.6–128.4 (Ar), 96.3 (C-1′), 74.0, 73.8, 72.4, 71.9, 69.3 (C-2′–C-5′, C-5), 64.1, 62.9 (C-6, C-6′).

### 3.17. (1′S)-2′,3′,4′,6′-Tetra-O-Acetyl-1′,5′-Anhydro-D-Galactitol-Spiro-[1′,2]-5-Hydroxymorpholin-3-One *(**23**)*

Prepared according to General method IV. from **21** (600 mg, 1.39 mmol). Yield: 270 mg (45%), colorless, fine powder, diastereomeric ratio (based on the ^1^H NMR integrals): ~70:30. R_f_ = 0.21 (hexane/acetone 2:1). (*Only the peaks of the major diastereomer are listed here, because not all signals of the minor component were separately visible*) ^1^H NMR (400 MHz, CDCl_3_) δ (ppm): 7.54 (1H, d, *J* = 6.1 Hz, NH), 5.93 (1H, dd, *J* = 10.6, 3.5 Hz, H-3′), 5.48 (1H, dd, *J* = 3.4, 1.1 Hz, H-4′), 5.38 (1H, d, *J* = 10.6 Hz, H-2′), 5.17 (1H, dd, *J* = 8.2, 5.2 Hz, H-5′), 4.66 (1H, dt, *J* = 7.0, 1.3 Hz, H-5), 4.33 (1H, brs, OH), 4.14 (2H, dd, *J* = 6.6, 3.3 Hz, H-6a,b), 4.00 (1H, dd, *J* = 11.8, 4.9 Hz, H-6′a), 3.92 (1H, dd, *J* = 11.7, 8.5 Hz, H-6′b), 2.18, 2.06, 2.04, 1.97 (4 × 3H, s, 4 × OCOC*H_3_*). ^13^C NMR (100 MHz, CDCl_3_) δ (ppm): 170.8, 170.5, 170.5, 169.5 (4 × *C*=O), 165.4 (C-3), 96.3 (C-1′), 73.9, 71.1, 70.1, 70.0, 67.3 (C-2′–C-5′, C-5), 63.9, 61.6 (C-6, C-6′), 20.9, 20.8 (3) (4 × OCO*C*H_3_).

### 3.18. 5-Hydroxy-2-Phenylmorpholin-3-One *(**25**)*

Prepared according to General method IV. from **24** (2.0 g, 10.4 mmol, for its preparation see supporting information). Column chromatography (hexane/acetone 3:1) of the crude product gave 769 mg (38%) pale yellow oil. R_f_ = 0.25 (hexane/acetone 3:2). *The product contained all 4 possible stereoisomers (two pairs of enantiomers). Two series of peaks are present in the spectra, in a ratio of 5:1. Only the peaks of the major components are listed here, because not all signals of the minor products were separately visible.* ^1^H NMR (400 MHz DMSO-*d_6_*) δ (ppm): 8.56 (1H, s, NH), 7.38–7.30 (5H, m, Ar), 6.17 (1H, d, *J* = 7.8 Hz, OH), 5.03 (1H, s, H-2), 4.94 (1H, dddd, *J* = 7.7, 5.7, 3.7, 2.4 Hz, H-5), 3.84 (1H, dd, *J* = 11.8, 3.6 Hz, H-6a), 3.46 (1H, dd, *J* = 11.8, 5.4 Hz, H-6b). ^13^C NMR (100 MHz, DMSO-*d_6_*) δ (ppm): 168.3 (C-3), 137.4, 128.2, 128.1 (2), 127.9 (2) (Ar), 77.7, 73.3 (C-2, C-5), 66.2 (C-6). HRMS (positive mode, m/z): 216.0630 (calculated value for C_10_H_11_NO_3_Na: 216.0631).

### 3.19. (1′S)-1′,5′-Anhydro-2′,3′,4′,6′-Tetra-O-Benzoyl-D-Glucitol-Spiro-[1′,2]-5-Iodomethyl-Morpholin-3-One *(**26**)*

In a flame dried round bottom flask, allyloxyamide **20** (0.72 g, 1.06 mmol) and iodine (0.81 g, 3 equiv.) were dissolved in dry acetonitrile (20 mL), and dried potassium carbonate (0.44 g, 3 equiv.) was added. The suspension was stirred at room temperature for 4 h, after which TLC showed complete conversion. The mixture was diluted with chloroform (40 mL), extracted with a ~5% aqueous solution of sodium sulfite and twice with water. The organic phase was dried over magnesium sulfate, filtered and concentrated. The product was obtained after column chromatography (hexane/acetone 3:1). Yield: 605 mg (71%), pale yellow oil. R_f_ = 0.38 (hexane/acetone 2:1). (*Only the peaks of the major diastereomer are listed here, because not all signals of the minor component were separately visible* ^1^H NMR (400 MHz CDCl_3_) δ (ppm): 8.06–7.81 (8H, m, Ar), 7.56–7.22 (13H, Ar, NH), 6.74 (1H, t, *J* = 9.7 Hz, H-3′), 5.79 (1H, t, *J* = 9.8 Hz, H-4′), 5.72 (1H, d, *J* = 9.7 Hz, H-2′), 4.98 (1H, dt, *J* = 9.5, 3.7 Hz, H-5′), 4.64 (1H, dd, *J* = 11.9, 2.3 Hz, H-6′a), 4.48 (1H, dd, *J* = 12.2, 4.4 Hz, H-6′b), 4.01–3.92 (2H, m, H-6a,b), 3.68–3.62 (1H, m, H-5), 3.13 (1H, dd, *J* = 10.7, 4.9 Hz, -C*H_2_*I), 3.30 (1H, dd, *J* = 10.8, 6.7 Hz, -C*H_2_*I). ^13^C NMR (100 MHz, CDCl_3_) δ (ppm): 166.3, 165.7, 165.7, 165.4, 165.1 (4 × *C*=O), 133.6–128.3 (Ar), 96.6 (C-1′), 73.4, 72.4, 72.3, 69.5 (C-2′–C-5′), 64.1, 63.1 (C-6, C-6′), 51.9 (C-5), 2.9 (-*C*H_2_I).

### 3.20. (1′S)-1′,5′-Anhydro-2′,3′,4′,6′-Tetra-O-Benzoyl-D-Glucitol-Spiro-[1′,2]-Morpholine-3,5-Dione *(**27**)*

Prepared according to General method V. from **22** (500 mg, 0.73 mmol). Yield: 354 mg (71%), colorless foam. R_f_ = 0.50 (hexane/acetone 2:1). [α]_D_ = +21 (c = 0.51, CHCl_3_); ^1^H NMR (360 MHz CDCl_3_) δ (ppm): 8.29 (1H, s, NH), 8.02–7.80 (8H, m, Ar), 7.80–7.25 (12H, m, Ar), 6.71 (1H, t, *J* = 9.8 Hz, H-3′), 5.85 (1H, d, *J* = 10.1 Hz, H-2′), 5.82 (1H, t, *J* = 9.7 Hz, H-4′), 4.73 (1H, d, *J* = 17.0 Hz, H-6a), 4.69 (1H, dd, *J* = 12.3, 2.7 Hz, H-6′a), 4.50 (1H, ddd, *J* = 9.9, 4.3, 2.9 Hz, H-5′), 4.44 (1H, dd, *J* = 12.7, 4.6 Hz, H-6′b), 4.41 (1H, d, *J* = 17.0 Hz, H-6b). ^13^C NMR (90 MHz, CDCl_3_) δ (ppm): 167.7, 166.2, 165.6, 165.3, 164.9, 164.7 (6 × *C*=O), 133.8–128.4 (Ar), 95.8 (C-1′), 72.5, 72.4, 71.7, 69.0 (C-2′–C-5′), 62.3, 62.1 (C-6, C-6′).

### 3.21. (1′S)-2′,3′,4′,6′-Tetra-O-Acetyl-1′,5′-Anhydro-D-Galactitol-Spiro-[1′,2]-Morpholine-3,5-Dione *(**28**)*

Prepared according to General method V. from **23** (500 mg, 1.16 mmol). Yield: 384 mg (77%), colorless, foam. R_f_ = 0.50 (hexane/acetone 2:1). [α]_D_ = +24 (c = 0.45, CHCl_3_); ^1^H NMR (400 MHz CDCl_3_) δ (ppm): 8.79 (1H, s, NH), 5.91 (1H, dd, *J* = 10.7, 3.5 Hz, H-3′), 5.55 (1H, dd, *J* = 3.4, 1.0 Hz, H-4′), 5.51 (1H, d, *J* = 10.7, H-2′), 4.68 (1H, d, *J* = 17.0 Hz, H-6a), 4.41 (1H, d, *J* = 17.0 Hz, H-6b), 4.30 (1H, td, *J* = 6.5, 1.0 Hz, H-5′), 4.18 (1H, dd, *J* = 11.3, 6.2 Hz, H-6′a), 4.13 (1H, dd, *J* = 11.1, 6.9 Hz, H-6′b), 2.19, 2.08, 2.05, 1.99 (4 × 3H, s, 4 × OCOC*H_3_*). ^13^C NMR (100 MHz, CDCl_3_) δ (ppm): 170.5, 170.2, 169.9, 169.4, 168.1, 164.5 (6 × *C*=O), 95.6 (C-1′), 71.4, 69.4, 69.1, 66.9 (C-2′–C-5′), 61.8, 61.2 (C-6, C-6′), 20.3, 20.7 (2), 20.6 (4 × OCO*C*H_3_).

### 3.22. (1′S)-1′,5′-Anhydro-2′,3′,4′,6′-Tetra-O-Benzoyl-D-Glucitol-Spiro-[1′,2]-(2H-1,4-Oxazin-3[4H]-One) *(**29**)*

Prepared according to General method VI. from **22** (290 mg, 0.42 mmol) Yield: 174 mg (62%), colorless powder. R_f_ = 0.46 (hexane/acetone 2:1). [α]_D_ = +29 (c = 0.50, CHCl_3_); ^1^H NMR (400 MHz DMSO-*d_6_*) δ (ppm): 10.40 (1H, d, *J* = 4.8 Hz, NH), 7.96–7.72 (8H, m, Ar), 7.67–7.34 (12H, m, Ar), 6.68 (1H, t, *J* = 9.6 Hz, H-3′), 6.36 (1H, d, *J* = 3.8 Hz, H-6), 6.00 (1H, t, *J* = 4.4 Hz, H-5), 5.78 (1H, t, *J* = 9.6 Hz, H-4′), 5.73 (1H, d, *J* = 9.9 Hz, H-2′), 4.52–4.45 (3H, m, H-5′, H-6′a, H-6′b). ^13^C NMR (100 MHz, DMSO-*d_6_*) δ (ppm): 165.3, 164.9, 164.7, 164.4 (4 × *C*=O), 158.4 (C-3), 134.0–128.2 (Ar), 125.8 (C-6), 106.7 (C-5), 97.1 (C-1′), 72.6, 72.5, 72.0, 68.8 (C-2′–C-5′), 62.5 (C-6′).

### 3.23. (1′S)-2′,3′,4′,6′-Tetra-O-Acetyl-1′,5′-Anhydro-D-Galactitol-Spiro-[1′,2]-(2H-1,4-Oxazin-3[4H]-One) *(**30**)*

Prepared according to General method VI. from **23** (150 mg, 0.35 mmol). Yield: 86 mg (60%), colorless powder. R_f_ = 0.51 (hexane/acetone 2:1). [α]_D_ = +40 (c = 0.33, CHCl_3_); ^1^H NMR (400 MHz CDCl_3_) δ (ppm): 7.78 (1H, d, *J* = 5.0 Hz, NH), 6.19 (1H, dd, *J* = 4.3, 1.4 Hz, H-6), 6.01 (1H, dd, *J* = 10.7, 3.6 Hz, H-5), 5.82 (1H, t, *J* = 4.8 Hz, H-3′), 5.55–5.52 (2H, m, H-2, H-4′), 4.31 (1H, dt, *J* = 6.6, 1.3 Hz, H-5′), 4.16 (1H, dd, *J* = 10.5, 5.4 Hz, H-6′a), 4.11 (1H, dd, *J* = 10.5, 6.9 Hz, H-6′b), 2.19, 2.08, 2.03, 1.98 (4 × 3H, s, 4 × OCOC*H_3_*). ^13^C NMR (100 MHz, CDCl_3_) δ (ppm): 170.4, 170.3, 169.4, 169.7 (4 × *C*=O), 159.3 (C-3), 126.8 (C-6), 105.6 (C-5), 98.1 (C-1′), 71.9, 70.2, 69.7, 67.1 (C-2′–C-5′), 61.2 (C-6′), 20.9, 20.8, 20.8, 20.7 (4 × OCO*C*H_3_).

### 3.24. 2-Phenylmorpholine-3,5-Dione *(**31**)*

Prepared according to General method V. from **25** (310 mg, 1.60 mmol). Column chromatography (hexane/acetone 3:1) gave 198 mg (65%) pale yellow oil. R_f_ = 0.27 (hexane/acetone 2:1). ^1^H NMR (400 MHz CDCl_3_) δ (ppm): 9.04 (1H, s, NH), 7.43–7.37 (5H, m, Ar), 5.28 (1H, s, H-2), 4.32 (2H, d, *J* = 2.8 Hz, H-6a, H-6b). ^13^C NMR (100 MHz, CDCl_3_) δ (ppm): 170.2, 170.1 (C-3, C-5), 132.9, 129.5, 128.9 (2), 127.7 (2) (Ar), 78.1 (C-2), 64.9 (C-6). HRMS (positive mode, m/z): *[M+Na]^+^ was detected only as a small intensity peak, presumably a methanol molecule was added to the substrate under the MS conditions, therefore we detected the [M+MeOH+Na]^+^ as base peak.* [M+Na]^+^: 214.0474 (calculated value for C_10_H_9_NO_3_Na: 214.0475) and [M+MeOH+Na]^+^: 246.0736 (calculated value for C_11_H_13_NO_4_Na: 246.0737).

### 3.25. 2-Phenyl-2H-1,4-Oxazin-3(4H)-One *(**32**)*

Prepared according to General method VI. from **25** (340 mg, 1.76 mmol) 5-hydroxy-2-phenylmorpholin-3-one and 45 mg (15 mol%) *p*TsOH. Yield: 176 mg (57%), pale yellow oil. R_f_ = 0.41 (hexane/acetone 2:1). ^1^H NMR (360 MHz CDCl_3_) δ (ppm): 8.40 (1H, s, NH), 7.47–7.37 (5H, m, Ar), 6.19 (1H, dd, *J* = 4.2, 1.2 Hz, H-6), 5.68 (1H, t, *J* = 4.4 Hz, H-5), 5.46 (1H, s, H-2). ^13^C NMR (90 MHz, CDCl_3_) δ (ppm): 165.4 (C-3), 135.5, 129.1, 129.0, 128.7 (2), 127.2 (2) (Ar, C-6), 106.5 (C-5), 78.5 (C-2). HRMS (positive mode, m/z): only the dimer [2M+Na]^+^ peak was detected at 373.1145 m/z (calculated value for C_20_H_18_N_2_O_4_Na: 373.1154).

### 3.26. (1′S)-1′,5′-Anhydro-2′,3′,4′,6′-Tetra-O-Benzoyl-D-Glucitol-Spiro-[1′,2]-(5-Methyl-2H-1,4-Oxazin-3[4H]-One) *(**33**)*

In a flame dried round bottom flask, **26** (140 mg, 0.17 mmol) was dissolved in dry THF (5 mL). While stirring, DBU (2 equiv., 52 μL) was added and the solution was stirred at room temperature. The conversion was monitored by TLC (hexane/acetone 2:1). Full conversion was reached after 2 h with minimal amounts of decomposition products. The mixture was quenched with one drop of glacial acetic acid, and the product was obtained after column chromatography (hexane/acetone 3:1). Yield: 60 mg (51%), colorless oil. R_f_ = 0.33 (hexane/acetone 2:1). [α]_D_ = +32 (c = 0.31, CHCl_3_); ^1^H NMR (400 MHz CDCl_3_) δ (ppm): 8.28 (1H, s, NH), 8.03–7.93 (8H, m, Ar), 7.56–7.21 (12H, m, Ar), 6.82 (1H, t, *J* = 9.7 Hz, H-3′), 5.98 (1H, s, H-6), 5.85 (1H, d, *J* = 9.9 Hz, H-2′), 5.80 (1H, t, *J* = 9.8 Hz, H-4′), 4.60–4.53 (2H, m, H-5′, H-6′a), 4.48 (1H, dd, *J* = 11.7, 4.6 Hz, H-6′b), 1.79 (3H, s, -C*H_3_*). ^13^C NMR (100 MHz, CDCl_3_) δ (ppm): 166.2, 165.7, 165.4, 165.1 (4 × *C*=O), 160.1 (C-3), 133.5–128.4 (Ar), 122.4 (C-6), 114.3 (C-5), 97.2 (C-1′), 72.7, 72.6, 72.5, 69.6 (C-2′–C-5′), 63.0 (C-6′), 13.6 (-*C*H_3_). HRMS (positive mode, m/z): 700.1781 (calculated for C_38_H_31_NO_11_Na: 700.1789).

### 3.27. (1′S)-1′,5′-Anhydro-D-Glucitol-Spiro-[1′,2]-Morpholin-3-One *(**34**)*

Prepared according to General method VII. from **13** (60 mg, 90 μmol). Reaction time: 4 h. Column chromatography (CHCl_3_/MeOH 4:1 +0.5% Et_3_N) gave 16 mg (71%) colorless powder. R_f_ = 0.32 (CHCl_3_/MeOH 3:1), [α]_D_ = +27 (c = 0.25, MeOH). Melting point: 178–181 °C. ^1^H NMR (CD_3_OD, 400 MHz) δ (ppm): 4.26 (1H, dt, *J* = 11.9, 3.3 Hz, H-6a), 4.24 (1H, t, *J* = 9.2 Hz, H-3′), 4.04 (1H, ddd, *J* = 9.9, 5.7, 2.3 Hz, H-5′), 3.82 (2H, dd, *J* = 11.9, 2.6 Hz, H-6b, H-6′a), 3.63 (1H, dd, *J* = 12.9, 5.7 Hz, H-6′b), 3.55 (1H, td, *J* = 12.2, 4.4 Hz, H-5a), 3.28 (*partially merged together with the solvent peak*, 2H, *J* = 9.5 Hz, H-2′, H-4′), 3.17 (1H, dd, *J* = 12.5, 2.3 Hz, H-5b). ^13^C NMR (CD_3_OD, 100 MHz) δ (ppm): 168.5 (C-3), 99.7 (C-1′), 77.9, 77.6, 74.5, 71.5 (C-2′–C-5′), 63.2 (C-6′), 59.9 (C-6), 41.9 (C-5). HRMS (positive mode, m/z): 272.0739 (calculated value for C_9_H_15_NO_7_Na: 272.0741).

### 3.28. (1′S)-1′,5′-Anhydro-D-Galactitol-Spiro-[1′,2]-Morpholin-3-One *(**35**)*

Prepared according to General method VII. from **14** (44 mg, 0.105 mmol). Reaction time: 2 h. Column chromatography (CHCl_3_/MeOH 4:1 +0.5% Et_3_N) gave 8 mg (31%) colorless powder. R_f_ = 0.24 (CHCl_3_/MeOH 3:1), [α]_D_ = +52 (c = 0.14, MeOH). Melting point: 142–145 °C. ^1^H NMR (CD_3_OD, 400 MHz) δ (ppm): 4.36 (1H, dd, *J* = 9.9, 3.4 Hz, H-3′), 4.29 (1H, td, *J* = 5.8, 1.1 Hz, H-6a), 4.26 (1H, td, *J* = 11.6, 3.2 Hz, H-6b), 3.89 (1H, dd, *J* = 3.3, 1.1 Hz, H-4′), 3.82 (1H, ddd, *J* = 11.7, 3.5, 0.9 Hz, H-5′), 3.72–3.66 (3H, m, H-2′, H-6′a, H-6′b), 3.55 (1H, td, *J* = 12.2, 4.4 Hz, H-5a), 3.16 (1H, ddd, *J* = 12.5, 2.2, 0.9 Hz, H-5b). ^13^C NMR (CD_3_OD, 100 MHz) δ (ppm): 168.7 (C-3), 100.2 (C-1′), 77.0, 74.4, 72.5, 70.3 (C-2′–C-5′), 63.0, 59.8 (C-6, C-6′), 42.0 (C-5). HRMS (positive mode, m/z): 272.0740 (calculated value for C_9_H_15_NO_7_Na: 272.0741).

### 3.29. (1′S)-1′,5′-Anhydro-D-Glucitol-Spiro-[1′,2]-5-Hydroxymorpholin-3-One *(**36**)*

Prepared according to General method VII. from **22** (75 mg, 0.11 mmol). Reaction time: 2 h. Column chromatography (CHCl_3_/MeOH 4:1 +0.5% Et_3_N) gave 20 mg (68%) colorless powder. R_f_ = 0.35 (CHCl_3_/MeOH 1:1). (*As not all signals of the minor diastereomer were separately visible in the NMR spectra, we are only listing the peaks of the major product*). ^1^H NMR (CD_3_OD, 400 MHz) δ (ppm): 4.37 (1H, dd, *J* = 12.3, 1.9 Hz, H-3′), 4.26 (1H, t, *J* = 8.9 Hz, H-5), 3.96 (1H, ddd, *J* = 9.6, 5.6, 1.9 Hz, H-5′), 3.82 (1H, dd, *J* = 11.9, 1.9 Hz, H-4′), 3.74 (1H, d, *J* = 12.2 Hz, H-2′), 3.62 (1H, dd, *J* = 12.0, 5.8 Hz, H-6′a), 3.38 (1H, d, *J* = 9.5 Hz, H-6a), 3.35–3.29 (2H, m, H-6b, H-6′b). ^13^C NMR (CD_3_OD, 100 MHz) δ (ppm): 167.5 (C-3), 99.2 (C-1′), 77.7, 77.5, 75.4, 74.9, 71.4 (C-5, C-2′–C-5′), 65.3, 62.9 (C-6, C-6′). HRMS (negative mode, m/z): 264.0725, (calculated value for C_9_H_14_NO_8_: 264.0725).

### 3.30. (1′S)-1′,5′-Anhydro-D-Galactitol-Spiro-[1′,2]-5-Hydroxymorpholin-3-One *(**37**)*

Prepared according to General method VII. from **23** (70 mg, 0.16 mmol). Reaction time: 2 h. Column chromatography (CHCl_3_/MeOH 4:1 +0.5% Et_3_N) gave 24 mg (55%) colorless powder. R_f_ = 0.16 (CHCl_3_/MeOH 3:1). (*As not all signals of the minor diastereomer were separately visible in the NMR spectra, we are only listing the peaks of the major product*) ^1^H NMR (CD_3_OD, 360 MHz) δ (ppm): 4.40 (2H, dd, *J* = 12.7, 2.9 Hz, H-6a, H-6b), 4.23 (1H, t, *J* = 6.0 Hz, H-3′), 3.91–3.90 (2H, m, H-5, H-5′), 3.78–3.71 (4H, m, H-2′, H-4′, H-6′a, H-6′b). ^13^C NMR (CD_3_OD, 90 MHz) δ (ppm): 166.2 (C-3), 98.4 (C-1′), 75.5, 73.2, 73.1, 70.9, 68.8 (C-5, C-2′–C-5′), 63.8, 61.9 (C-6, C-6′). HRMS (positive mode, m/z): 288.0690 (calculated value for C_9_H_15_NO_8_Na: 288.0690).

### 3.31. (1′S)-1′,5′-Anhydro-D-Glucitol-Spiro-[1′,2]-Morpholine-3,5-Dione *(**38**)*

Prepared according to General method VII. from **27** (200 mg, 0.29 mmol). Reaction time: 4 h. Column chromatography (CHCl_3_/MeOH 6:1 +0.5% Et_3_N) gave 40 mg (52%) colorless powder. R_f_ = 0.25 (CHCl_3_/MeOH 3:1), [α]_D_ = +45 (c = 0.13, MeOH). Melting point: 186–188 °C. ^1^H NMR (CD_3_OD, 400 MHz) δ (ppm): 4.98 (1H, d, *J* = 16.7 Hz, H-6a), 4.53 (1H, d, *J* = 16.7 Hz, H-6b), 4.49 (1H, t, *J* = 9.3 Hz, H-3′), 4.07 (1H, d, *J* = 9.8 Hz, H-2′), 3.88–3.82 (2H, m, H-4′, H-6′a), 3.66 (1H, d, *J* = 9.6 Hz, H-6′b), 3.53 (1H, ddd, *J* = 9.5, 5.2, 1.6 Hz, H-5′). ^13^C NMR (CD_3_OD, 100 MHz) δ (ppm): 171.7, 167.9 (C-3, C-5), 97.6 (C-1′), 77.7, 76.8, 76.3, 71.3 (C-2′–C-5′), 62.7, 62.6 (C-6, C-6′). HRMS (positive mode, m/z): 286.0535 (calculated value for C_9_H_13_NO_8_Na: 286.0535).

### 3.32. (1′S)-1′,5′-Anhydro-D-Galactitol-Spiro-[1′,2]-Morpholine-3,5-Dione *(**39**)*

Prepared according to General method VII. from **28** (134 mg, 0.31 mmol). Reaction time: 4 h. Column chromatography (CHCl_3_/MeOH 4:1 +0.5% Et_3_N) gave 69 mg (84%) colorless powder. R_f_ = 0.27 (CHCl_3_/MeOH 4:1). ^1^H NMR (CD_3_OD, 400 MHz) δ (ppm): 4.97 (1H, d, *J* = 16.7 Hz, H-6a), 4.61 (1H, dd, *J* = 9.7, 3.5 Hz, H-3′), 4.52 (1H, d, *J* = 16.7 Hz, H-6b), 4.14–4.08 (2H, m, H-4′, H-5′), 4.03–3.89 (3H, m, H-2′, H-6′a, H-6′b). ^13^C NMR (CD_3_OD, 100 MHz) δ (ppm): 171.8, 168.1 (C-3, C-5), 98.1 (C-1′), 76.9, 73.6, 72.1, 70.1 (C-2′–C-5′), 62.9, 62.5 (C-6, C-6′). HRMS (positive mode, m/z): 286.0533 (calculated value for C_9_H_13_NO_8_Na: 286.0533).

### 3.33. (1′S)-1′,5′-Anhydro-D-Glucitol-Spiro-[1′,2]-(2H-1,4-Oxazin-3[4H]-One) *(**40**)*

Prepared according to General method VII. from **29** (112 mg, 0.17 mmol). Reaction time: 3 h. Column chromatography (CHCl_3_/MeOH 6:1 +0.5% Et_3_N) gave 81 mg (72%) colorless powder. R_f_ = 0.32 (CHCl_3_/MeOH 4:1), [α]_D_ = +26 (c = 0.30, MeOH). Melting point: 176–177 °C ^1^H NMR (CD_3_OD, 400 MHz) δ (ppm): 6.16 (1H, d, *J* = 4.3 Hz, H-6), 5.78 (1H, d, *J* = 4.3 Hz, H-5), 4.31 (1H, t, *J* = 9.2 Hz, H-3′), 3.74 (1H, dd, *J* = 12.0, 2.2 Hz, H-6′a), 3.62 (1H, dd, *J* = 12.0, 4.9 Hz, H-6′b), 3.56 (1H, ddd, *J* = 9.8, 4.9, 2.2 Hz, H-5′), 3.41 (1H, d, *J* = 9.6 Hz, H-2′), 3.36 (1H, t, *J* = 9.3 Hz, H-4′). ^13^C NMR (CD_3_OD, 100 MHz) δ (ppm): 162.1 (C-3), 127.6 (C-6), 106.8 (C-5), 100.1 (C-1′), 78.2, 77.1, 75.9, 71.2 (C-2′–C-5′), 62.4 (C-6′). HRMS (positive mode, m/z): 270.0581 (calculated value for C_9_H_13_NO_7_Na: 270.0584).

### 3.34. (1′S)-1′,5′-Anhydro-D-Galactitol-Spiro-[1′,2]-(2H-1,4-Oxazin-3[4H]-One) *(**41**)*

Prepared according to General method VII. from **30** (76 mg, 0.18 mmol). Reaction time: 2 hours. Column chromatography (CHCl_3_/MeOH 3:1 +0.5% Et_3_N) gave 32 mg (71%) colorless powder. R_f_ = 0.20 (CHCl_3_/MeOH 3:1), [α]_D_ = +62 (c = 0.21, MeOH). Melting point: 132–136 °C. ^1^H NMR (CD_3_OD, 400 MHz) δ (ppm): 6.21 (1H, d, *J* = 4.3 Hz, H-6), 5.81 (1H, d, *J* = 4.3 Hz, H-5), 4.88 (1H, dd, *J* = 9.9, 3.6 Hz, H-3′), 3.95 (1H, dd, *J* = 3.8, 1.0. Hz, H-4′), 3.86 (1H, td, *J* = 6.0, 1.0 Hz, H-5′), 3.83 (1H, d, *J* = 10.0 Hz, H-2′), 3.75–3.66 (2H, m, H-6′a, H-6′b). ^13^C NMR (CD_3_OD, 100 MHz) δ (ppm): 162.2 (C-3), 127.9 (C-6), 106.7 (C-5), 100.6 (C-1′), 77.4, 74.2, 72.8, 70.0 (C-2′–C-5′), 62.4 (C-6′). HRMS (positive mode, m/z): 270.0584 (calculated value for C_9_H_13_NO_7_Na: 270.0584).

### 3.35. (1′S)-1′,5′-Anhydro-D-Glucitol-Spiro-[1′,2]-(5-Methyl-2H-1,4-Oxazin-3[4H]-One) *(**42**)*

Prepared according to General method VII. from **33** (85 mg, 0.125 mmol). Reaction time: 3 hours. Column chromatography (CHCl_3_/MeOH 6:1 +0.5% Et_3_N) gave 30 mg (77%) colorless powder. R_f_ = 0.25 (CHCl_3_/MeOH 6:1), [α]_D_ = +31 (c = 0.26, MeOH). Melting point: 162–164 °C. ^1^H NMR (CD_3_OD, 400 MHz) δ (ppm): 5.97 (1H, d, *J* = 1.4 Hz, H-6), 4.37 (1H, t, *J* = 9.2 Hz, H-3′), 3.79 (1H, dd, *J* = 11.9, 2.0 Hz, H-6′a), 3.67 (1H, dd, *J* = 11.9, 5.0 Hz, H-6′b), 3.61 (1H, ddd, *J* = 9.9, 5.0, 2.2 Hz, H-5′), 3.44 (1H, d, *J* = 9.6 Hz, H-2′), 3.40 (1H, t, *J* = 8.5 Hz, H-4′), 1.75 (3H, d, ^4^*J*_H-2, Me_ =1.32 Hz, C*H_3_*). ^13^C NMR (DMSO-*d_6_*, 100 MHz) δ (ppm): 161.1 (C-3), 121.7 (C-6), 114.4 (C-5), 98.2 (C-1′), 77.6, 75.9, 74.5, 70.1 (C-2′–C-5′), 61.3 (C-6′), 13.5 (-*C*H_3_). HRMS (positive mode, m/z): 284.0738 (calculated value for C_10_H_15_NO_7_Na: 284.0741).

## 4. Conclusions

This systematic study of the synthetic possibilities to obtain glycopyranosylidene-spiro-morpholinones revealed that (glyculosylbromide)onamides are suitable starting materials for the (*S*) spiro-epimers via the corresponding (2-chloroethyl and allyl glyculoside)onamides to be obtained by Ag(I) promoted glycosylations. Attempts to prepare the corresponding glycosides to obtain the (*R*) spiro-epimers remained unsuccessful due to the failure of alkylation reactions of the highly hindered glycosidic hydroxyl group of glyculosonamides. The allyl glycosides’ (in general, α-allyloxy-carboxamides’) ring closure by ozonolysis represents a new method for the construction of morpholinone rings as their diastereomeric 5-hydroxy derivatives. Another cyclization of the allyl glycosides by iodine resulted in 5-iodomethyl-morpholinone diastereomers. The new stereogenic center in these compounds was abolished by oxidation and elimination reactions that furnished stereochemically uniform spiro-morpholin-3,5-diones, 5,6-didehydro-morpholin-3-ones and 5-methyl-2*H*-1,4-oxazin-3(4*H*)-ones.

## Supplementary Materials

The following supporting information can be downloaded at: https://www.mdpi.com/article/10.3390/molecules27227785/s1, Synthesis of 2-allyloxy-2-phenylacetamide (**24**); attempted transformations of glucopyranosonamide **18**; copies of the ^1^H and ^13^C J-MOD NMR spectra.

## Sample Availability

Samples of the compounds are not available from the authors.
