# Supplementary material for: Glycopyranosylidene-Spiro-Morpholinones: Evaluation of the Synthetic Possibilities Based on Glyculosonamide Derivatives and a New Method for the Construction of the Morpholine Ring
Source: Molecules. 2022 Nov 11;27(22):7785. doi: 10.3390/molecules27227785 (PMC9698030; doi:10.3390/molecules27227785)
Supplement: Supplementary file 1 [file molecules-27-07785-s001.zip › molecules-1988799-supplementary.pdf]

molecules

Supplementary Materials

Glycopyranosylidene-Spiro-Morpholinones: Evaluation of the Synthetic Possibilities Based on Glyculosonamide Derivatives and a New Method for the Construction of the Morpholine Ring

Nándor Kánya, Sándor Kun and László Somsák *

Contents

Synthesis of 2-allyloxy-2-phenylacetamide (24)..2

Attempted transformations of glucopyranosonamide 18..3

Copies of the ^{1}H and ^{13}C J-MOD NMR spectra 4

Synthesis of 2-allyloxy-2-phenylacetamide (24)

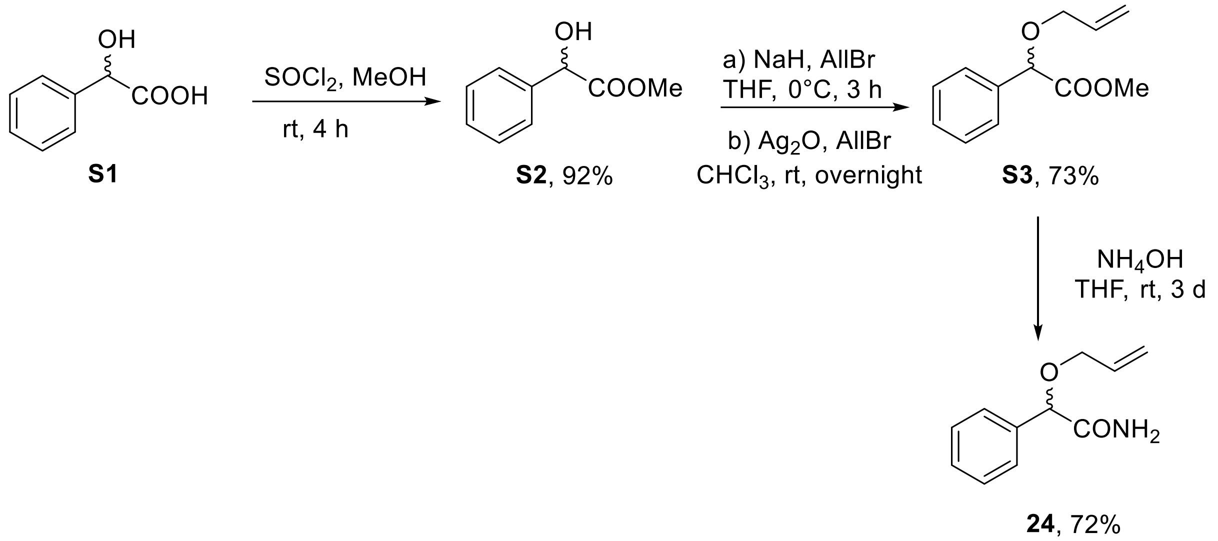

This synthesis was performed from racemic mandelic acid **S1** via the known intermediates **S2** and **S3** prepared according to a published procedure (Vink, M.K.S., PhD Thesis, University of Amsterdam, 2003. https://dare.uva.nl/search?identifier=7012b66e-2c8b-48f7-8258-d284d5e8ffde)

2-Allyloxy-2-phenylacetamide (24)

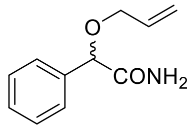

Methyl 2-(allyloxy)-2-phenylacetate (**S3**, 3.0 g, 14.5 mmol) was dissolved in THF (15 mL), and aqueous ammonia solution (25 w/w %, 25 mL) was added. The resulting two-phase system was stirred vigorously at room temperature for 2 days, after which TLC showed complete conversion. The mixture was diluted with water (40 mL), and the aqueous layer was extracted with ethyl acetate (3 × 20 mL), and the combined organic phases was dried on magnesium sulphate, filtered and concentrated. The crude product was used without further purification. Yield: 2.0 g (72 %), pale yellow oil. R_f = 0.38 (hexane/acetone 2:1). ^{1}H NMR (400 MHz $CDCl_3$) δ (ppm): 7.42-7.29 (5H, m, Ar), 6.72 (1H, brs, NH), 6.63 (1H, brs, NH), 5.89 (1H, dddd, *J* = 17.2, 10.5, 6.0, 5.4 Hz, -OCH$_2$-C*H*=CH$_2$), 5,26 (1H, dq, *J* = 17.3, 1.6, 1.6 Hz, -OCH$_2$-CH=C*H*$_2$), 5.21 (1H, dq, *J* = 10.4, 1.2, Hz, -OCH$_2$-CH=C*H*$_2$), 4.76 (1H, s, Ph-C*H*-O), 4.03 (1H, ddt, *J* = 12.7, 5.2, 1.4 Hz, -OC*H*$_2$-CH=CH$_2$), 3.94 (1H, ddt, *J* = 12.8, 6.0, 1.3 Hz, -OC*H*$_2$-CH=CH$_2$). ^{13}C NMR (100 MHz, $CDCl_3$) δ (ppm): 174.0 (CONH$_2$), 137.0, 133.7, 128.6 (2), 128.6, 127.2 (2) (Ar, -OCH$_2$-CH=CH$_2$), 118.0 (-OCH$_2$-CH=CH$_2$), 81.1 (Ph-CH-O), 70.2 (-OCH$_2$-CH=CH$_2$).

Attempted transformations of glucopyranosonamide 18

Table S1. Attempted transformations of **18** with dielectrophiles

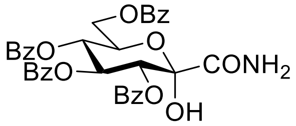

18

Reagent	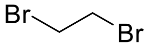	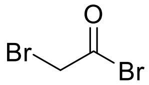
Conditions		
NaH/THF/25°C	Decomposition	Decomposition
K_2CO_3/MeCN/100°C	No reaction	No reaction
Pyridine/100°C	No reaction	No reaction

Table S2. Unsuccesful experiments for the allylation of **18**

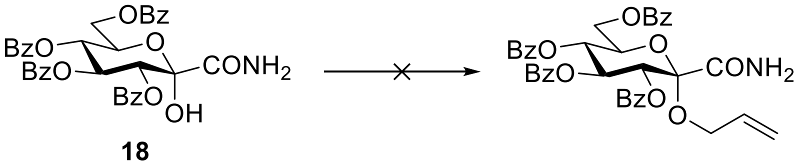

18

Conditions	Observation
Allyl bromide/NaH//THF/0°C	Decomposition
1. Allyl alcohol + Tf_2O/0°C, 2. **18** + K_2CO_3	No reaction
Allyl bromide + AgOTf/Et_3N/$CHCl_3$/25°C	No reaction
1. Allyl alcohol + Cl_3C-CN/NaH/Et_2O/0°C<br>2. **18** + K_2CO_3	No reaction
Allyl bromide + $Pd(dba)_3$/$CHCl_3$/25°C<br>„Tsuji-Trost allylation"{Trost, 1996 #6396}	No reaction
1. Me_3Si-NEt_2, 2. Allyl-bromide +<br>CsF/DMF/0°C{Suzuki, 2015 #6397}	No reaction

Copies of the ¹H and ¹³C J-MOD NMR spectra

¹H (400 MHz) and ¹³C J-MOD (100 MHz) NMR spectra of compound **11** in $CDCl_3$

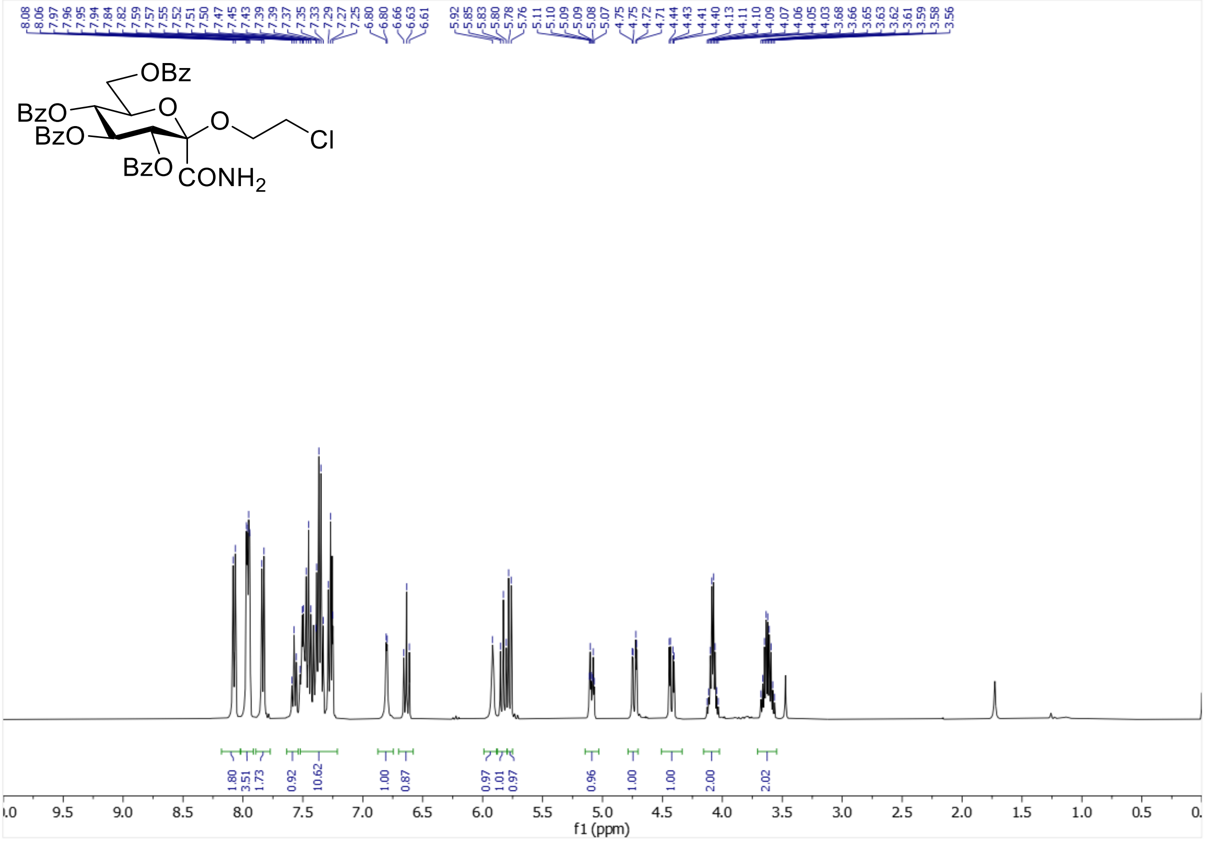

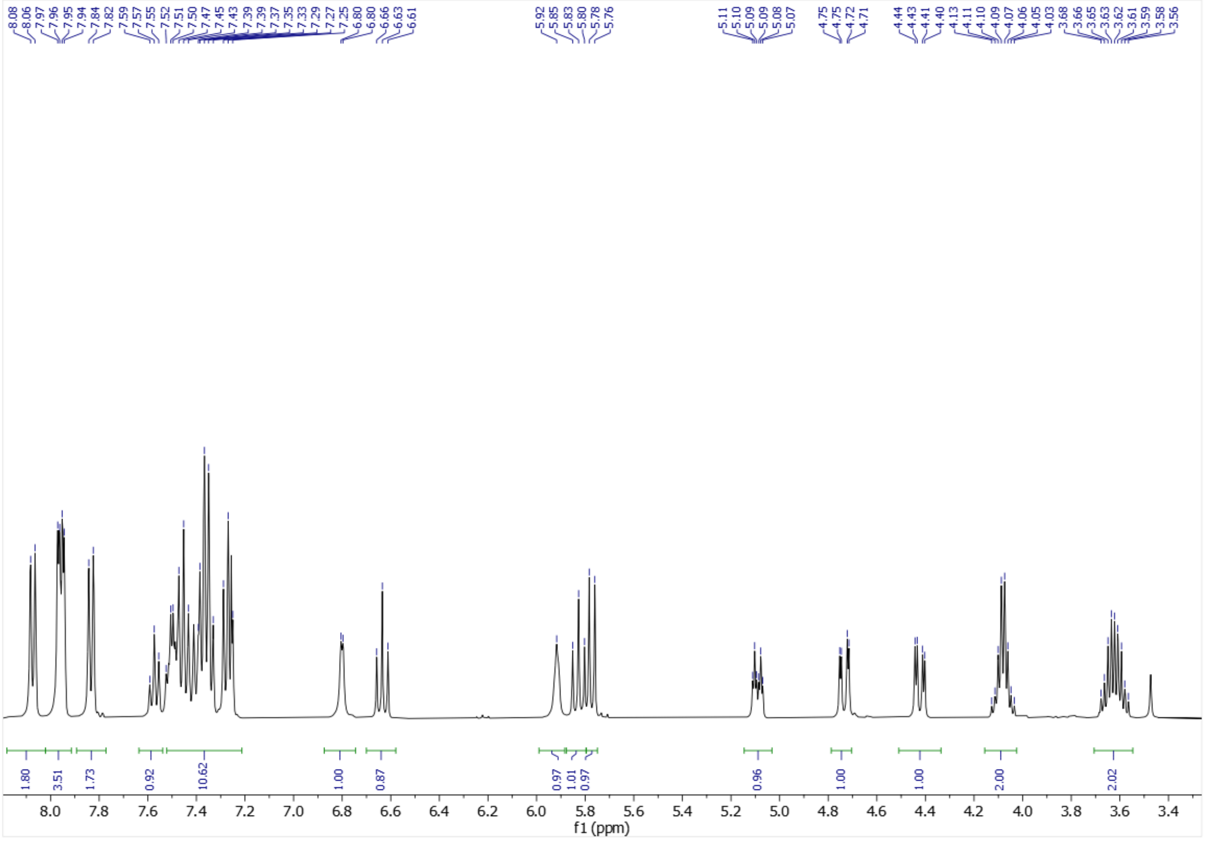

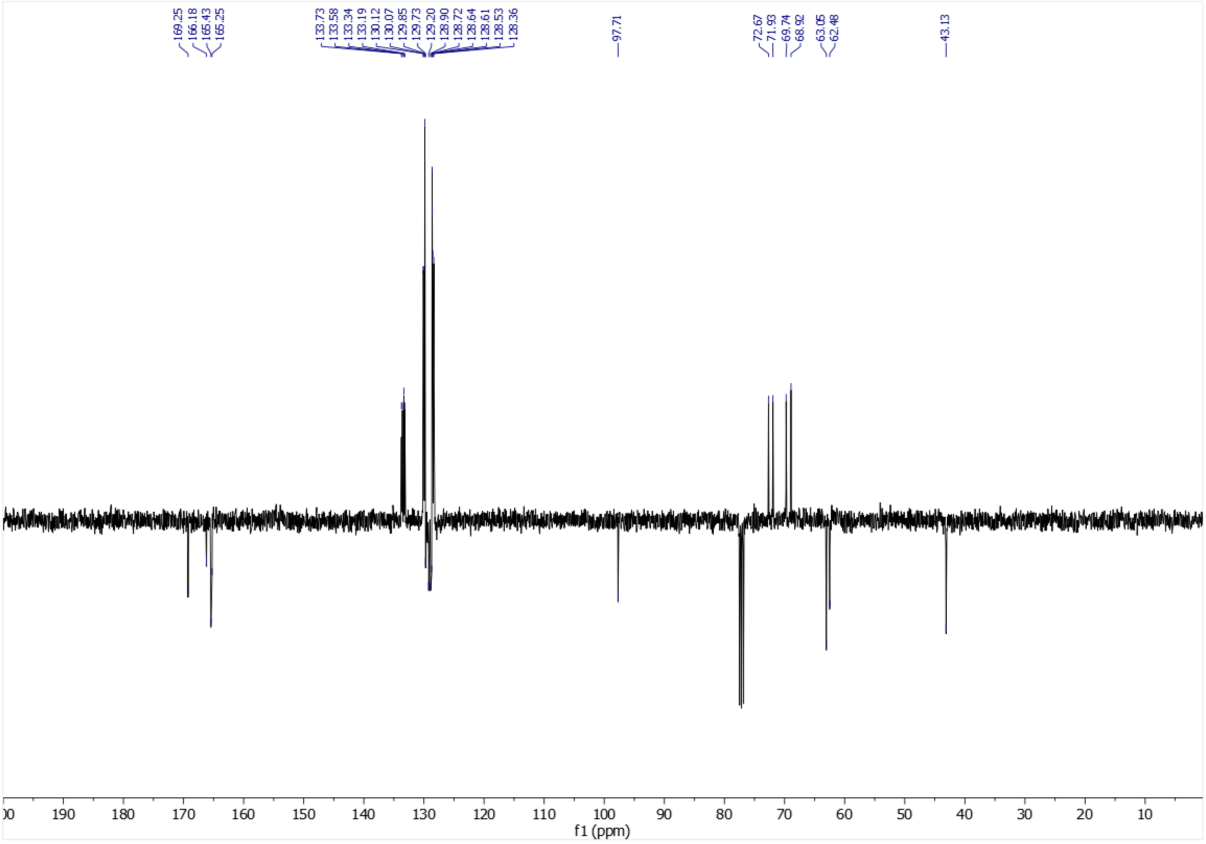
169.25
166.18
165.43
165.25
133.73
133.58
133.34
133.19
130.12
130.07
129.85
129.73
129.20
128.90
128.72
128.64
128.61
128.53
128.36
97.71
72.67
71.93
69.74
68.92
63.05
62.48
43.13
190 180 170 160 150 140 130 120 110 100 90 80 70 60 50 40 30 20 10
f1 (ppm)

^{1}H (400 MHz) and ^{13}C J-MOD (100 MHz) NMR spectra of compound **12** in $CDCl_3$

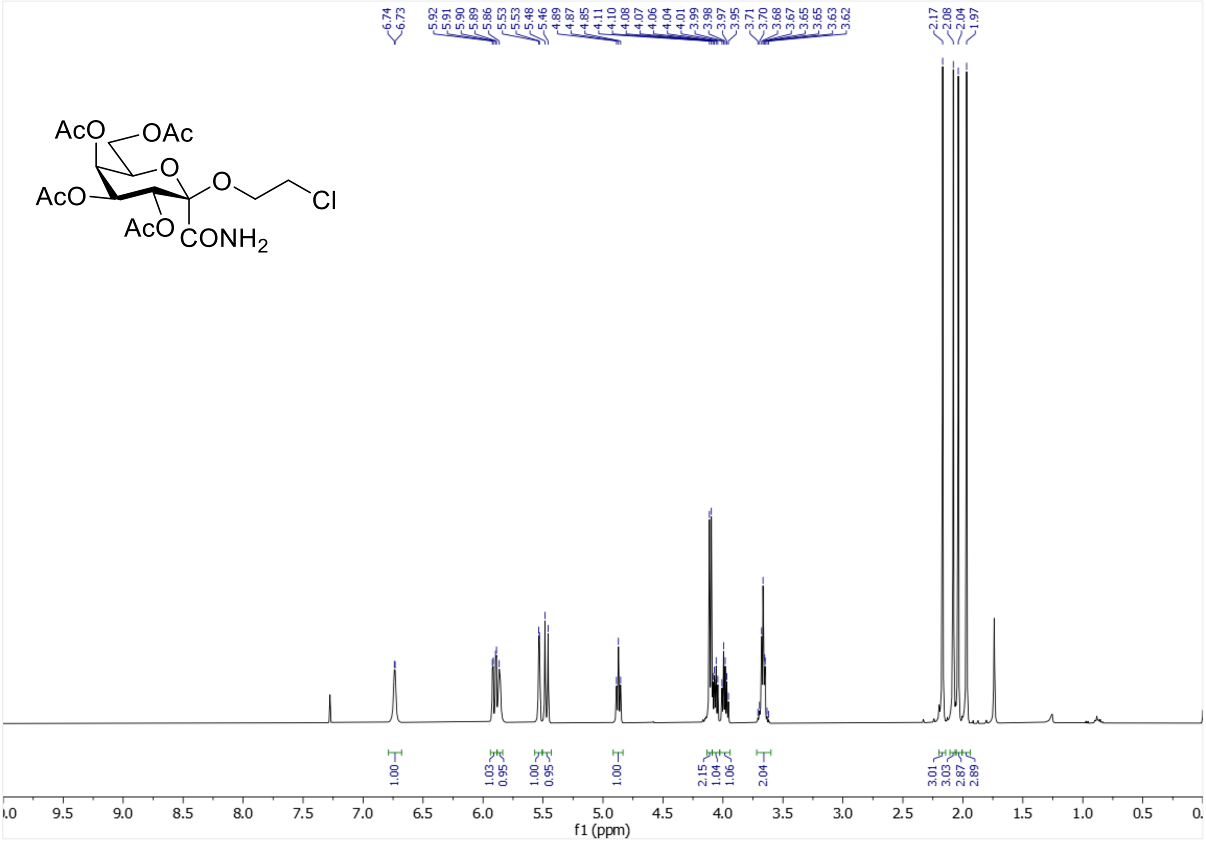

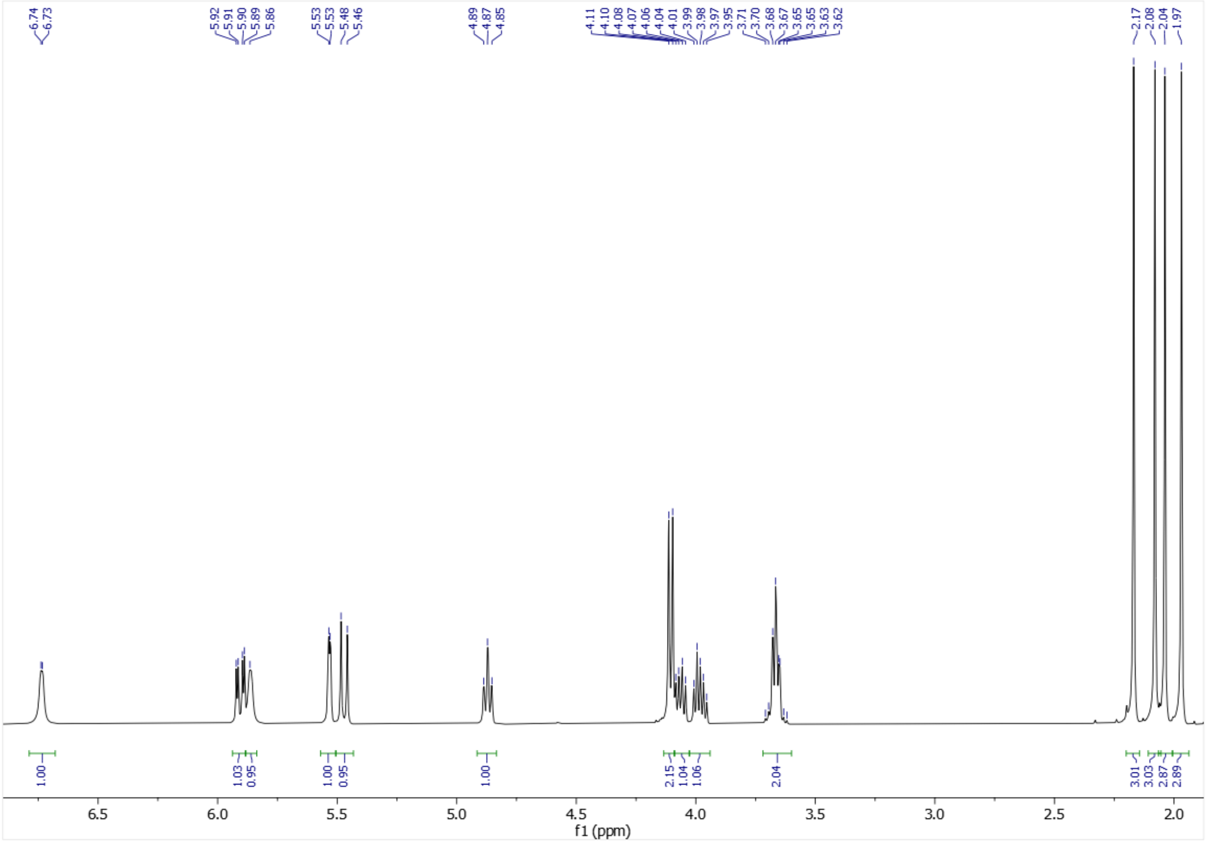

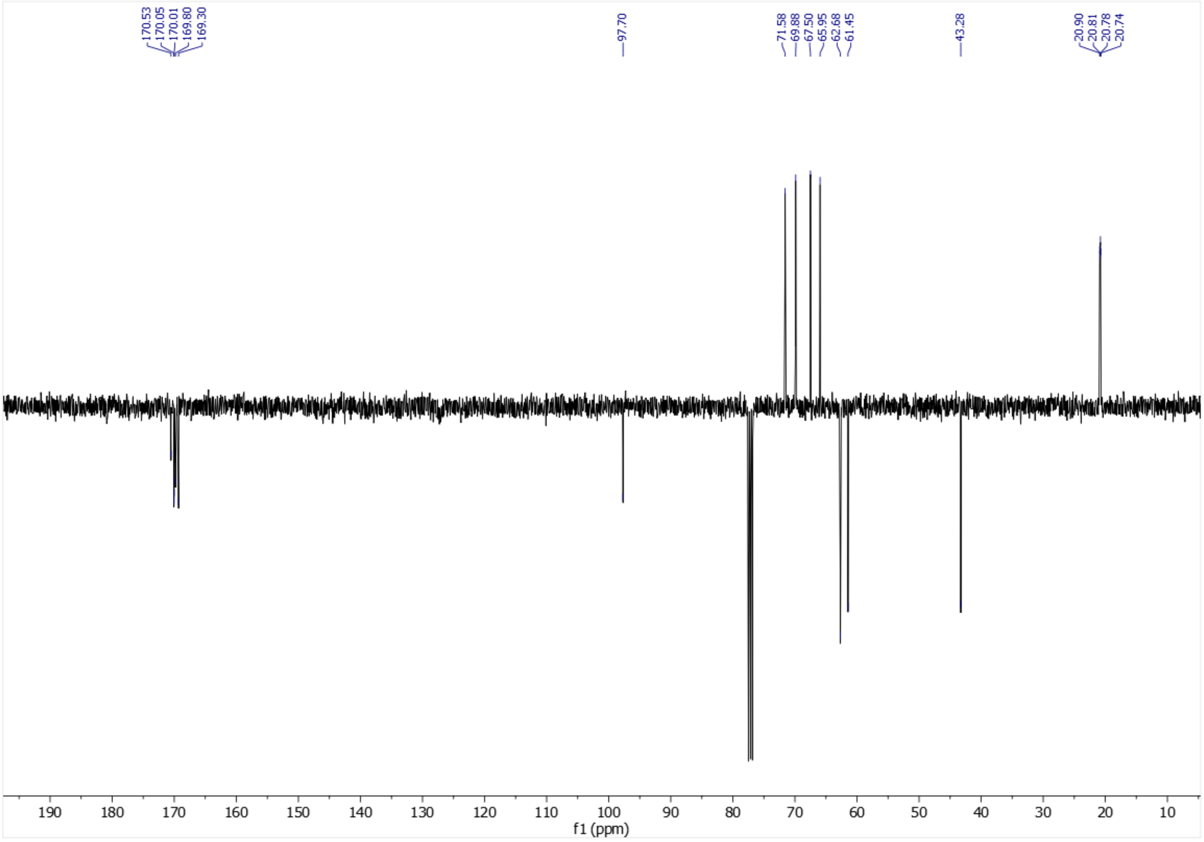
170.53
170.05
170.01
169.80
169.30
97.70
71.58
69.88
67.50
65.95
62.68
61.45
43.28
20.90
20.81
20.78
20.74
190
180
170
160
150
140
130
120
110
100
90
80
70
60
50
40
30
20
10
f1 (ppm)

^{1}H (400 MHz) and ^{13}C J-MOD (100 MHz) NMR spectra of compound **13** in $CDCl_3$

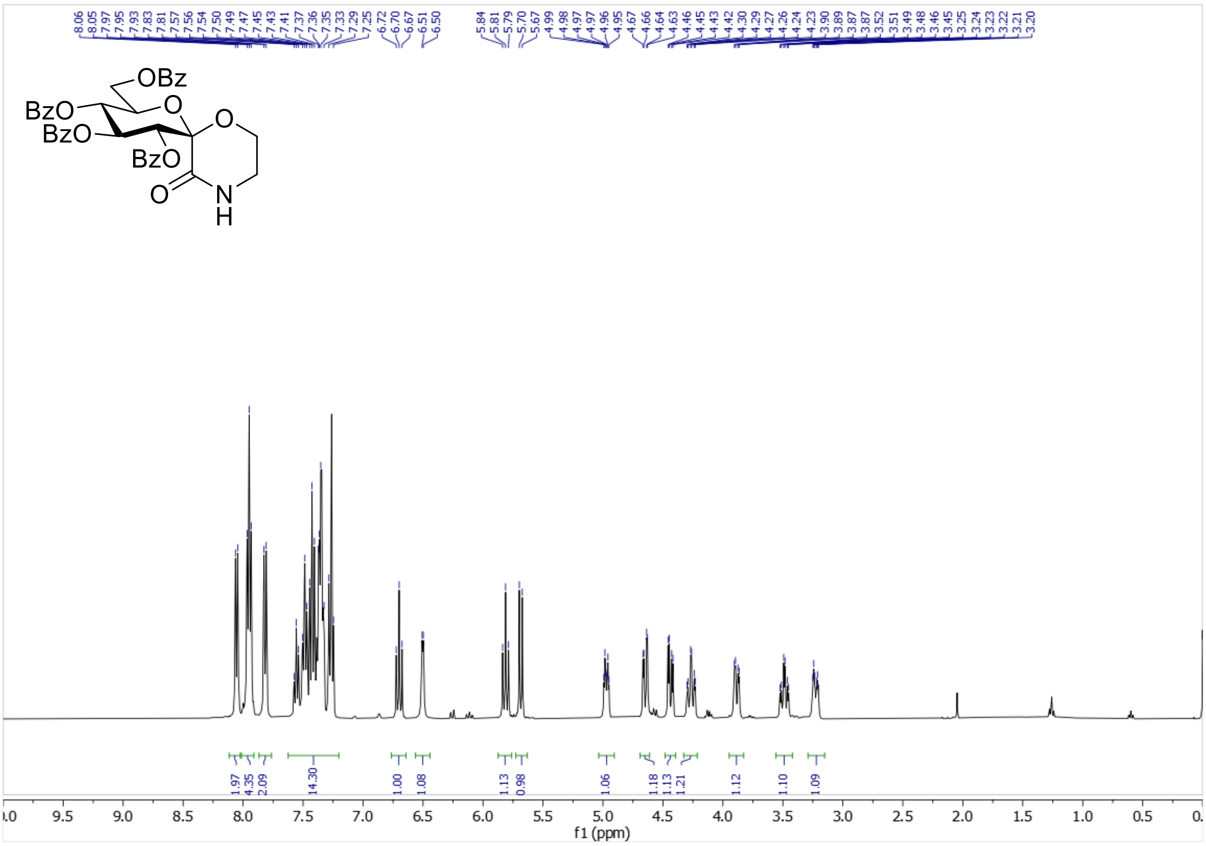

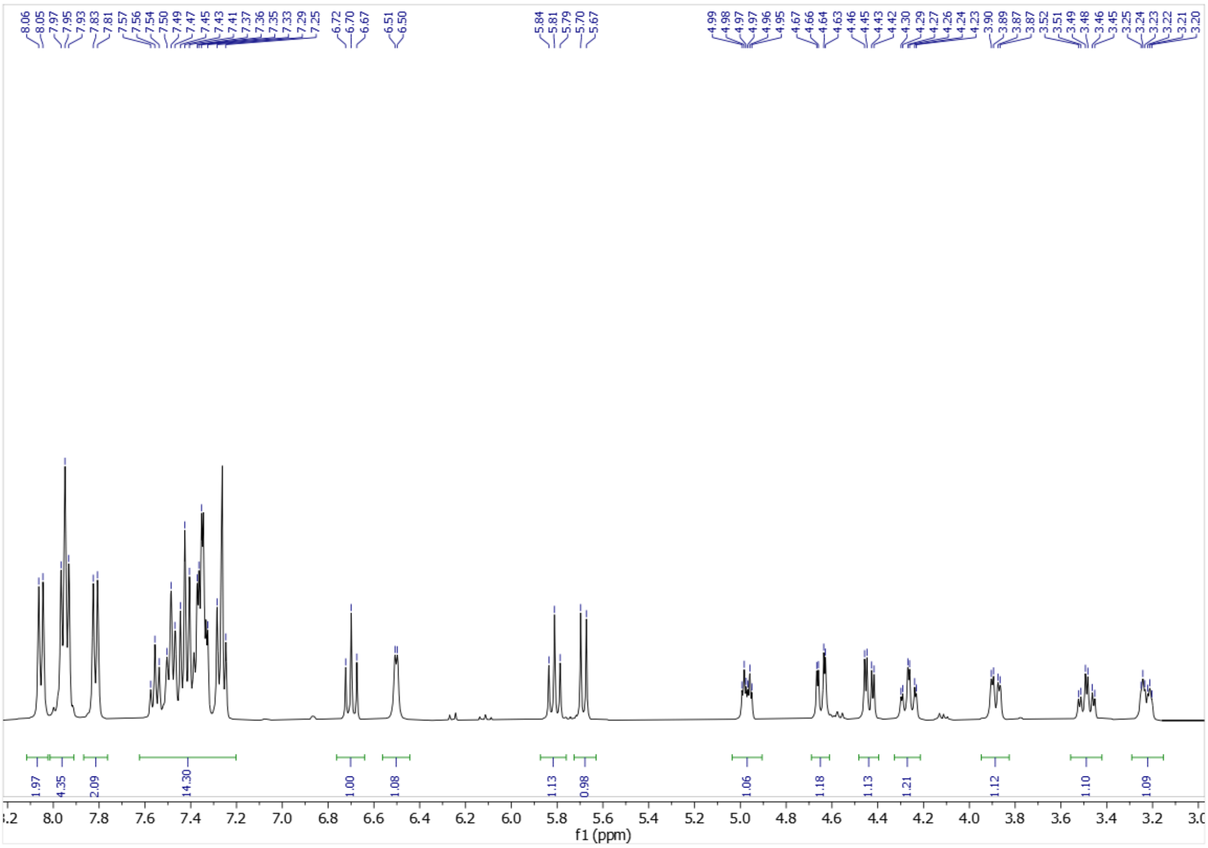

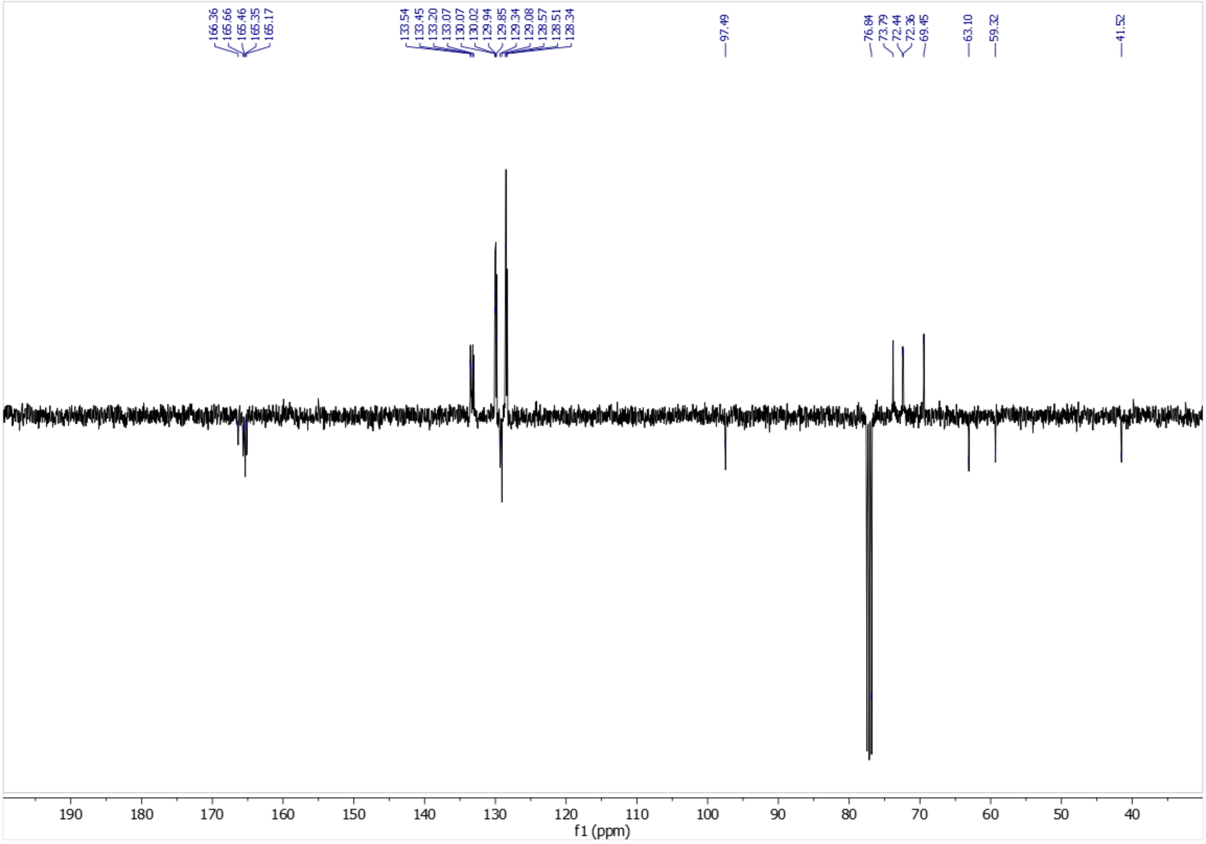
166.36
165.66
165.46
165.35
165.17
133.54
133.45
133.20
133.07
130.07
130.02
129.94
129.85
129.34
129.08
128.57
128.51
128.34
97.49
76.84
73.79
72.44
72.36
69.45
63.10
59.32
41.52
190 180 170 160 150 140 130 120 110 100 90 80 70 60 50 40
f1 (ppm)

^{1}H (400 MHz) and ^{13}C J-MOD (100 MHz) NMR spectra of compound **14** in $CDCl_3$

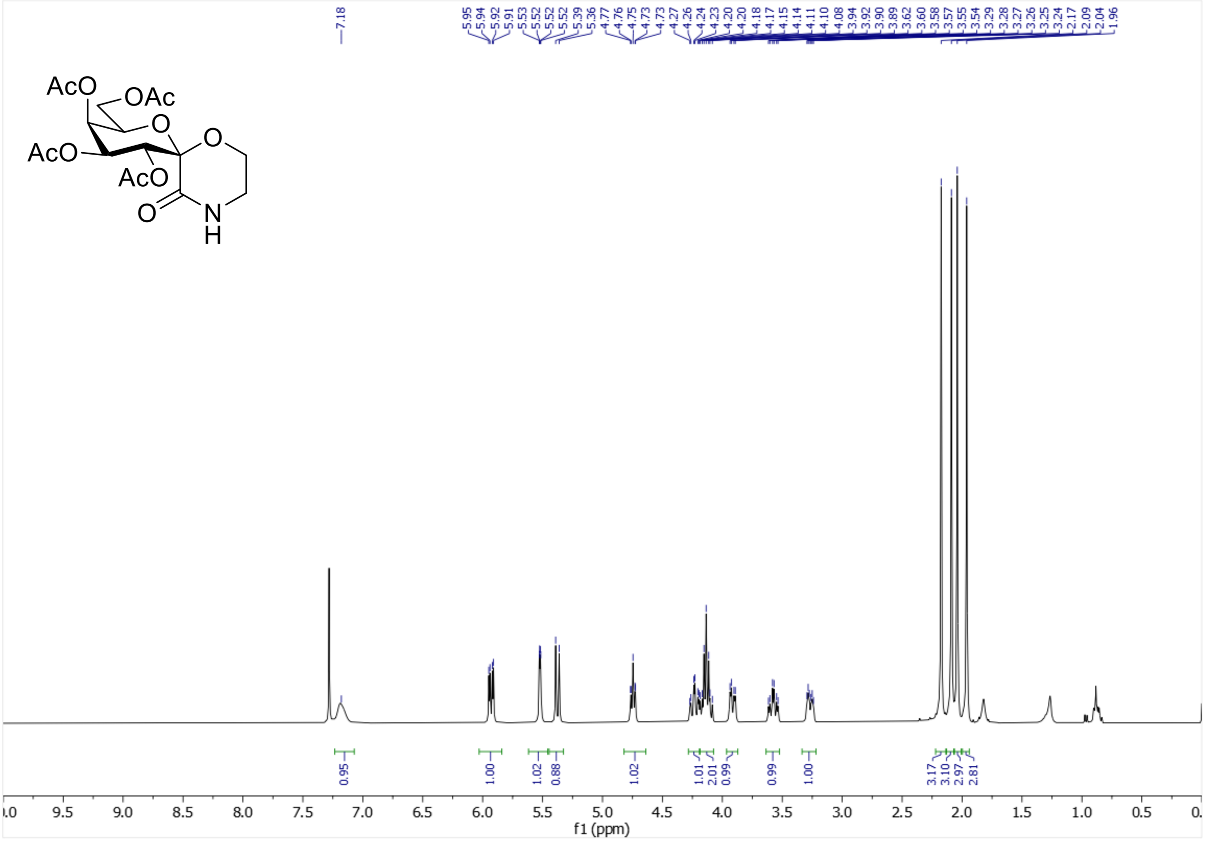

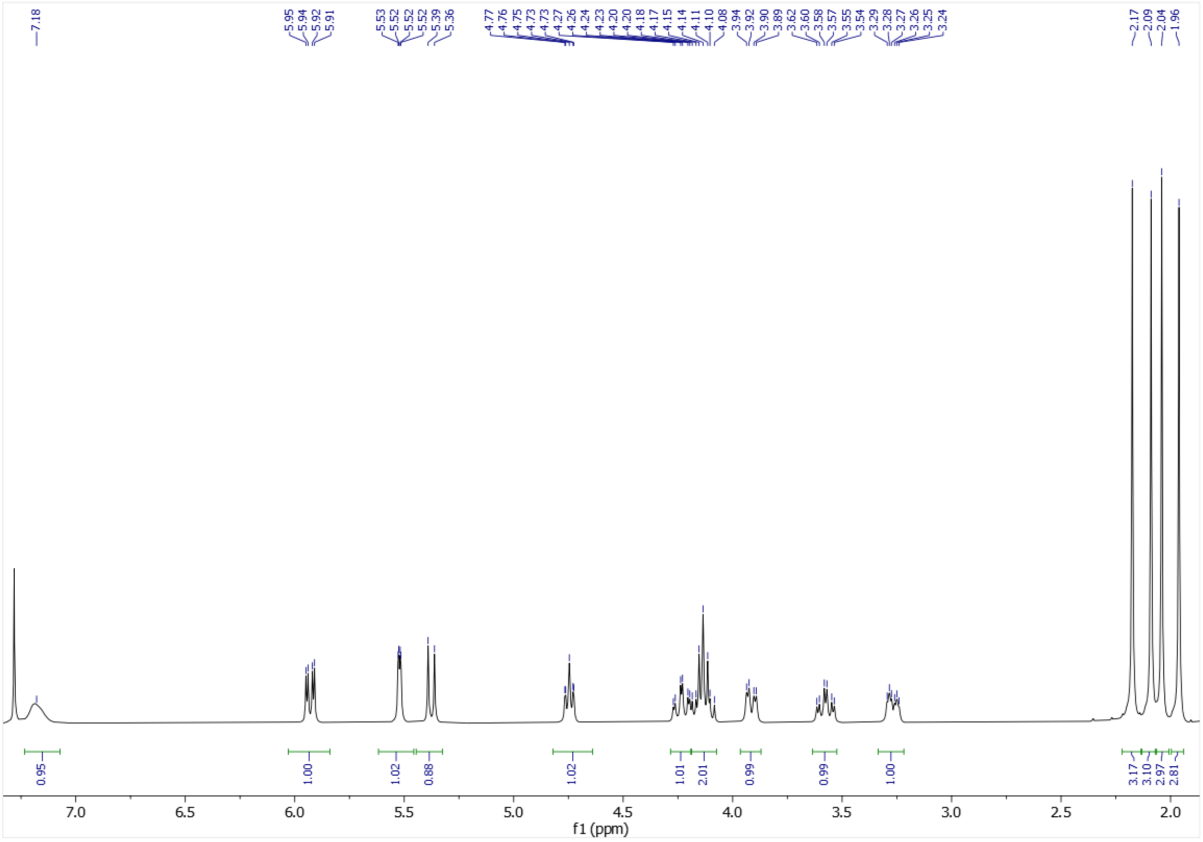

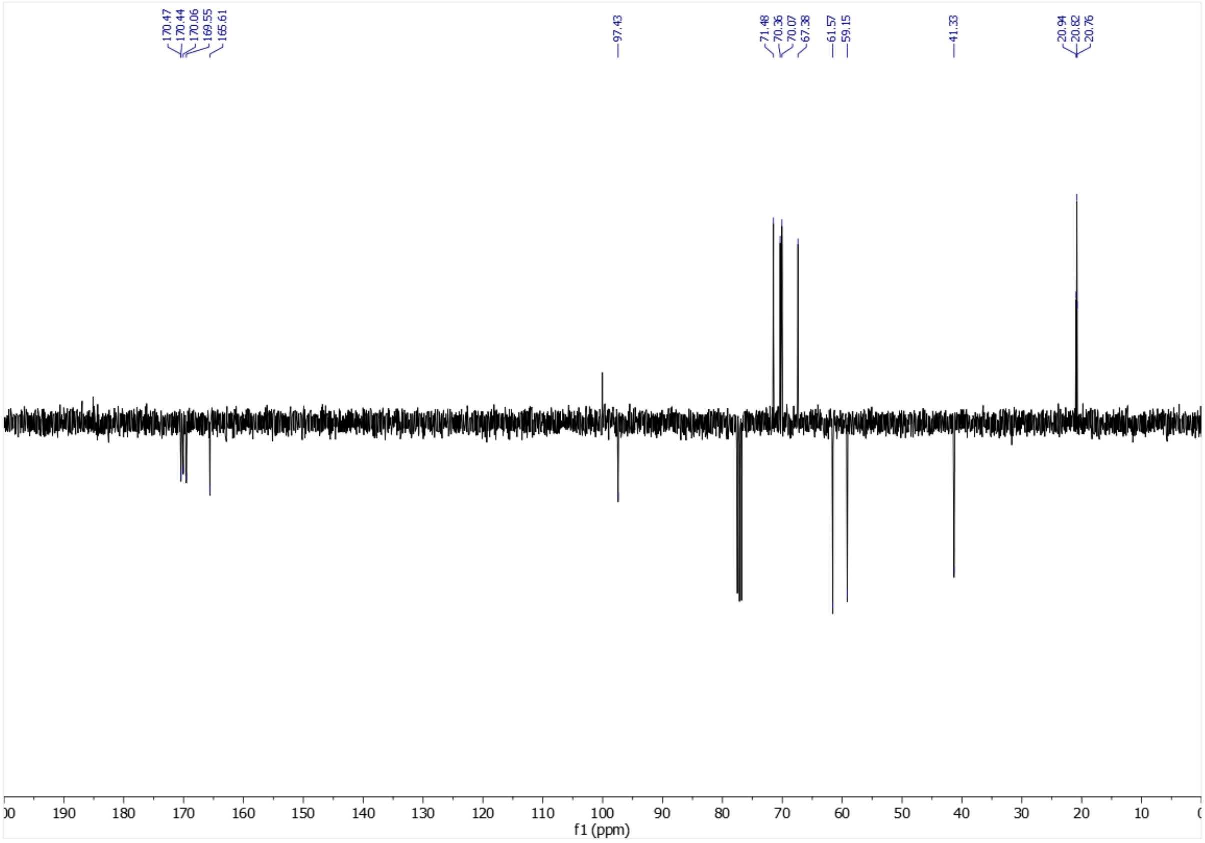

170.47
170.44
170.06
169.55
165.61
97.43
71.48
70.36
70.07
67.38
61.57
59.15
41.33
20.94
20.82
20.76
190 180 170 160 150 140 130 120 110 100 90 80 70 60 50 40 30 20 10
f1 (ppm)

^{1}H (400 MHz) and ^{13}C J-MOD (90 MHz) NMR spectra of compound **17** in $CDCl_3$

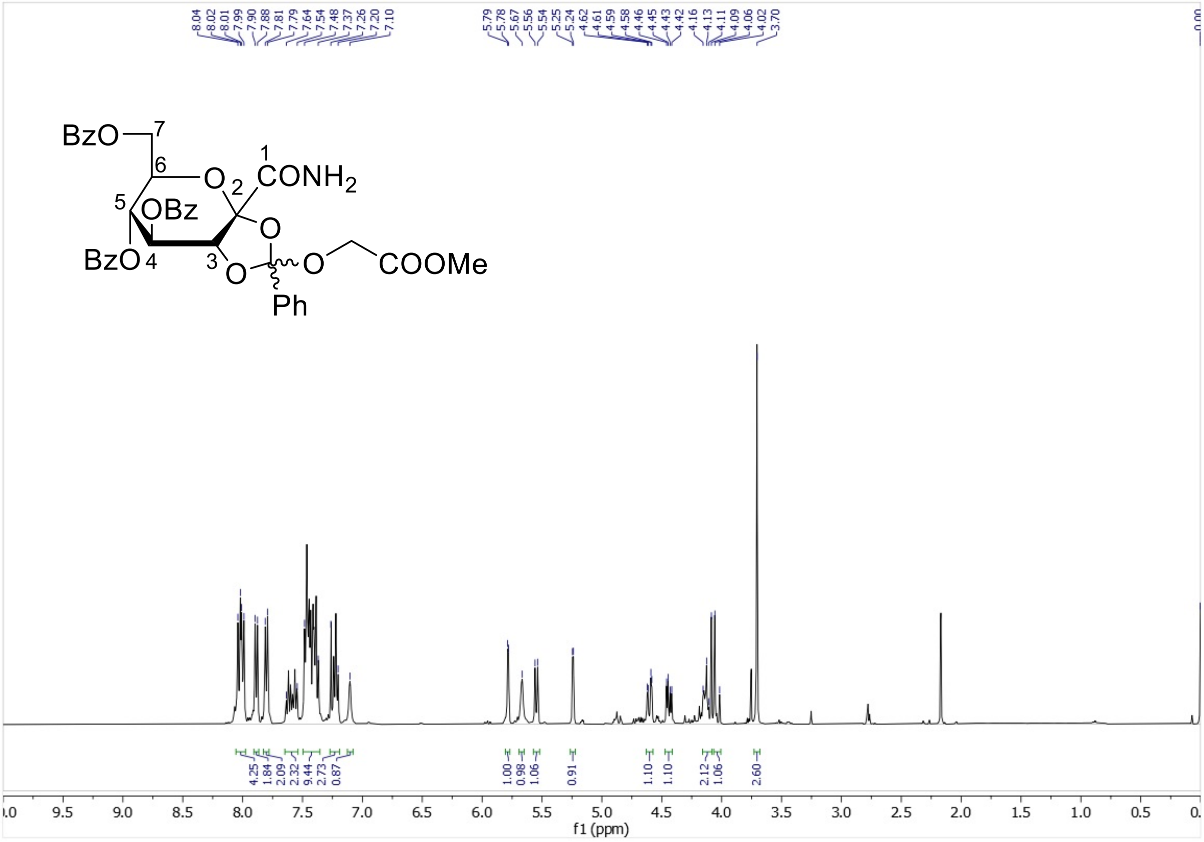

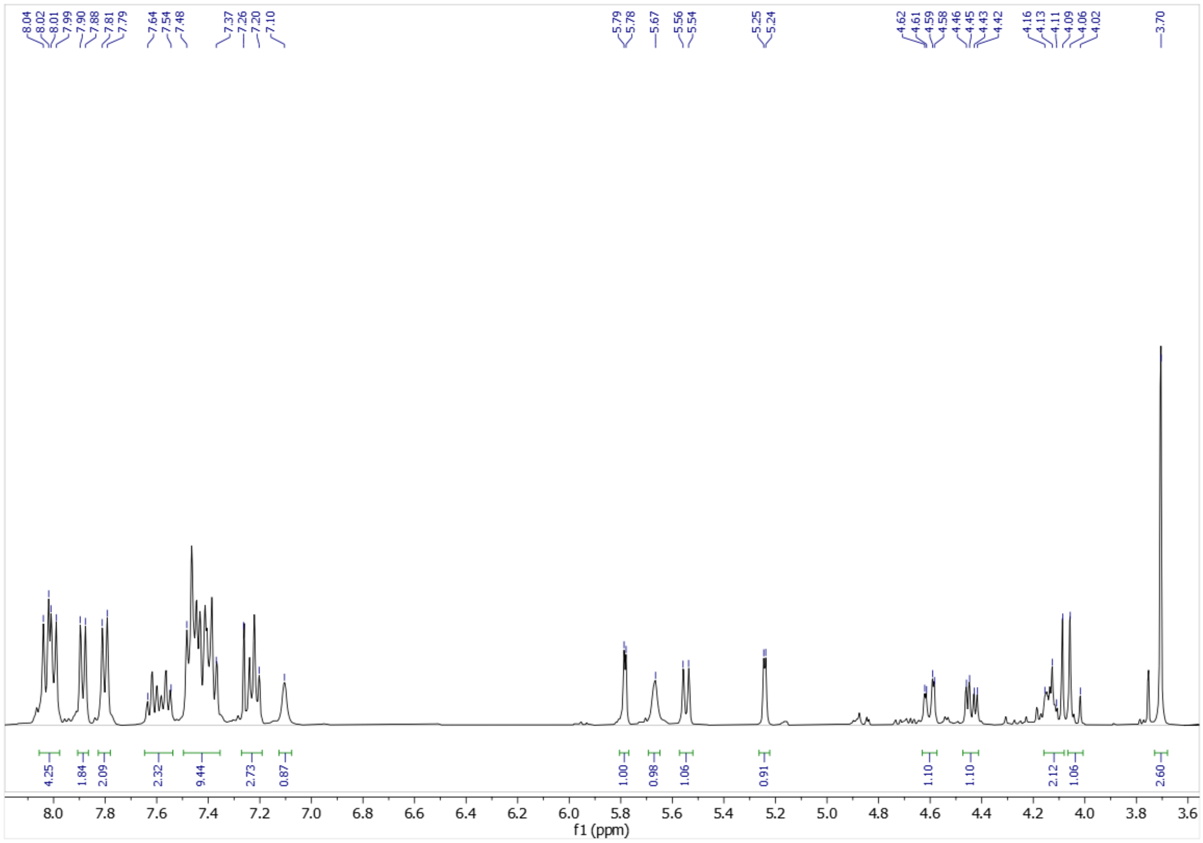

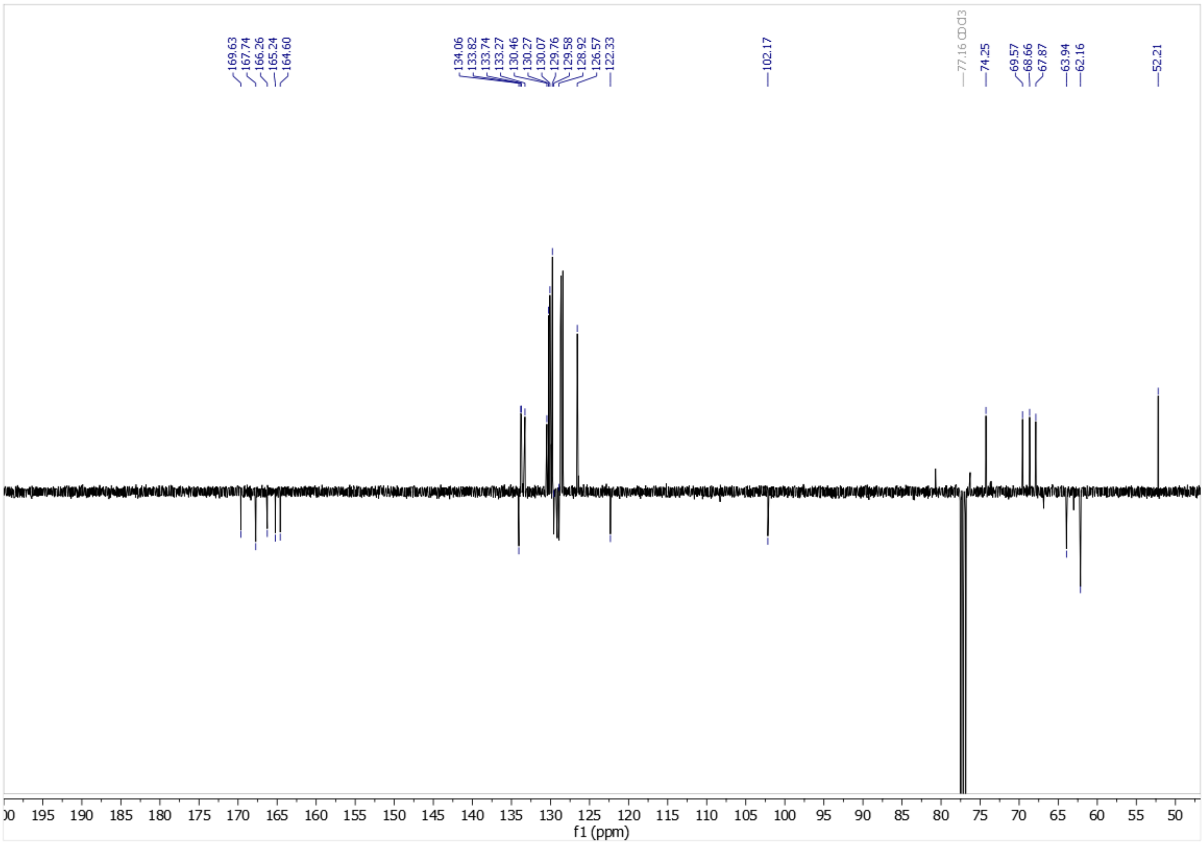
169.63
167.74
166.26
165.24
164.60
134.06
133.82
133.74
133.27
130.46
130.27
130.07
129.76
129.58
128.92
126.57
122.33
102.17
77.16 CDCl3
74.25
69.57
68.66
67.87
63.94
62.16
52.21
f1 (ppm)

^{1}H (360 MHz) and ^{13}C J-MOD (90 MHz) NMR spectra of compound **19** in $CDCl_3$

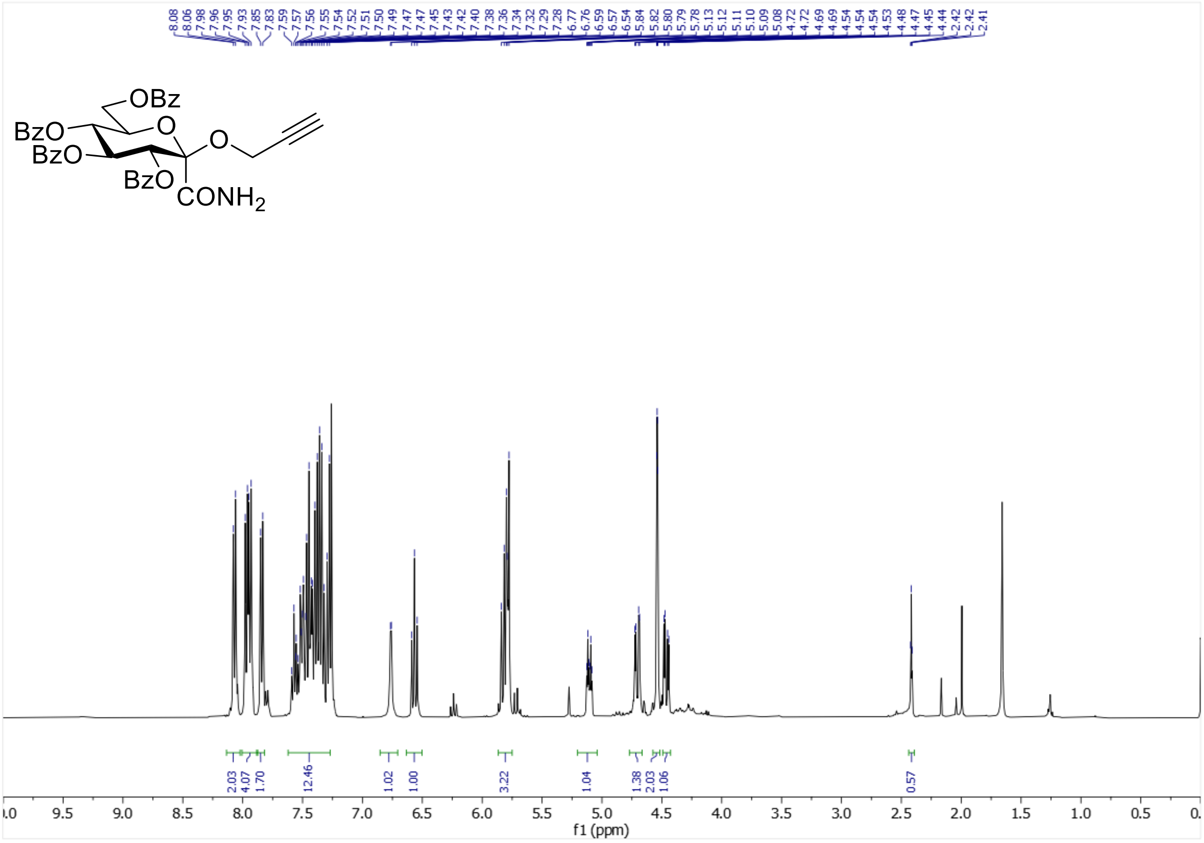

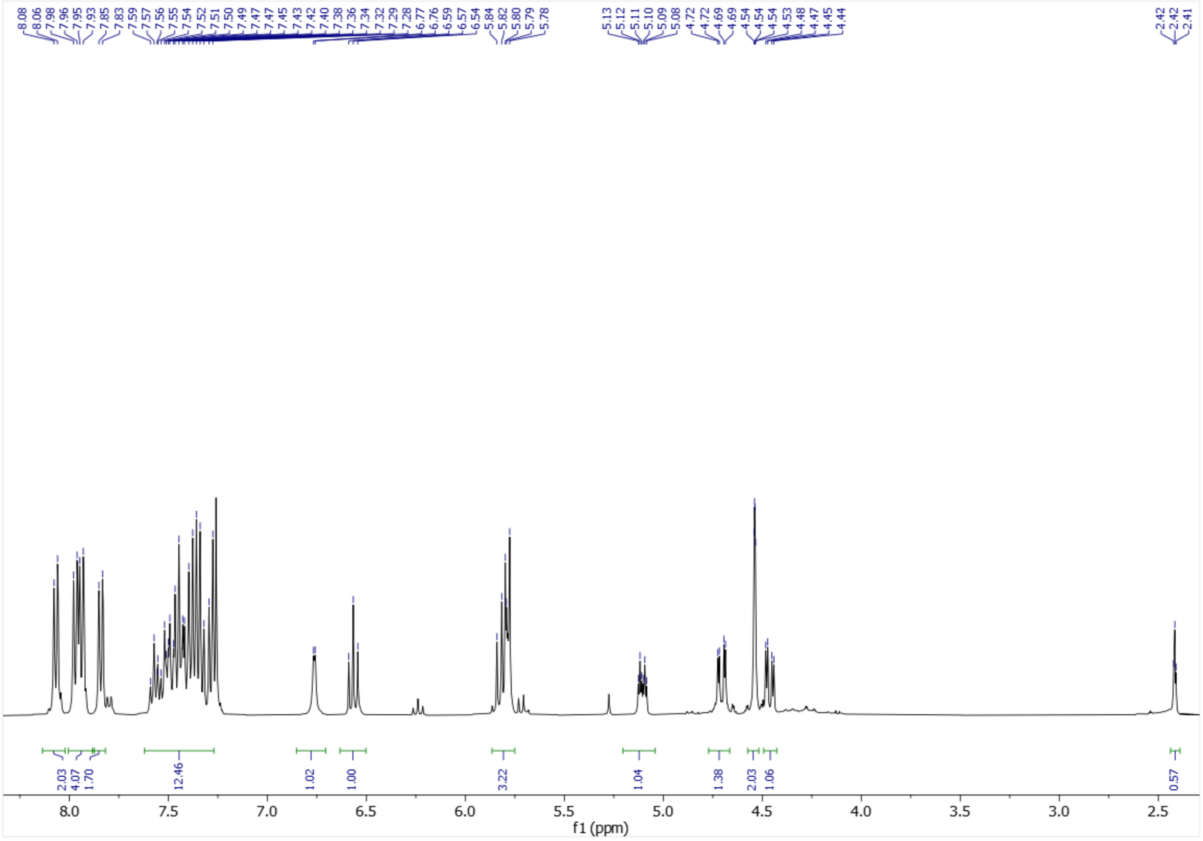

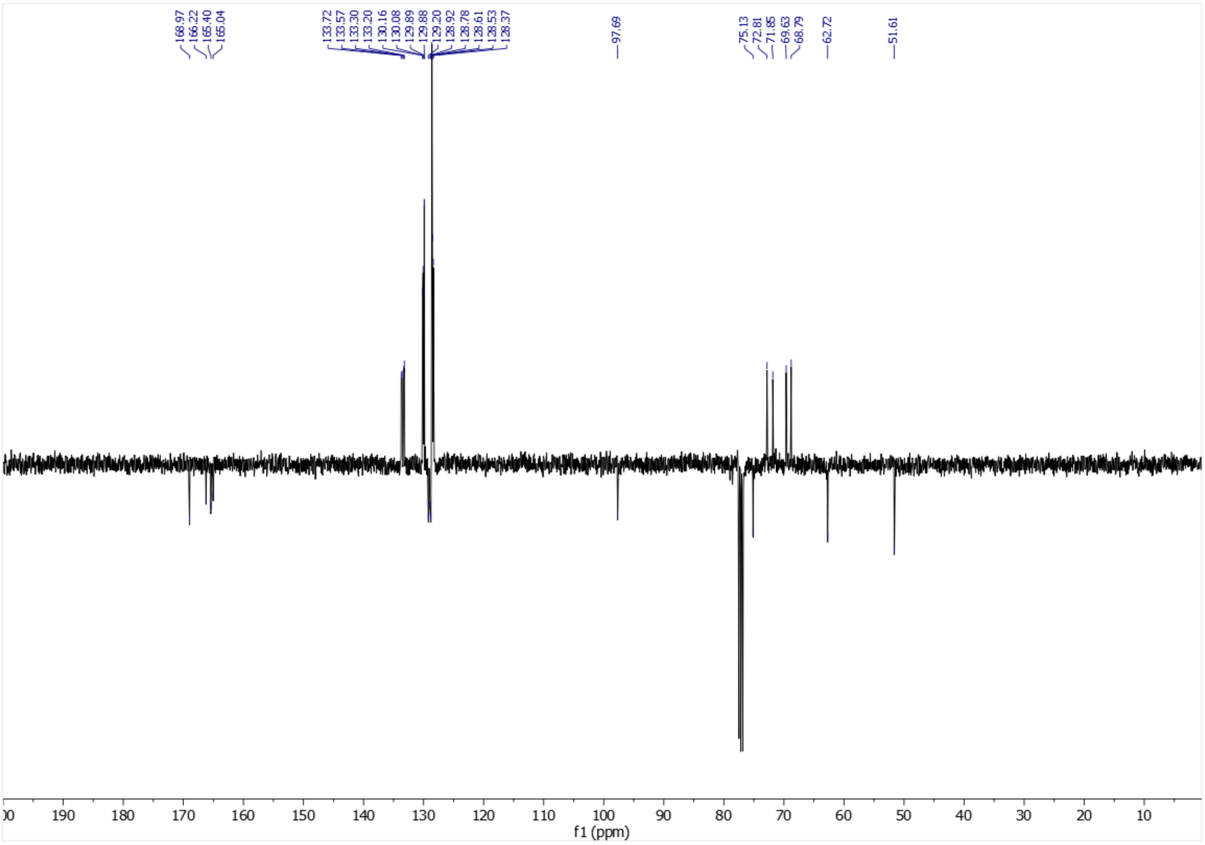
168.97
166.22
165.40
165.04
133.72
133.57
133.30
133.20
130.16
130.08
129.89
129.88
129.20
128.92
128.78
128.61
128.53
128.37
97.69
75.13
72.81
71.85
69.63
68.79
62.72
51.61
f1 (ppm)

^{1}H (360 MHz) and ^{13}C J-MOD (90 MHz) NMR spectra of compound **20** in $CDCl_3$

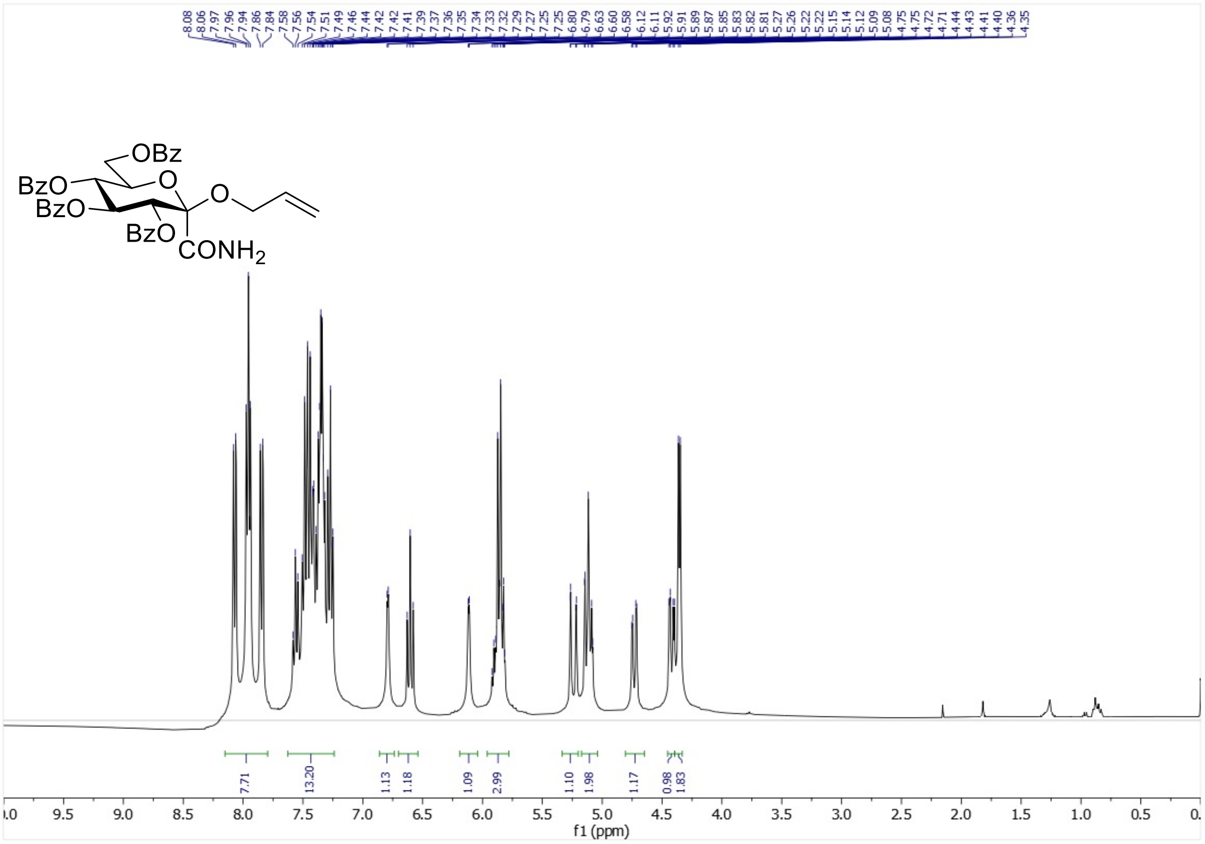

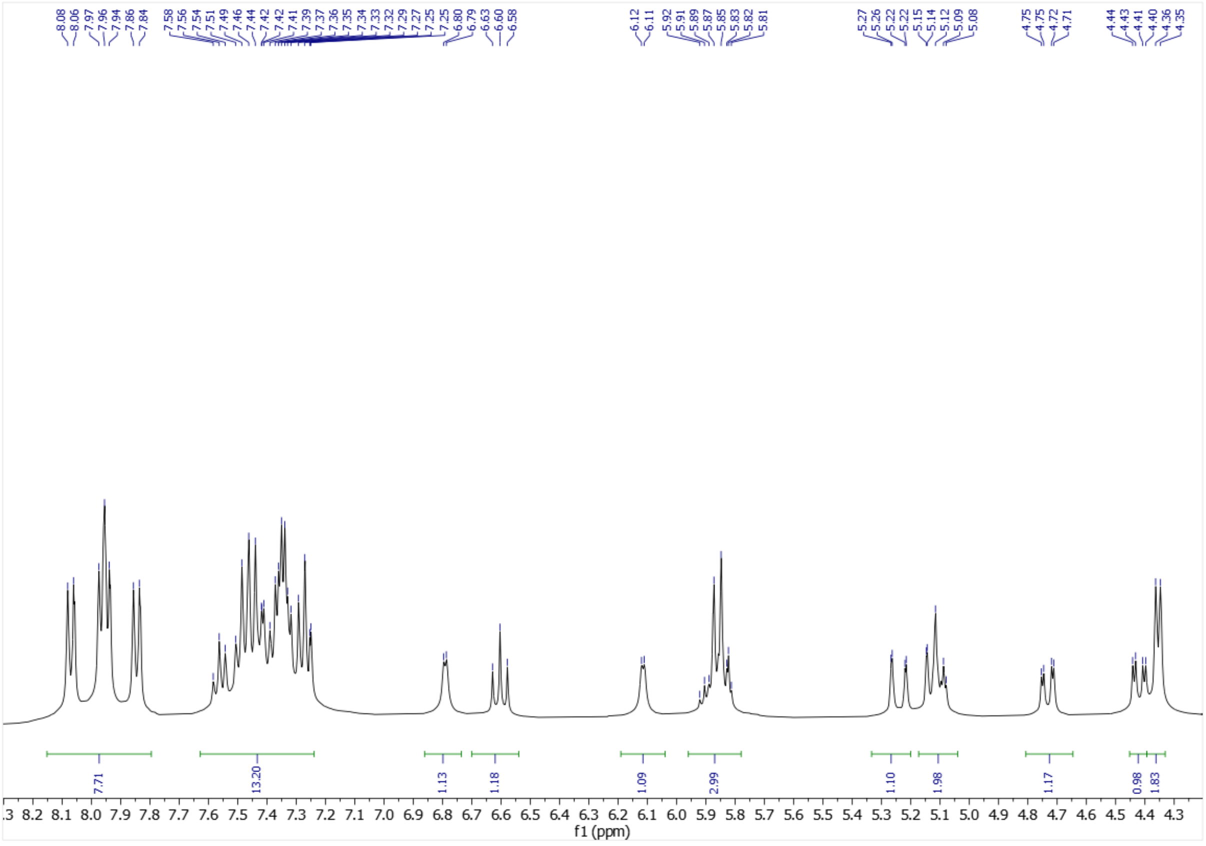

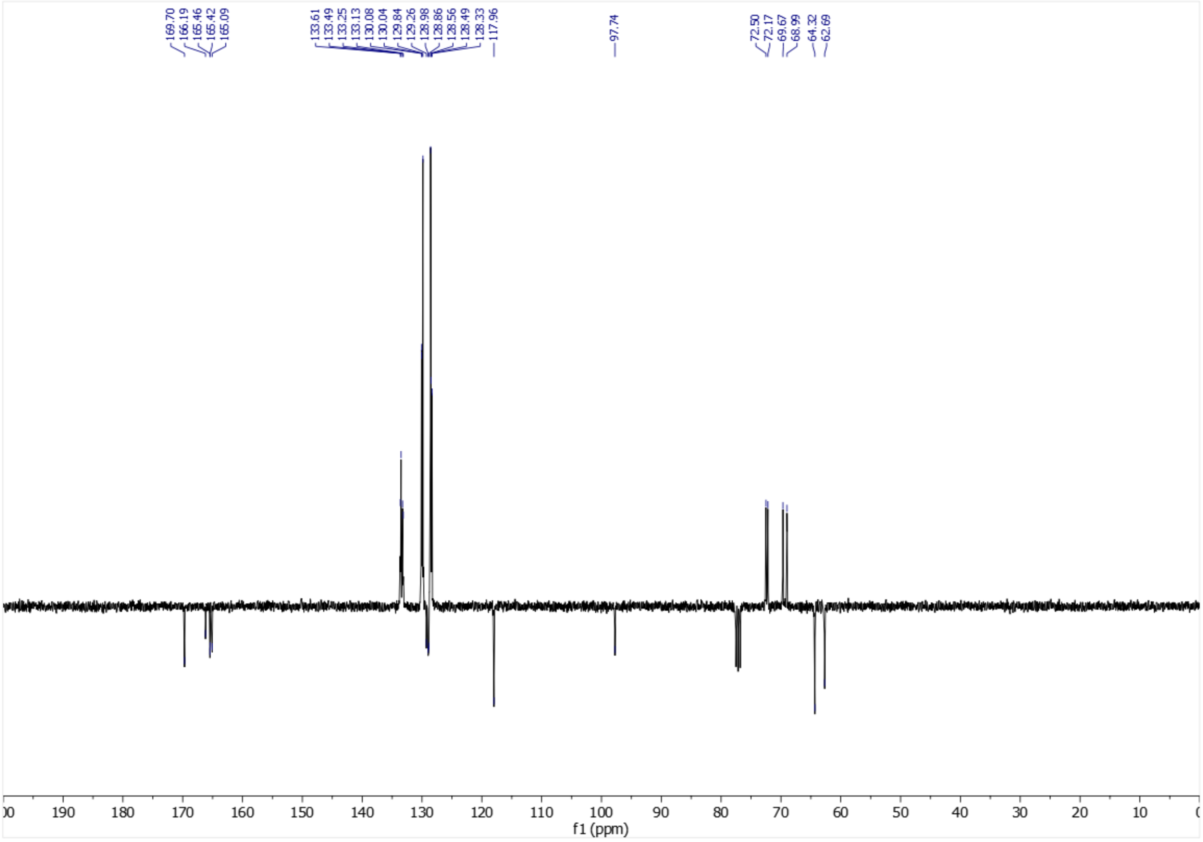

169.70
166.19
165.46
165.42
165.09
133.61
133.49
133.25
133.13
130.08
130.04
129.84
129.26
128.98
128.86
128.56
128.49
128.33
117.96
97.74
72.50
72.17
69.67
68.99
64.32
62.69
f1 (ppm)

^{1}H (400 MHz) and ^{13}C J-MOD (100 MHz) NMR spectra of compound **21** in $CDCl_3$

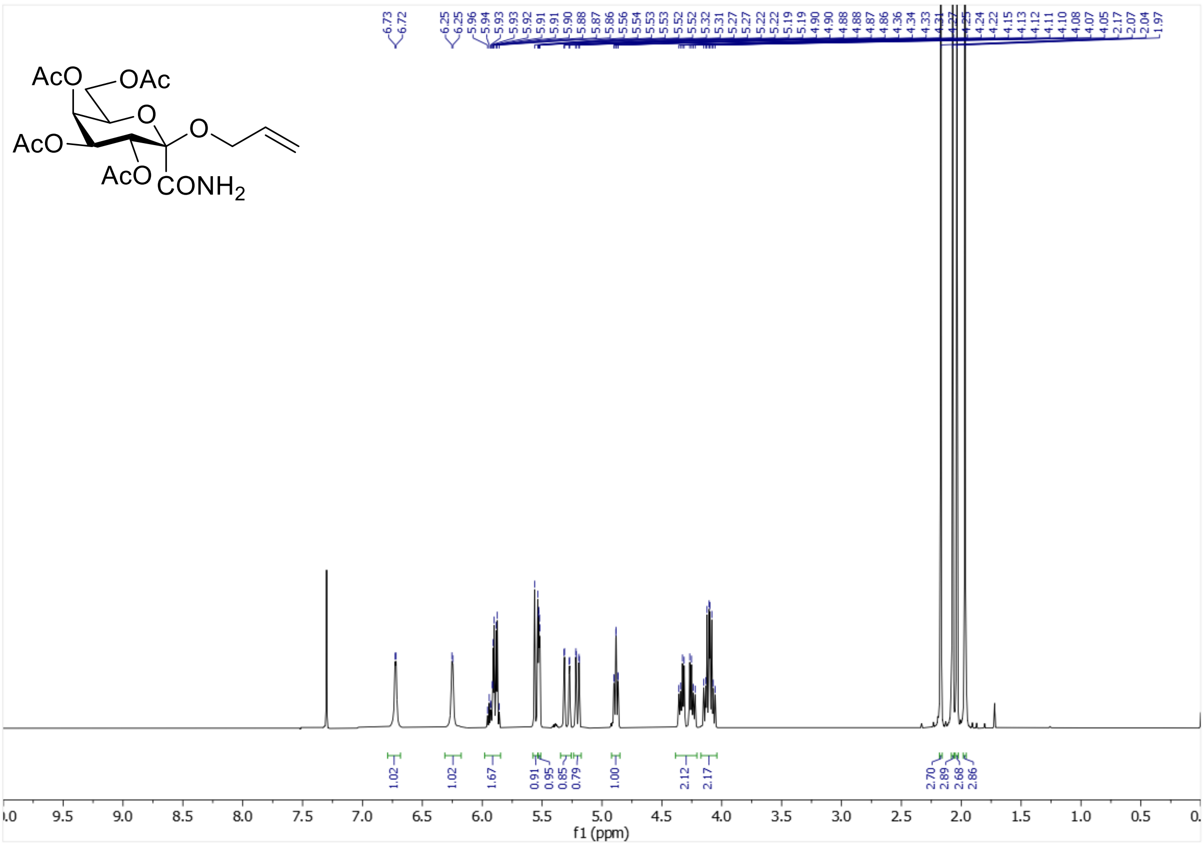

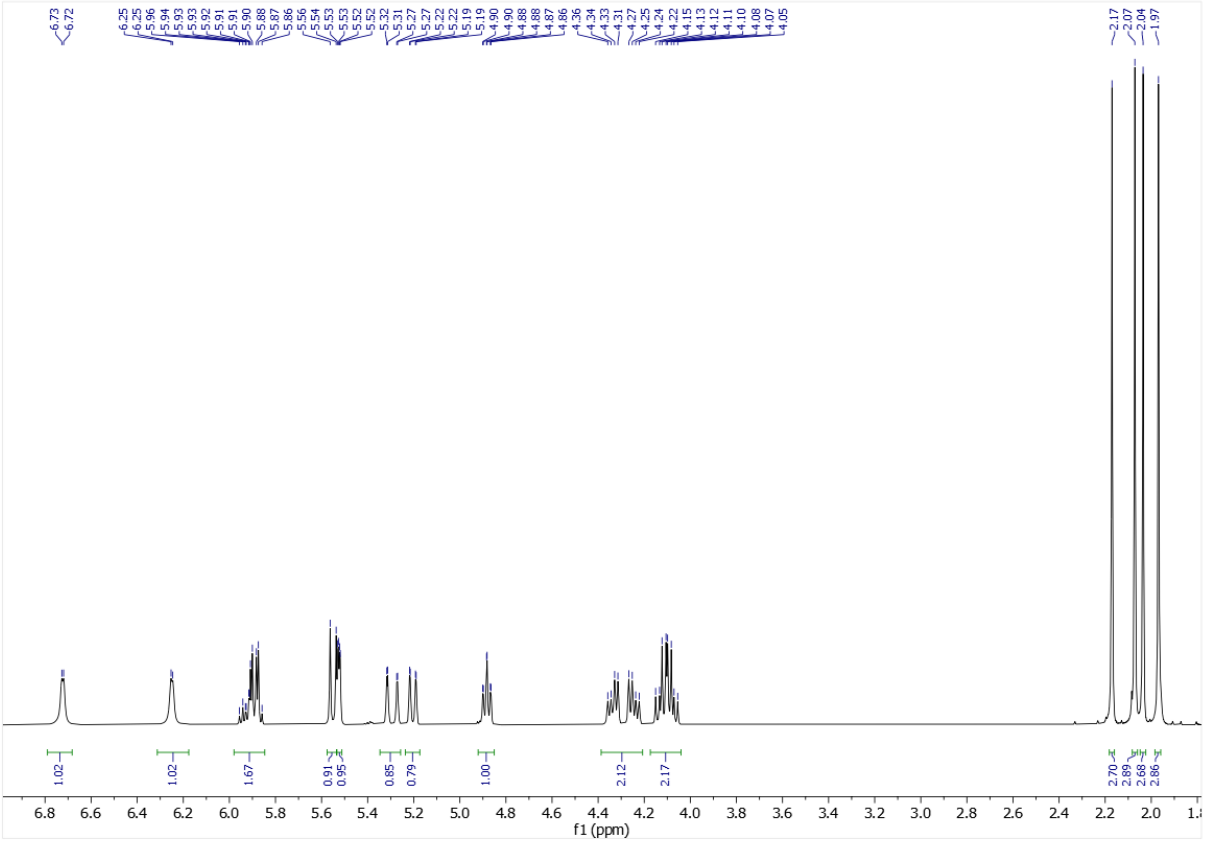

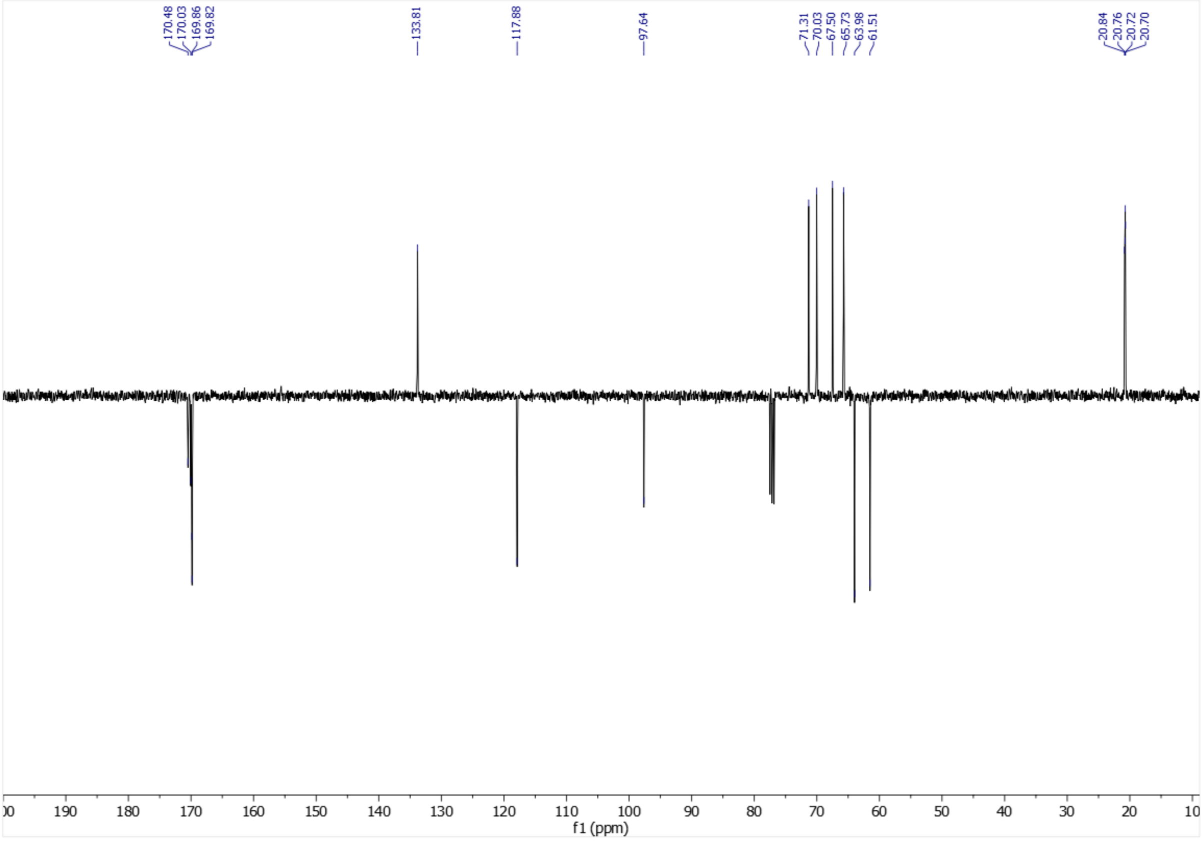
170.48
170.03
169.86
169.82
133.81
117.88
97.64
71.31
70.03
67.50
65.73
63.98
61.51
20.84
20.76
20.72
20.70
f1 (ppm)

^{1}H (400 MHz) and ^{13}C J-MOD (100 MHz) NMR spectra of compound **22** in $CDCl_3$

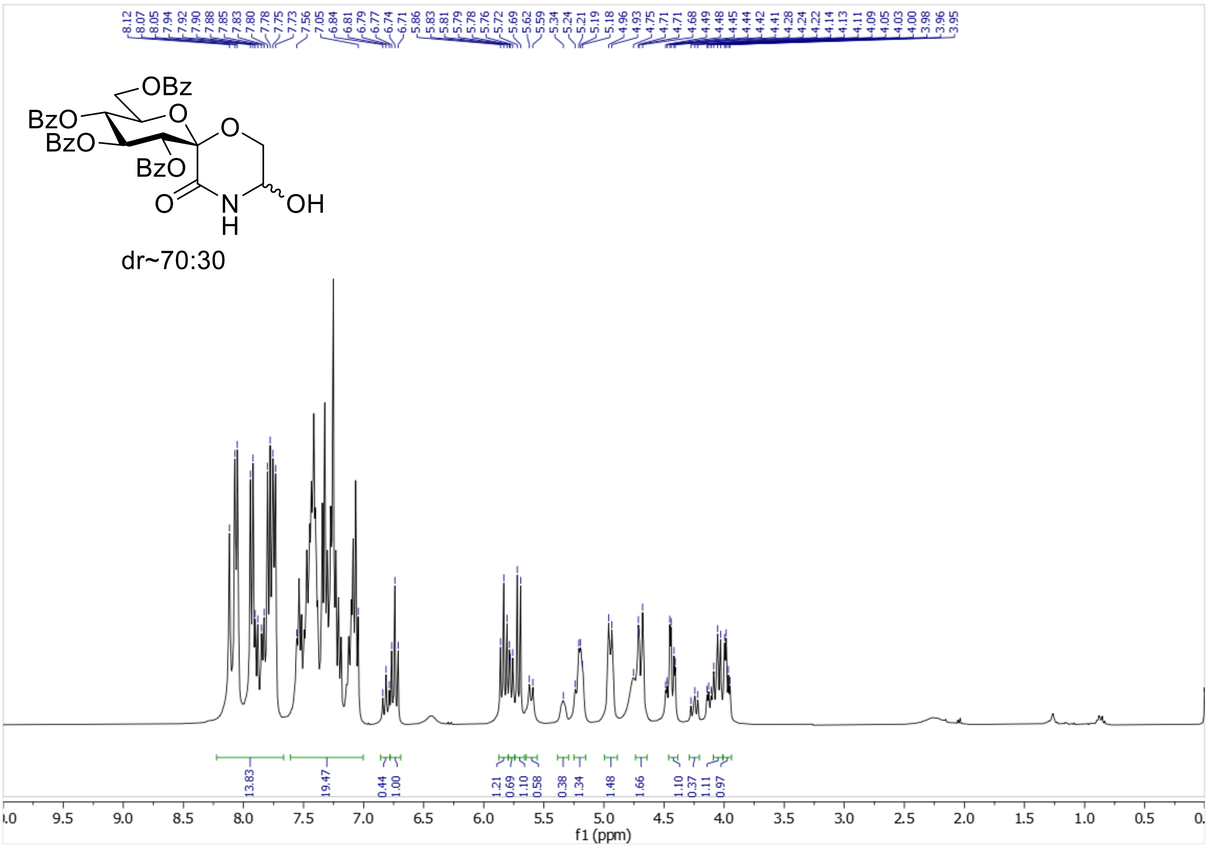

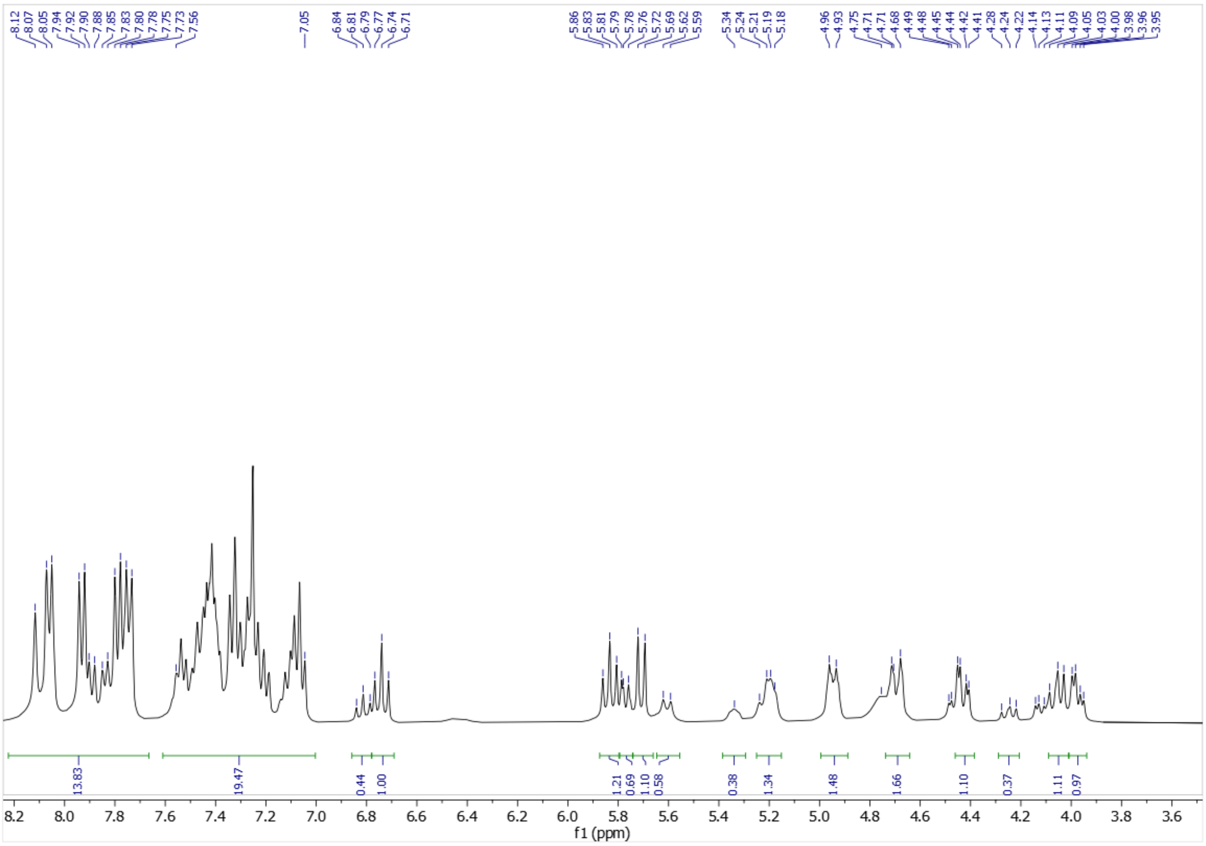

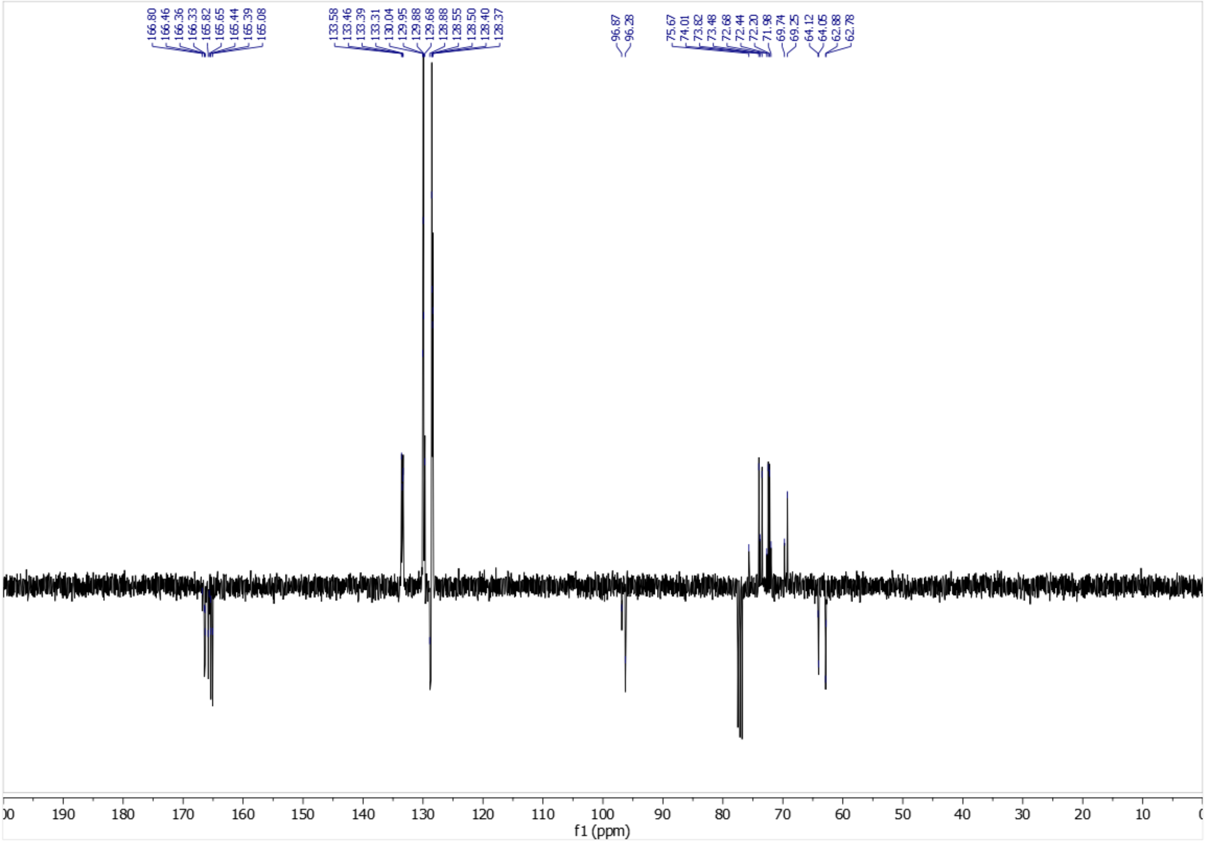
166.80
166.46
166.36
166.33
165.82
165.65
165.44
165.39
165.08
133.58
133.46
133.39
133.31
130.04
129.95
129.88
129.68
128.88
128.55
128.50
128.40
128.37
96.87
96.28
75.67
74.01
73.82
73.48
72.68
72.44
72.20
71.98
69.74
69.25
64.12
64.05
62.88
62.78
f1 (ppm)

^{1}H (400 MHz) and ^{13}C J-MOD (100 MHz) NMR spectra of compound **23** in $CDCl_3$

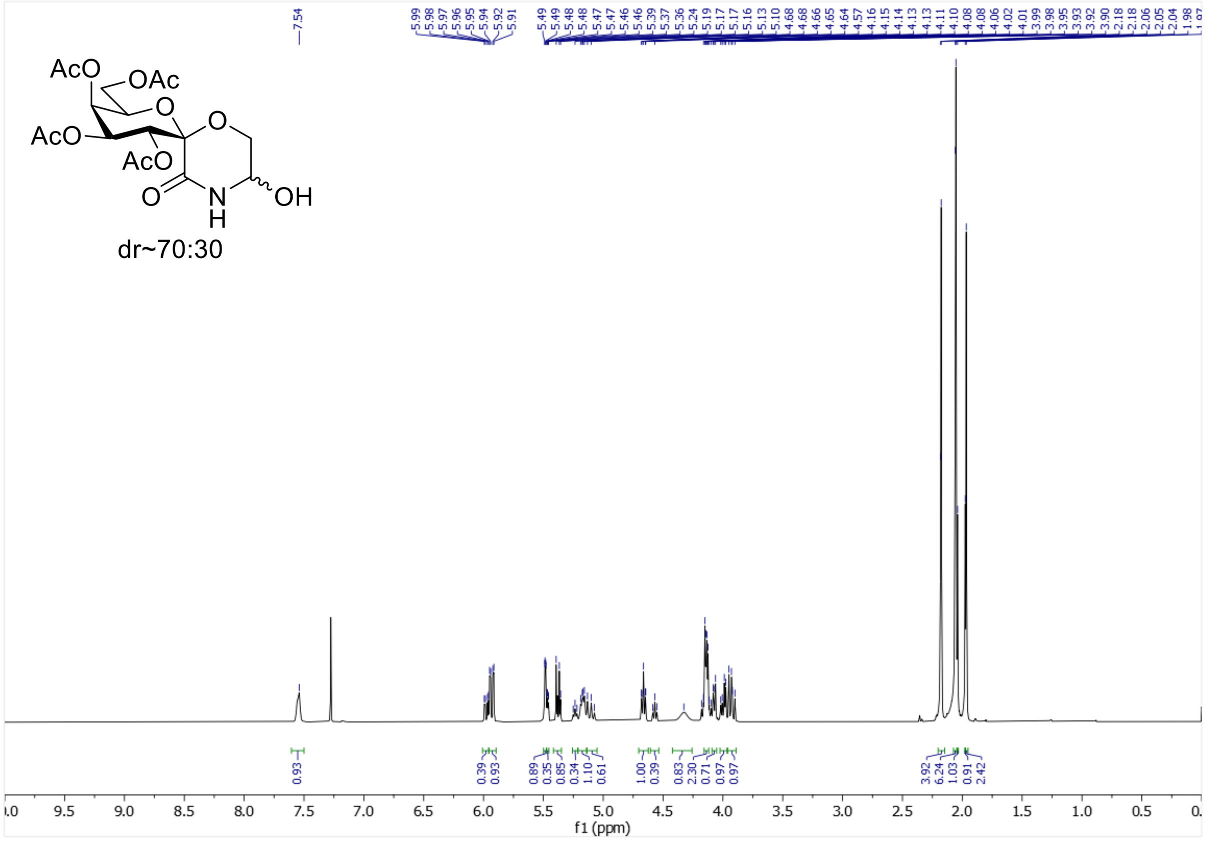

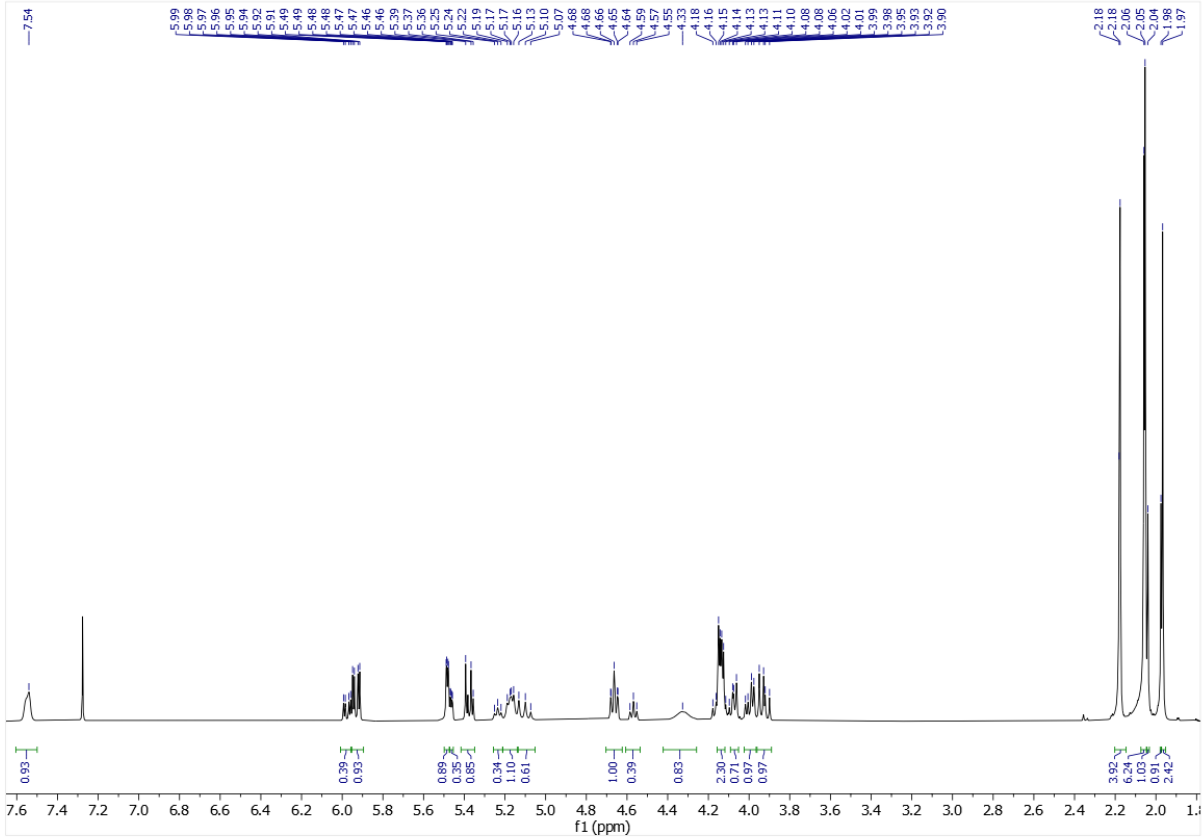

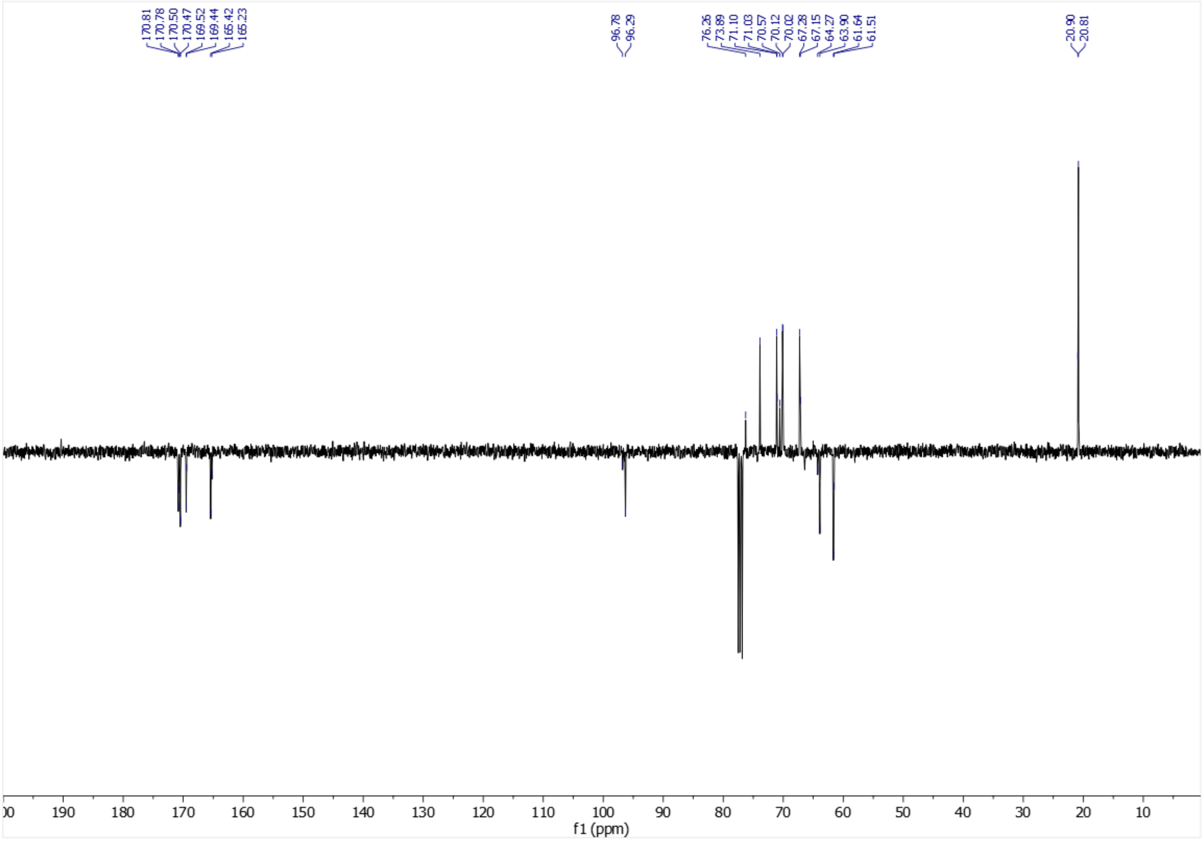
170.81
170.78
170.50
170.47
169.52
169.44
165.42
165.23
96.78
96.29
76.26
73.89
71.10
71.03
70.57
70.12
70.02
67.28
67.15
64.27
63.90
61.64
61.51
20.90
20.81
f1 (ppm)

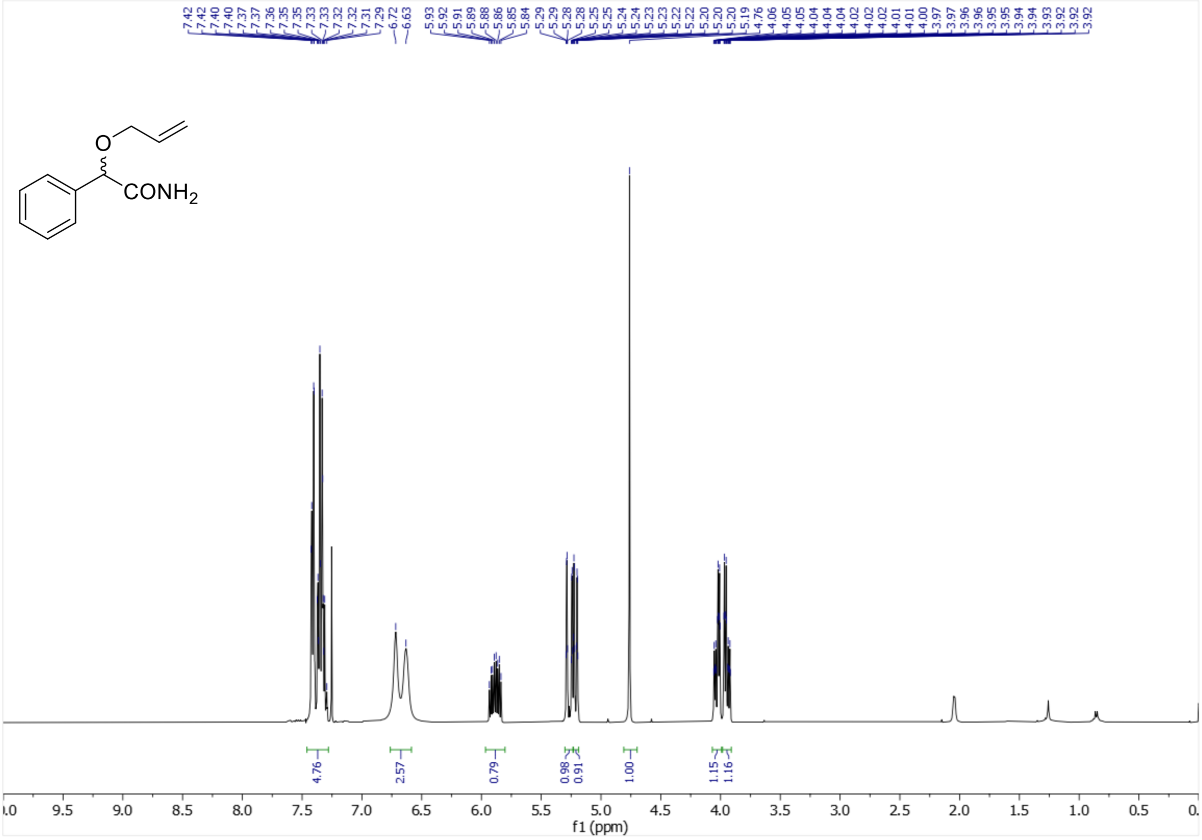
O
CONH2
f1 (ppm)

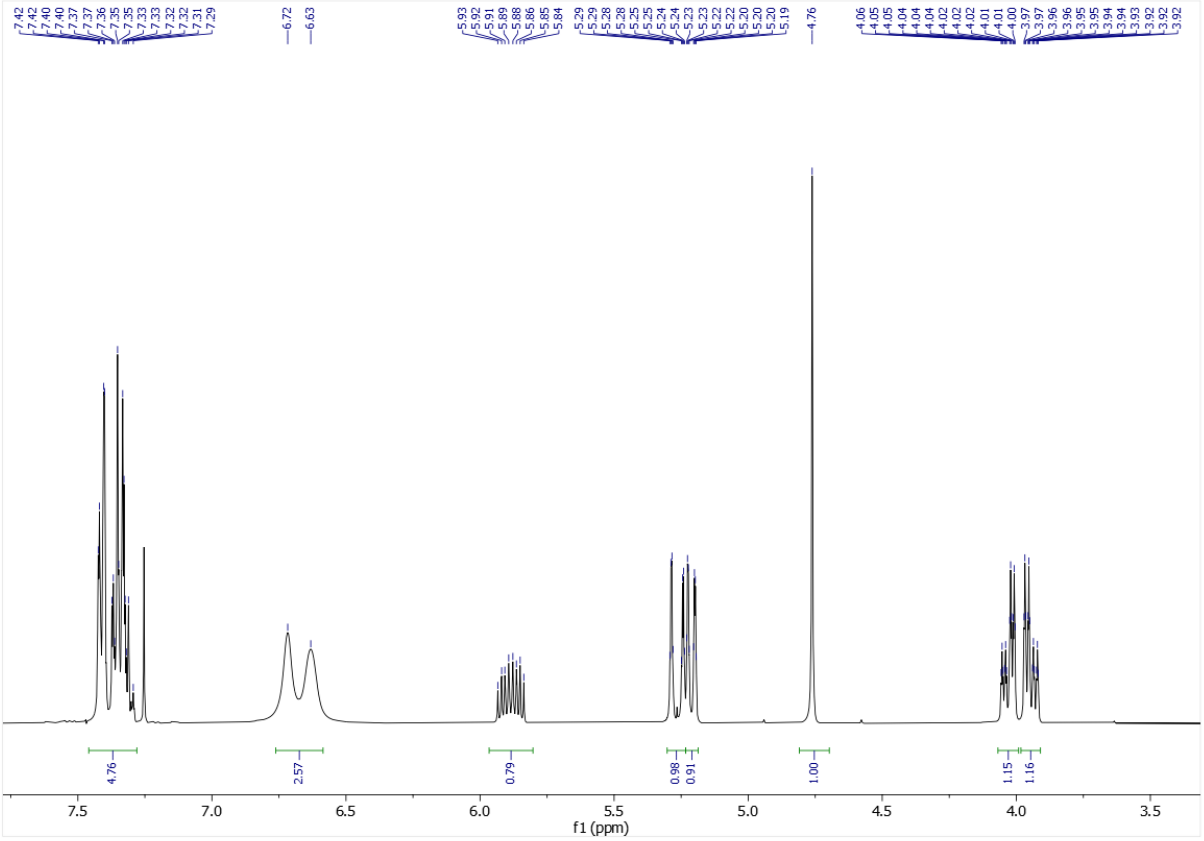
f1 (ppm)

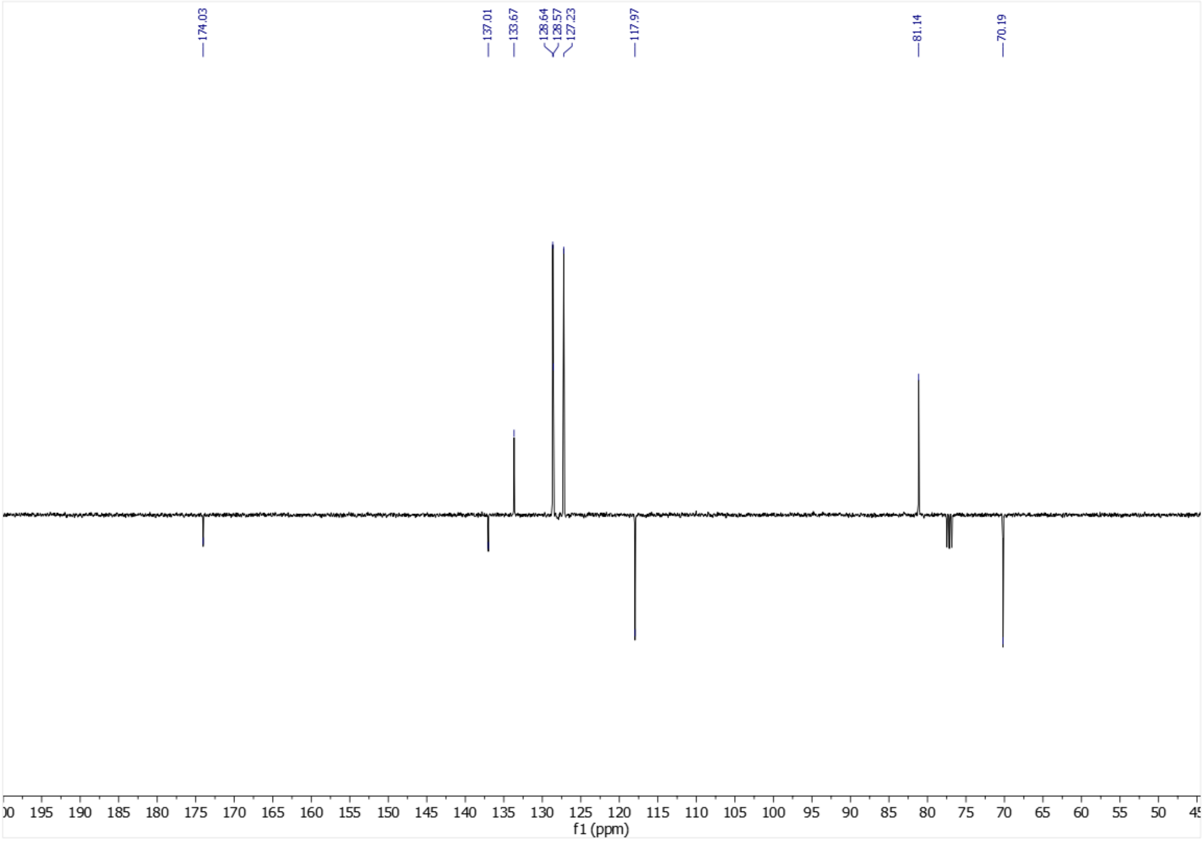

^{1}H (400 MHz) and ^{13}C J-MOD (100 MHz) NMR spectra of compound **24** in $CDCl_3$

^{1}H (400 MHz) and ^{13}C J-MOD (100 MHz) NMR spectra of compound **25** in DMSO-d6

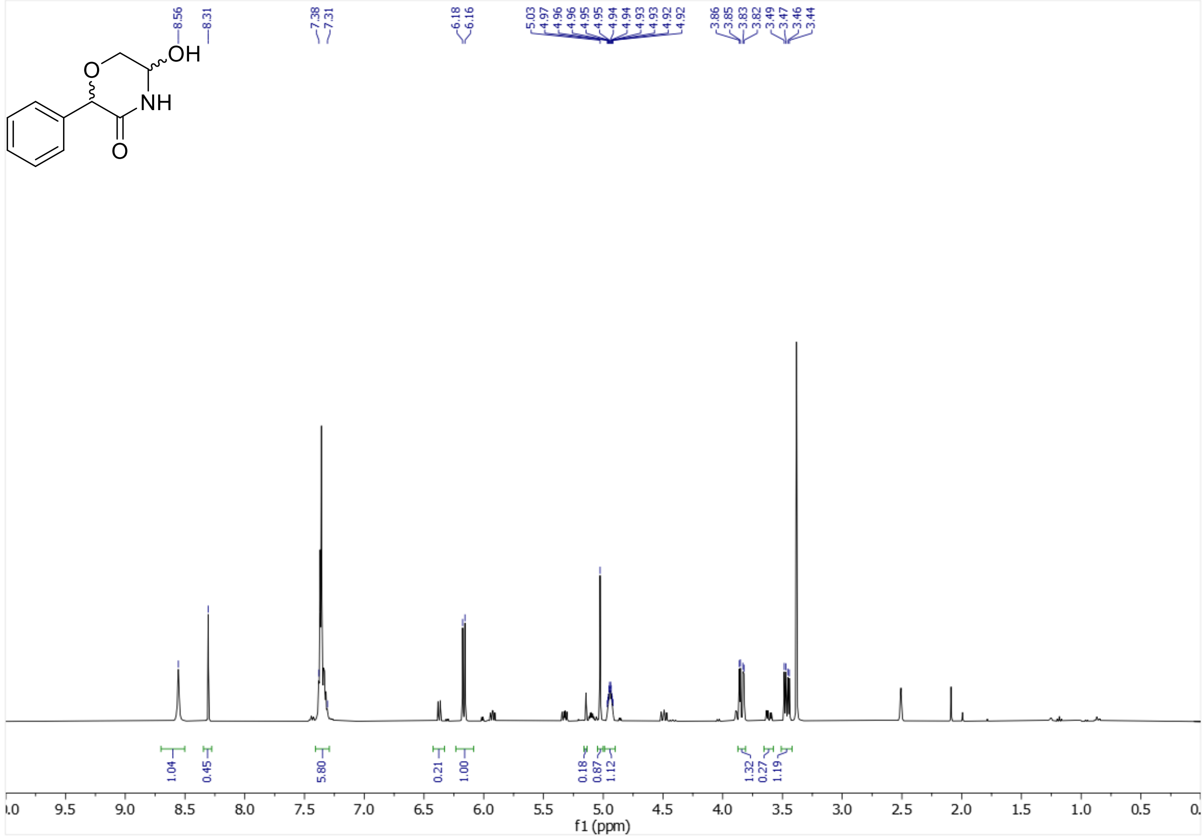

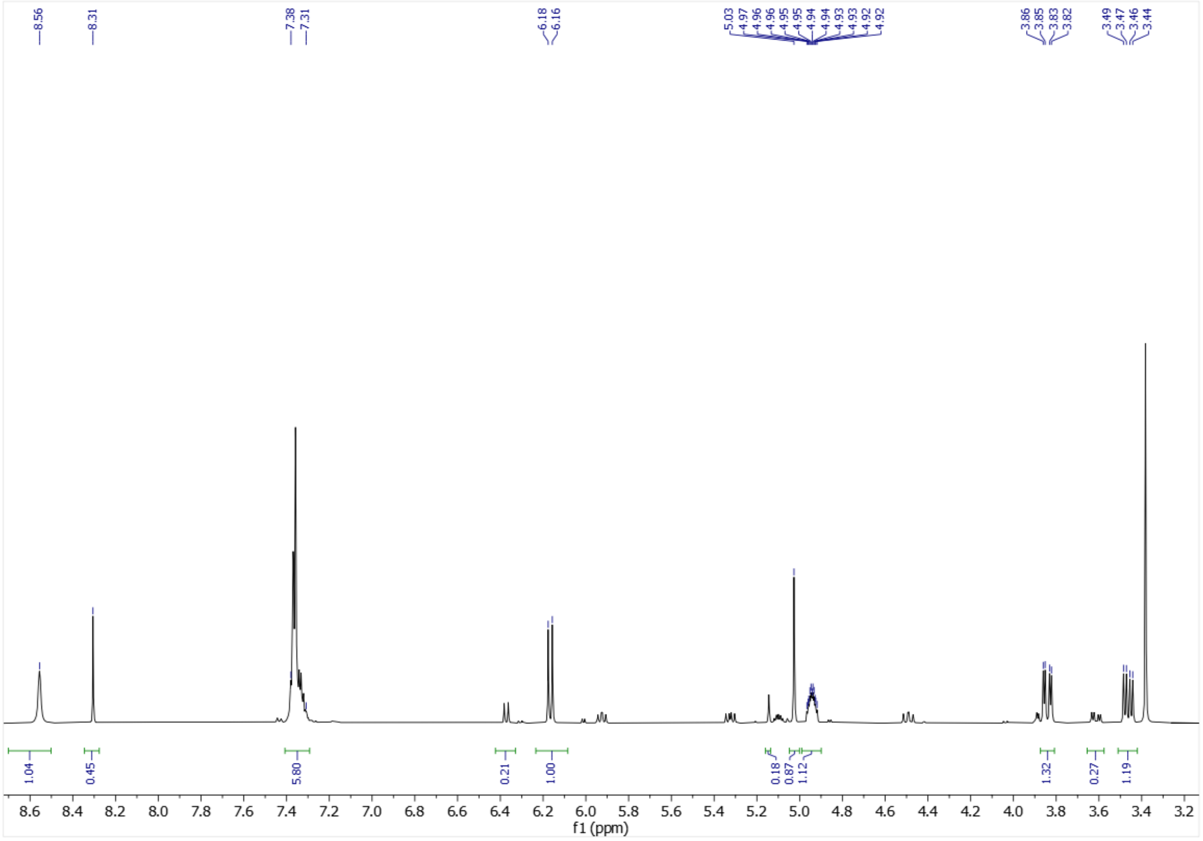

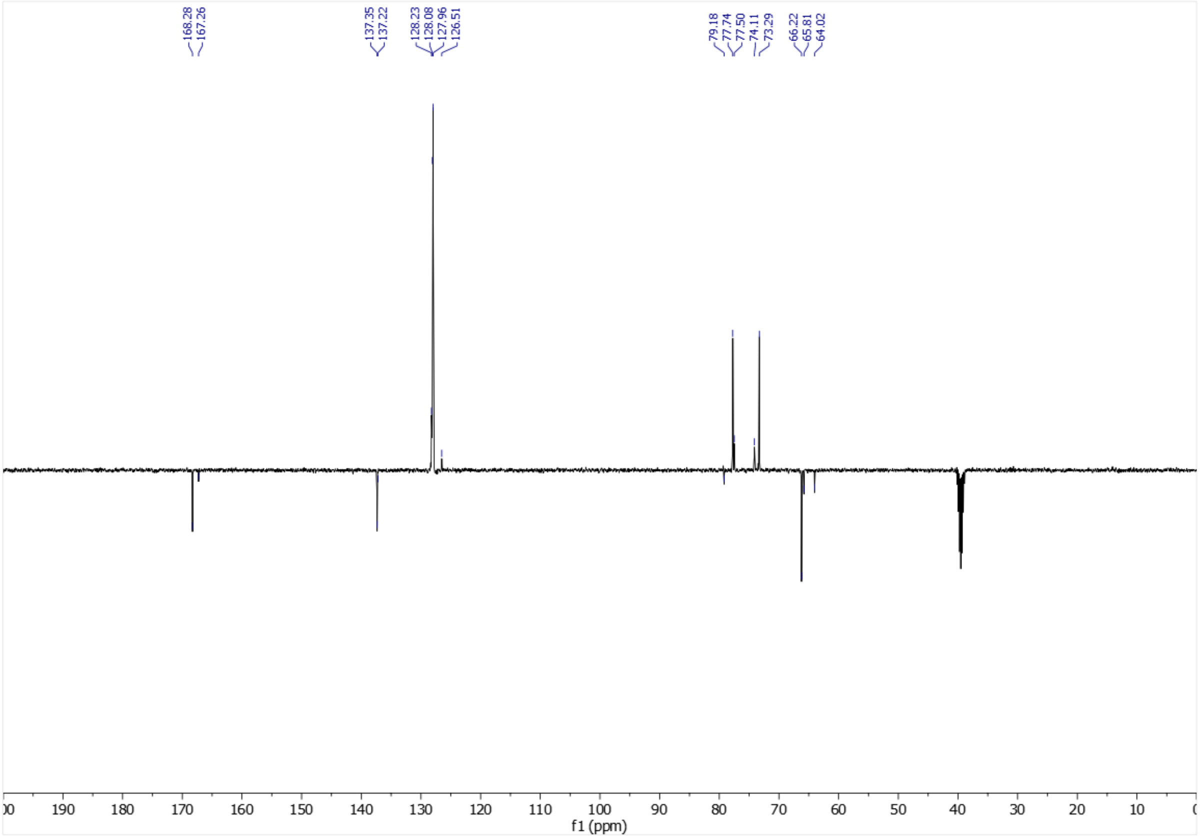

168.28
167.26
137.35
137.22
128.23
128.08
127.96
126.51
79.18
77.74
77.50
74.11
73.29
66.22
65.81
64.02
190 180 170 160 150 140 130 120 110 100 90 80 70 60 50 40 30 20 10
f1 (ppm)

^{1}H (400 MHz) and ^{13}C J-MOD (100 MHz) NMR spectra of compound **26** in $CDCl_3$

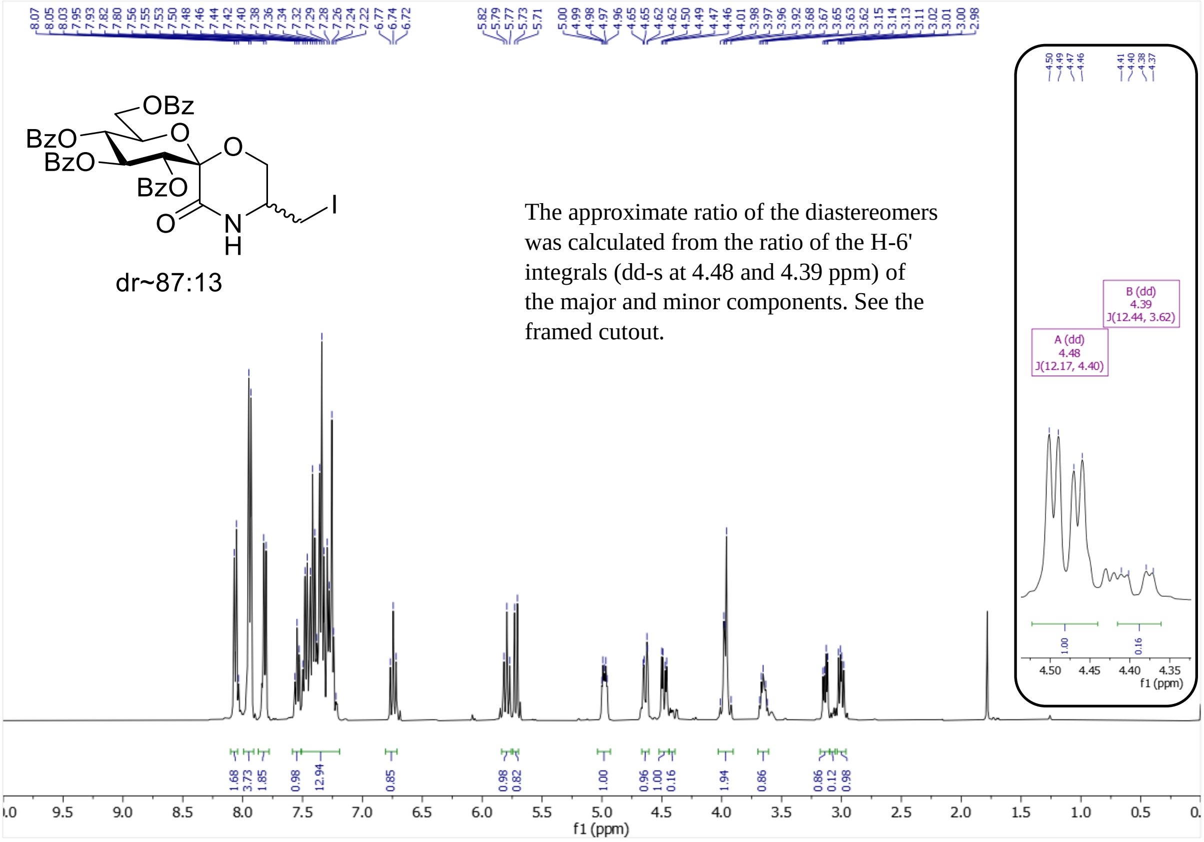

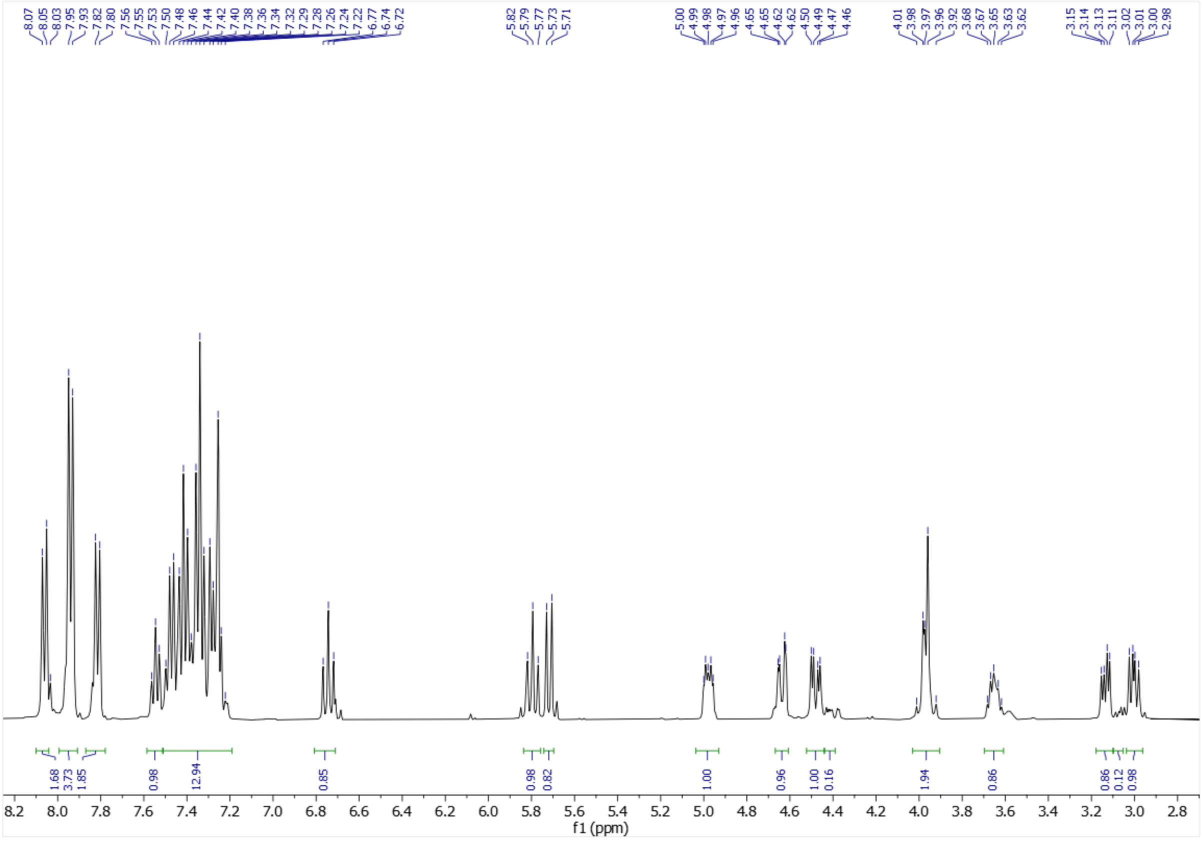

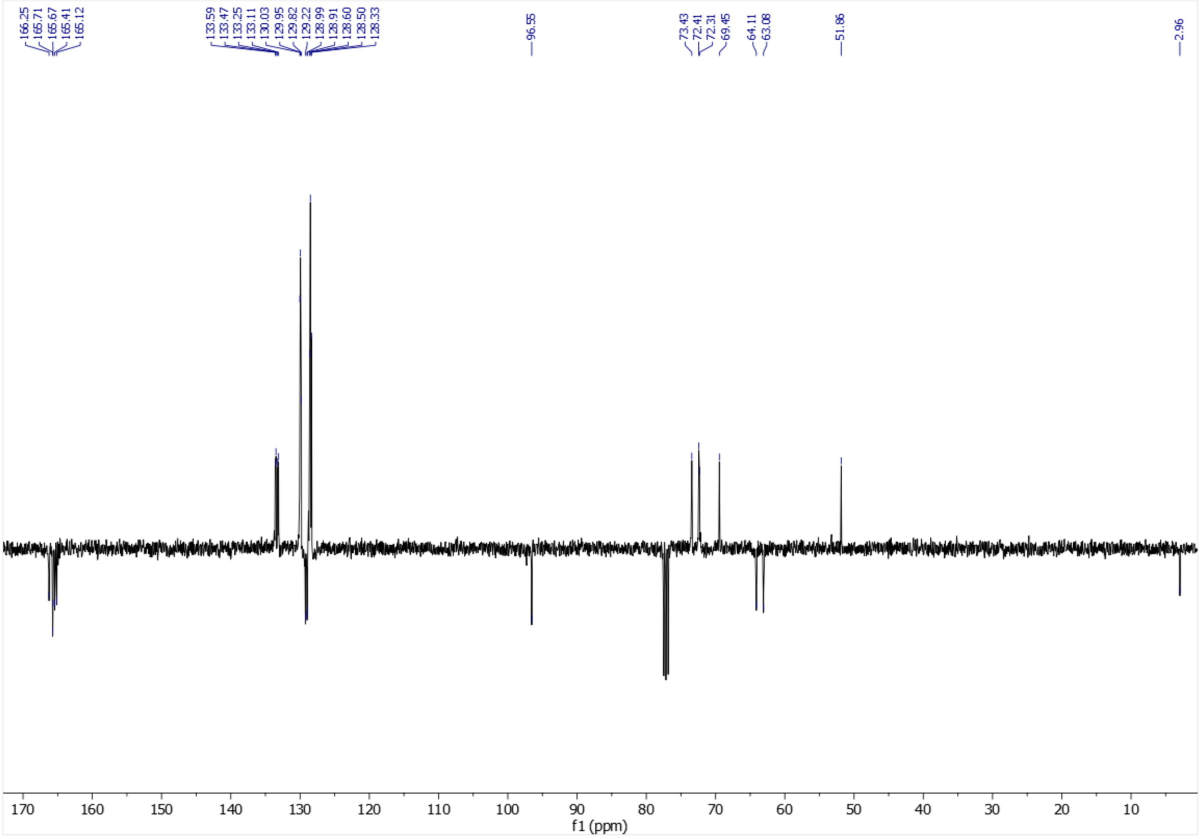
166.25
165.71
165.67
165.41
165.12
133.59
133.47
133.25
133.11
130.03
129.95
129.82
129.22
128.99
128.91
128.60
128.50
128.33
96.55
73.43
72.41
72.31
69.45
64.11
63.08
51.86
2.96
170 160 150 140 130 120 110 100 90 80 70 60 50 40 30 20 10
f1 (ppm)

^{1}H (360 MHz) and ^{13}C J-MOD (90 MHz) NMR spectra of compound **27** in $CDCl_3$

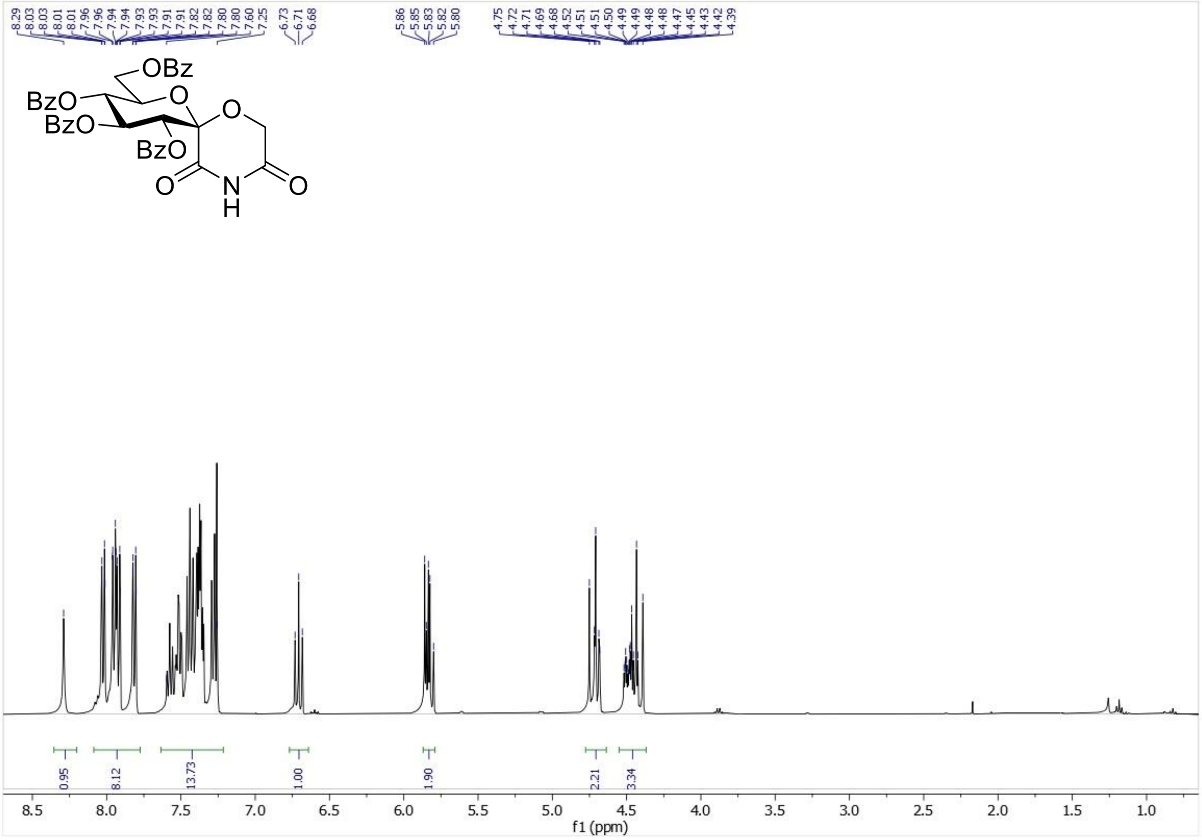

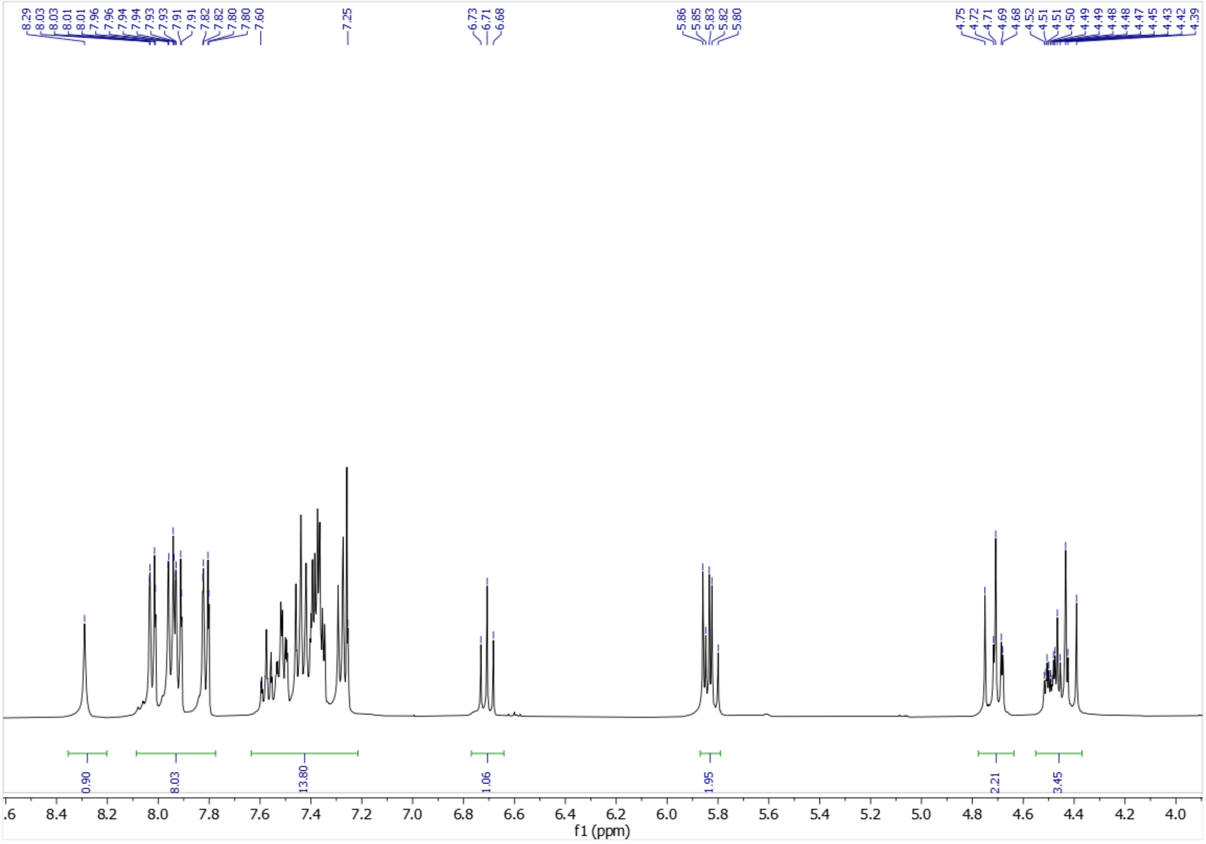

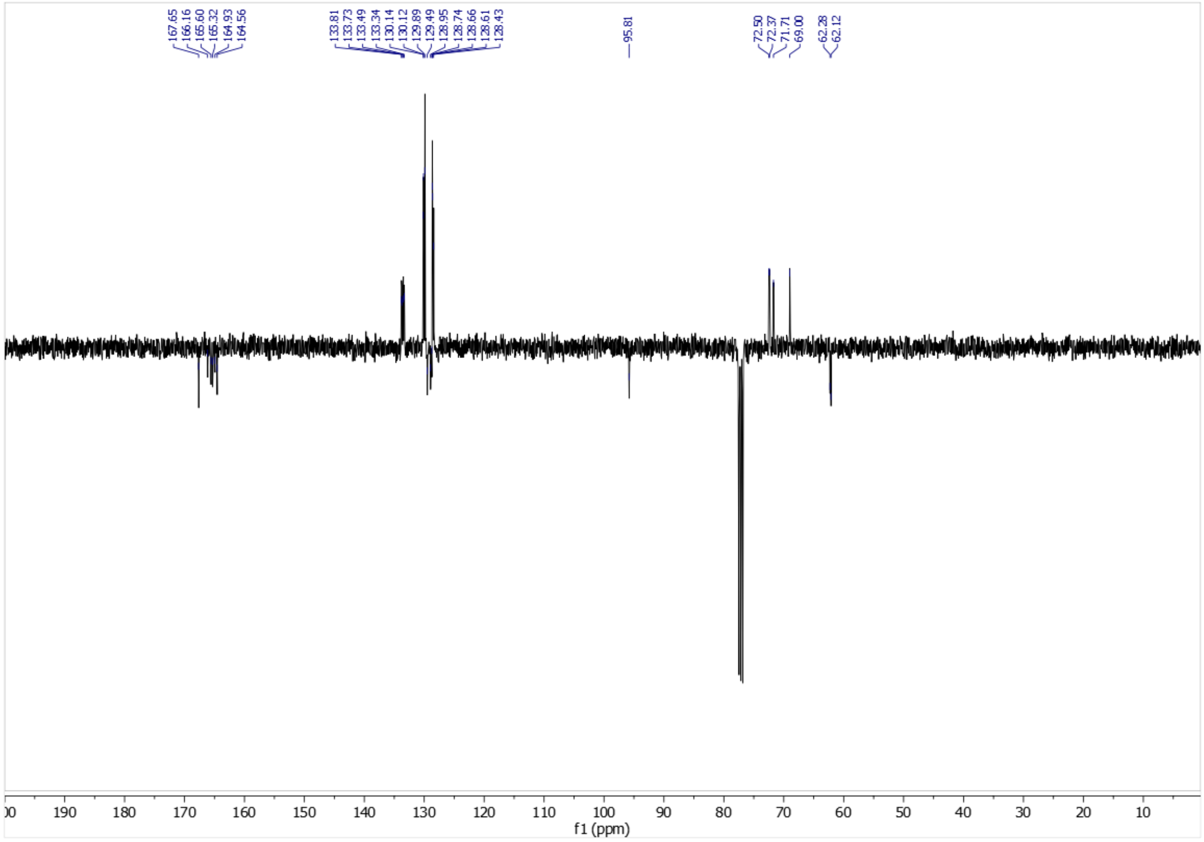

167.65
166.16
165.60
165.32
164.93
164.56
133.81
133.73
133.49
133.34
130.14
130.12
129.89
129.49
128.95
128.74
128.66
128.61
128.43
95.81
72.50
72.37
71.71
69.00
62.28
62.12
f1 (ppm)

^{1}H (400 MHz) and ^{13}C J-MOD (100 MHz) NMR spectra of compound **28** in $CDCl_3$

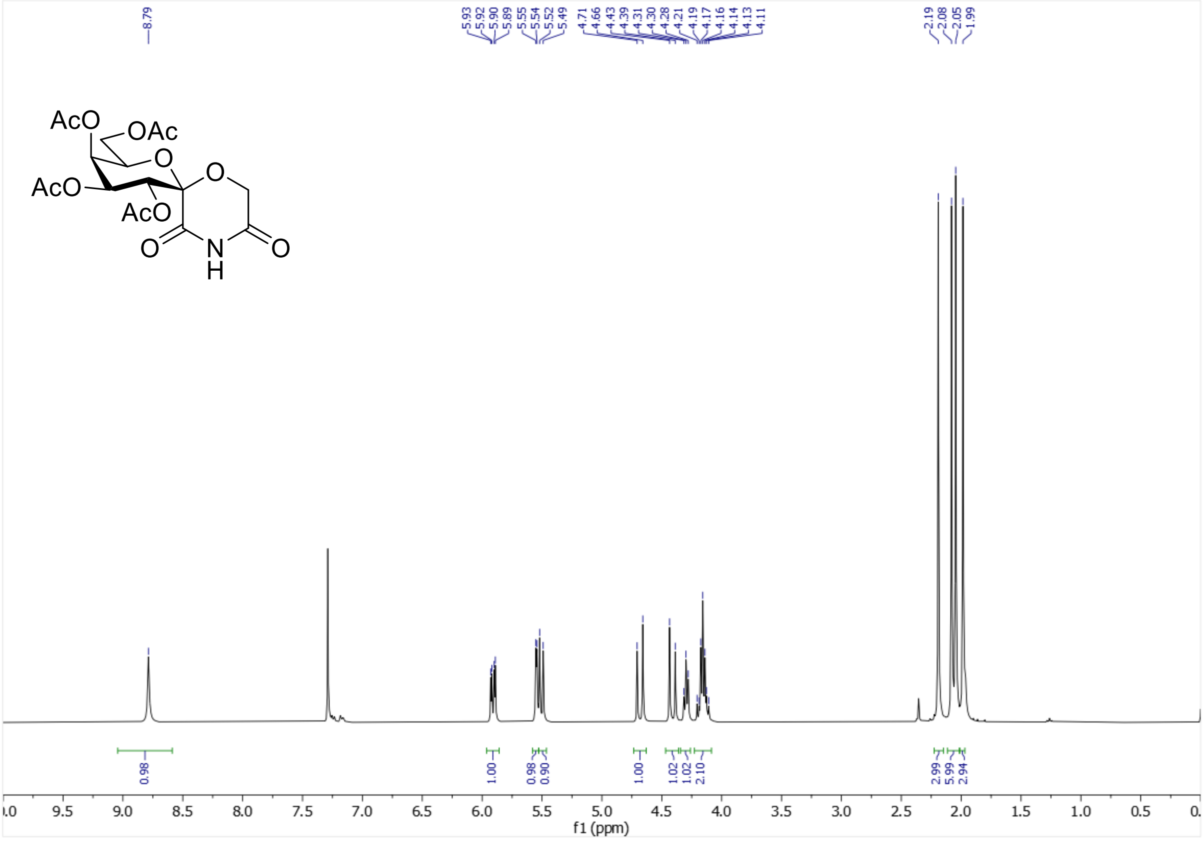

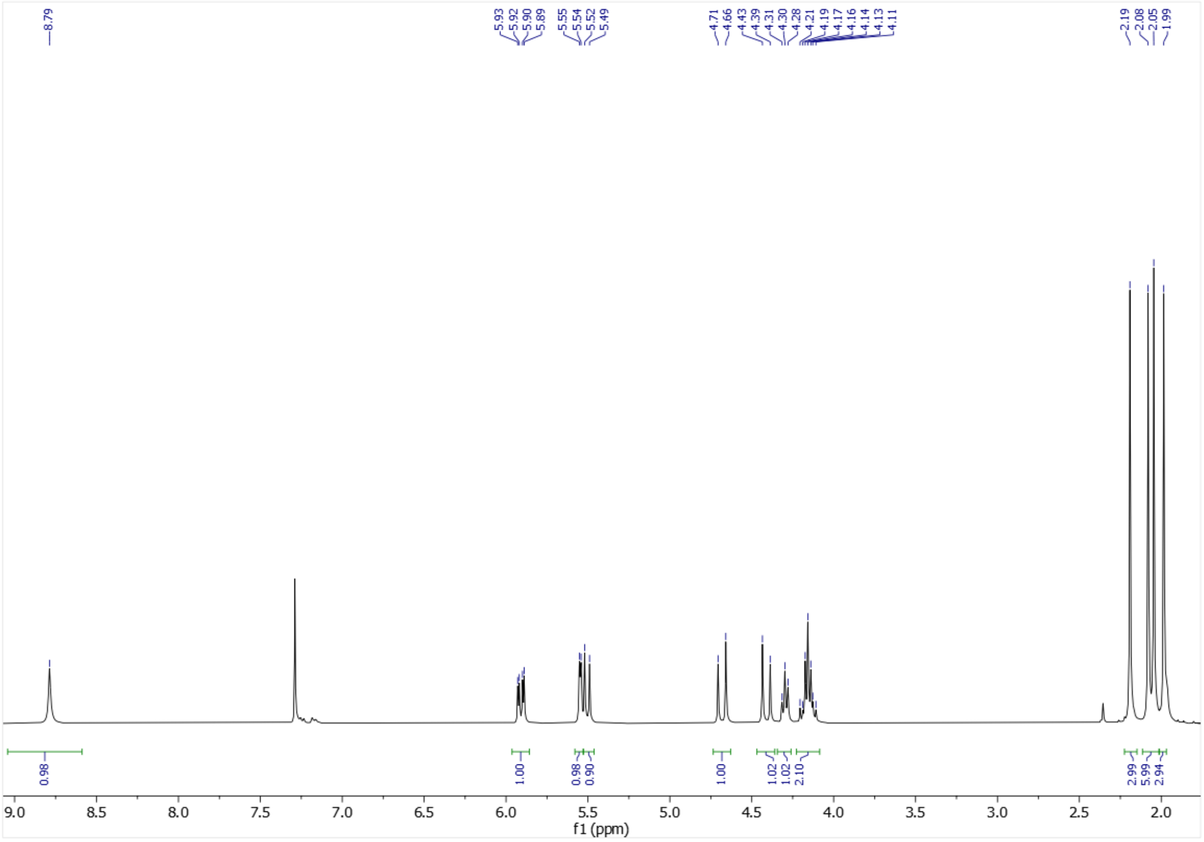

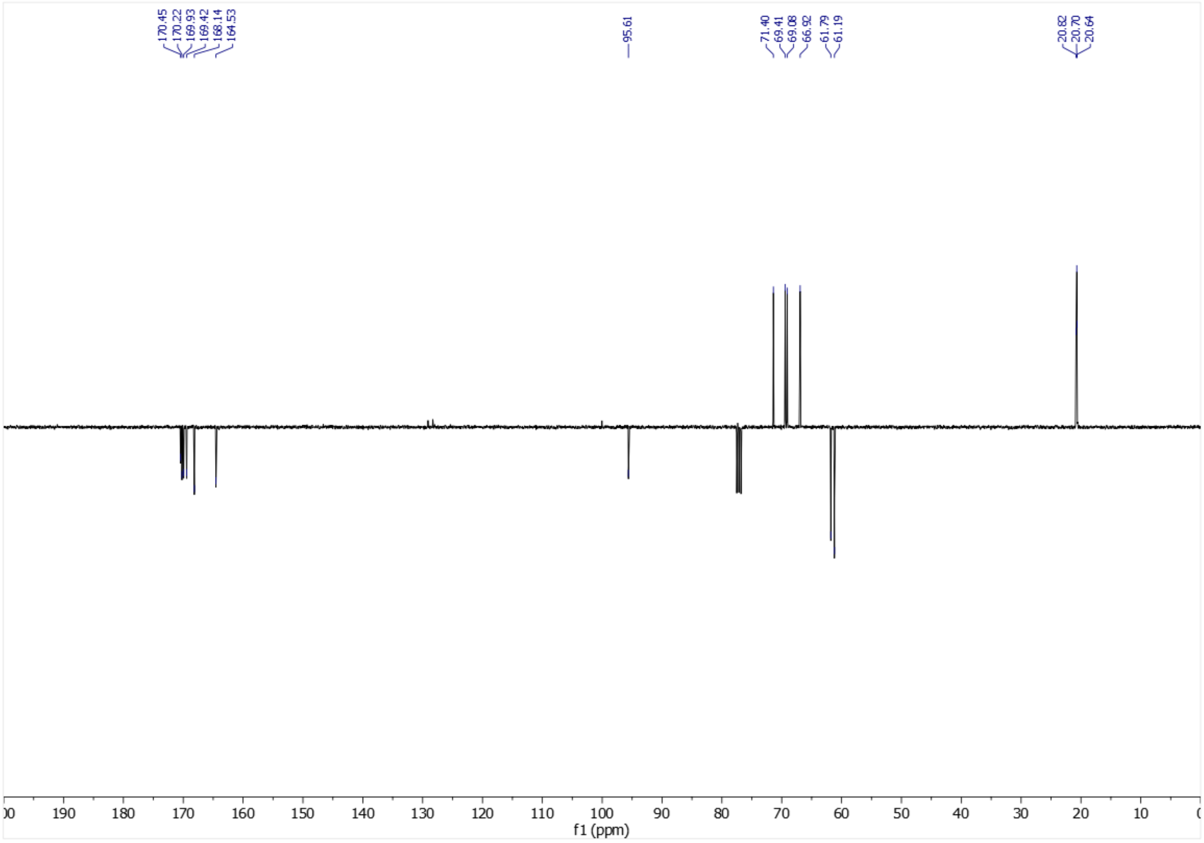
170.45
170.22
169.93
169.42
168.14
164.53
95.61
71.40
69.41
69.08
66.92
61.79
61.19
20.82
20.70
20.64
190
180
170
160
150
140
130
120
110
100
90
80
70
60
50
40
30
20
10
f1 (ppm)

^{1}H (400 MHz) and ^{13}C J-MOD (100 MHz) NMR spectra of compound **29** in DMSO-d6

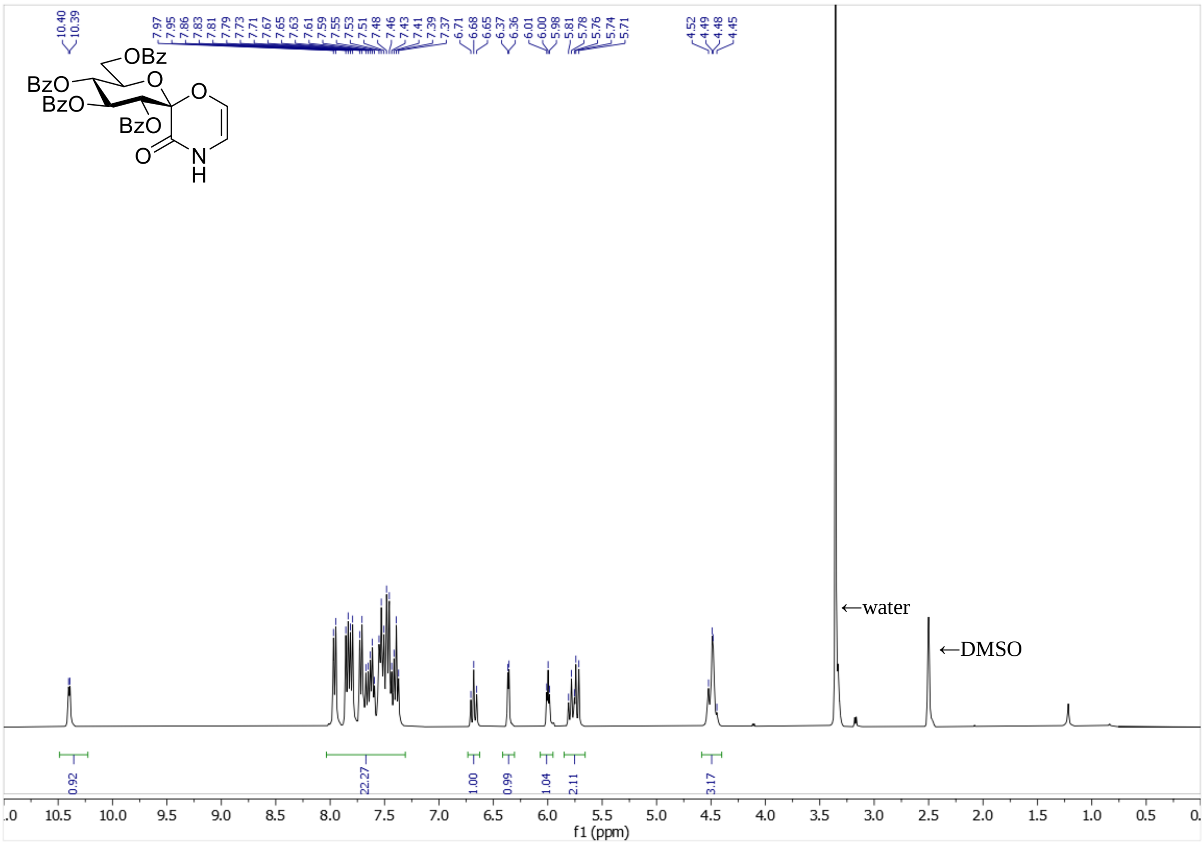

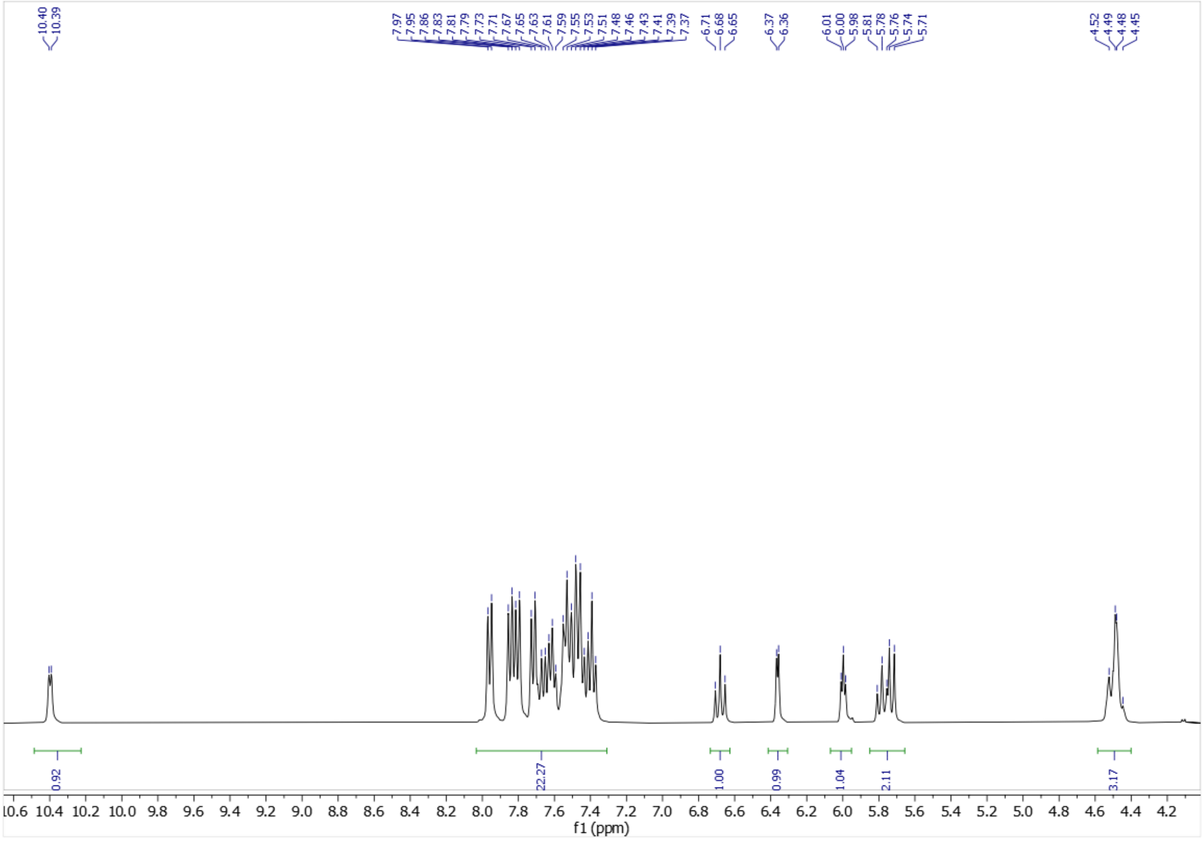

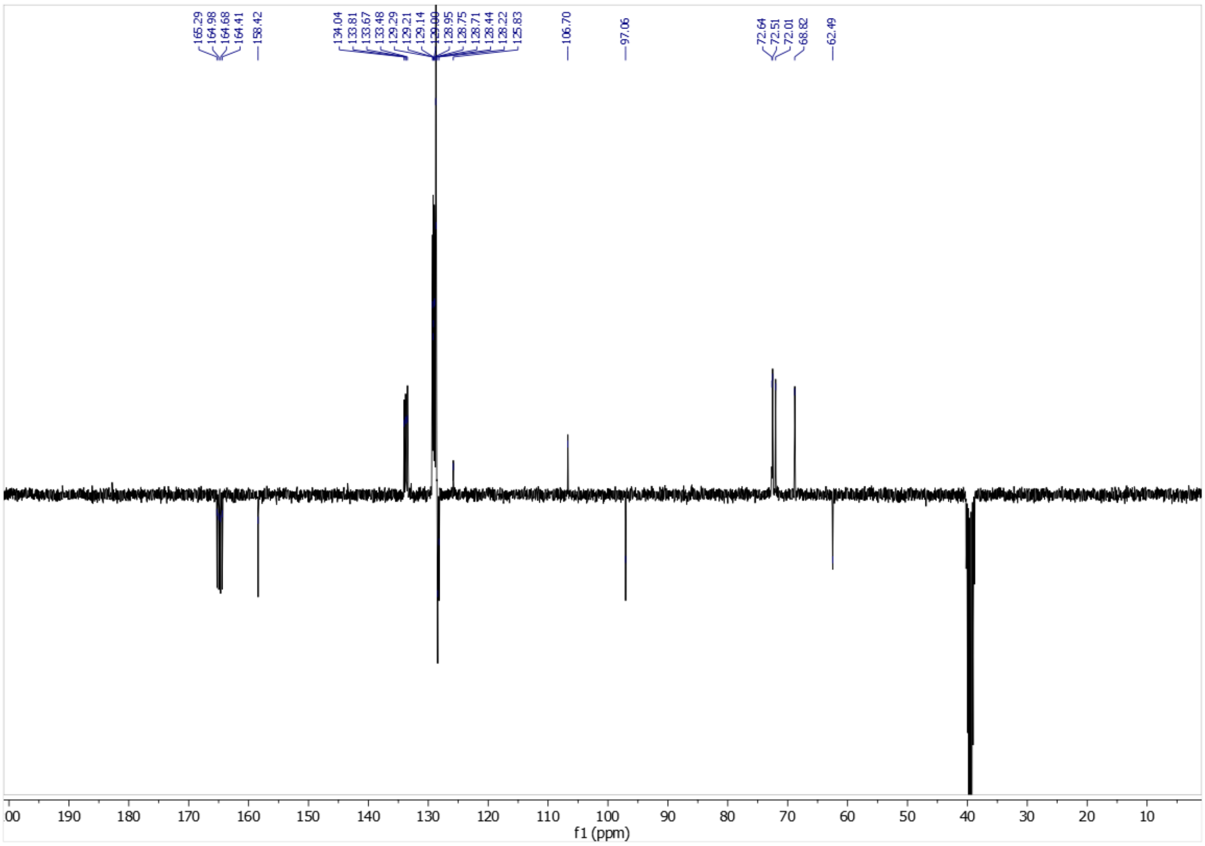
165.29
164.98
164.68
164.41
158.42
134.04
133.81
133.67
133.48
129.29
129.21
129.14
128.95
128.75
128.71
128.44
128.22
125.83
106.70
97.06
72.64
72.51
72.01
68.82
62.49
f1 (ppm)

^{1}H (400 MHz) and ^{13}C J-MOD (100 MHz) NMR spectra of compound **30** in $CDCl_3$

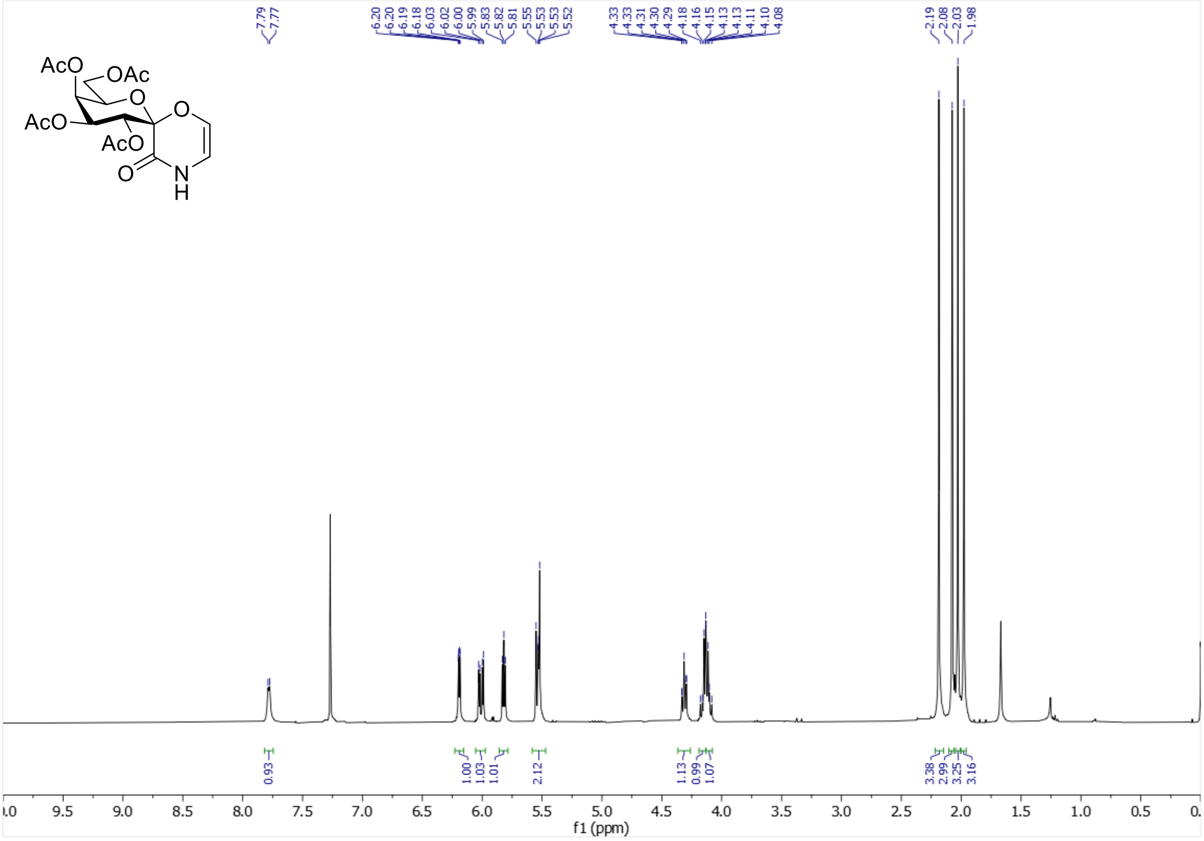

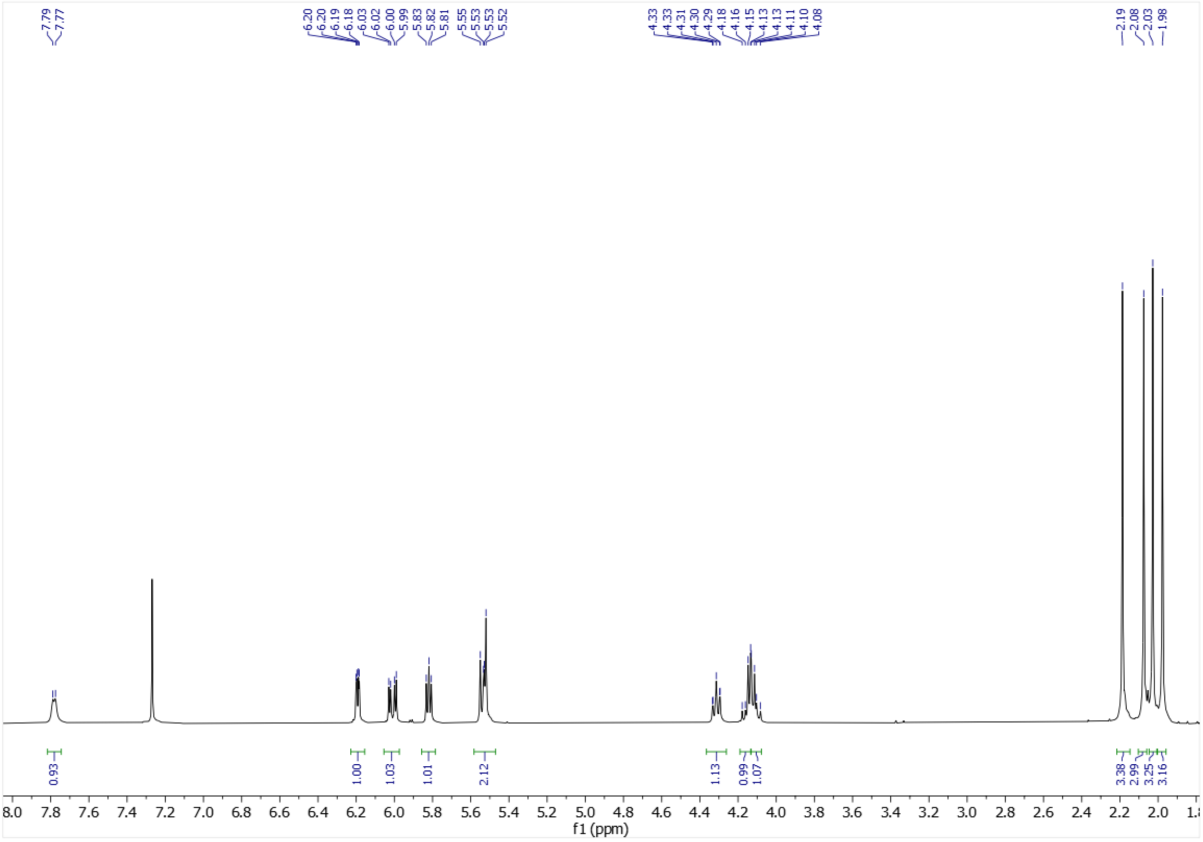

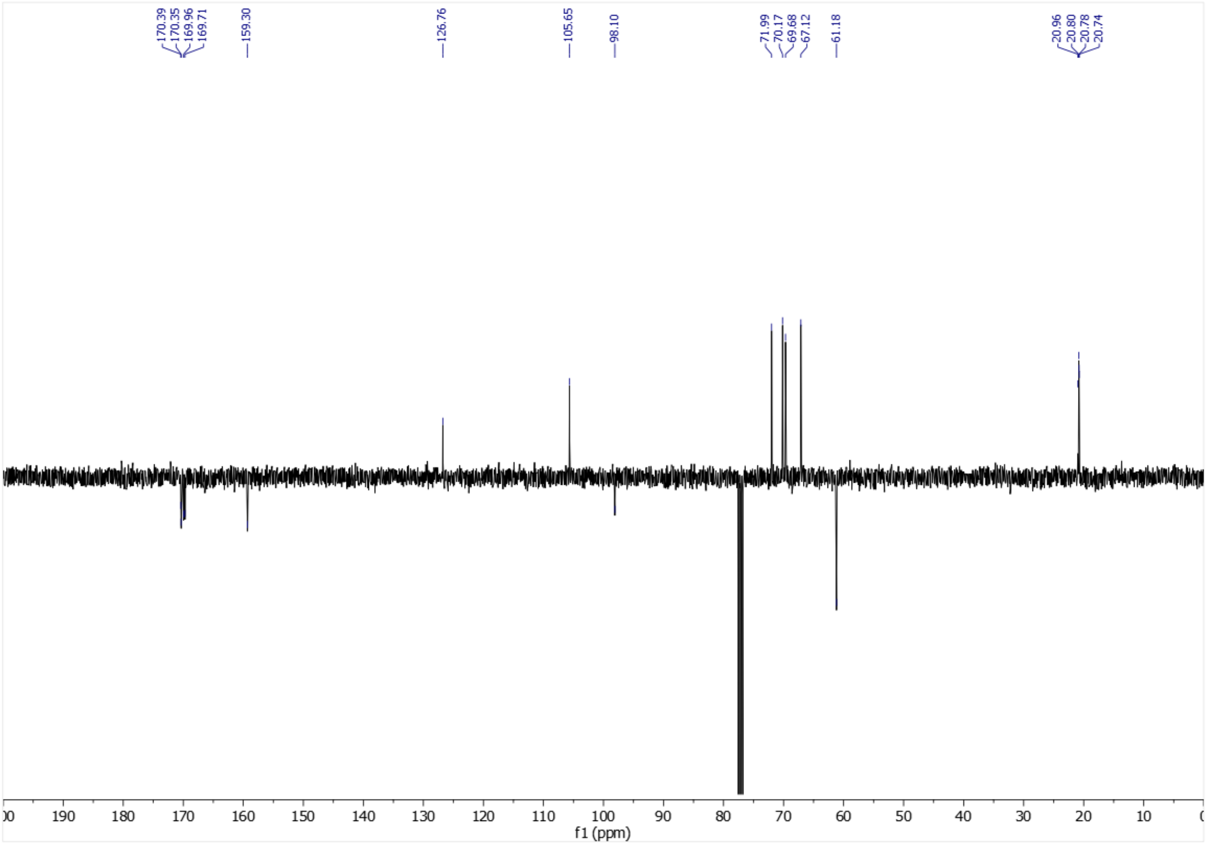

170.39
170.35
169.96
169.71
159.30
126.76
105.65
98.10
71.99
70.17
69.68
67.12
61.18
20.96
20.80
20.78
20.74
f1 (ppm)

^{1}H (400 MHz) and ^{13}C J-MOD (100 MHz) NMR spectra of compound **31** in $CDCl_3$

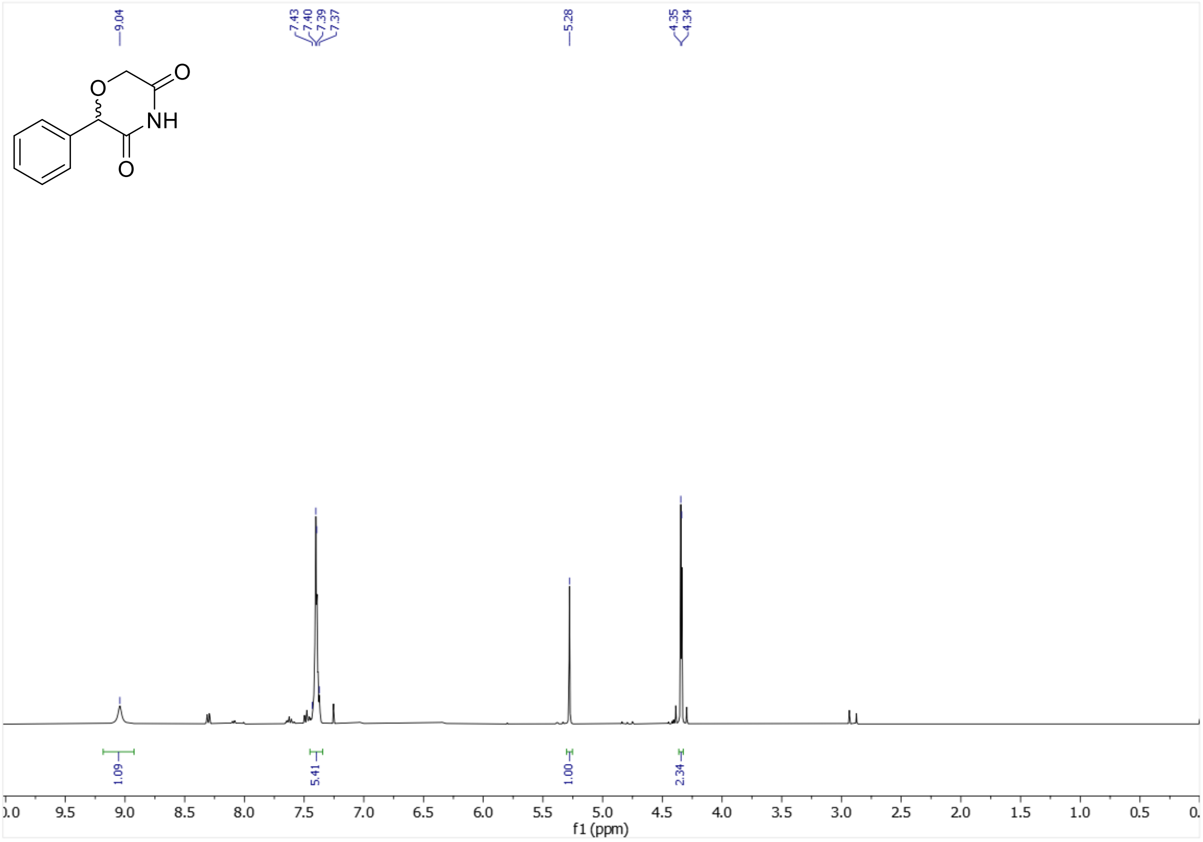

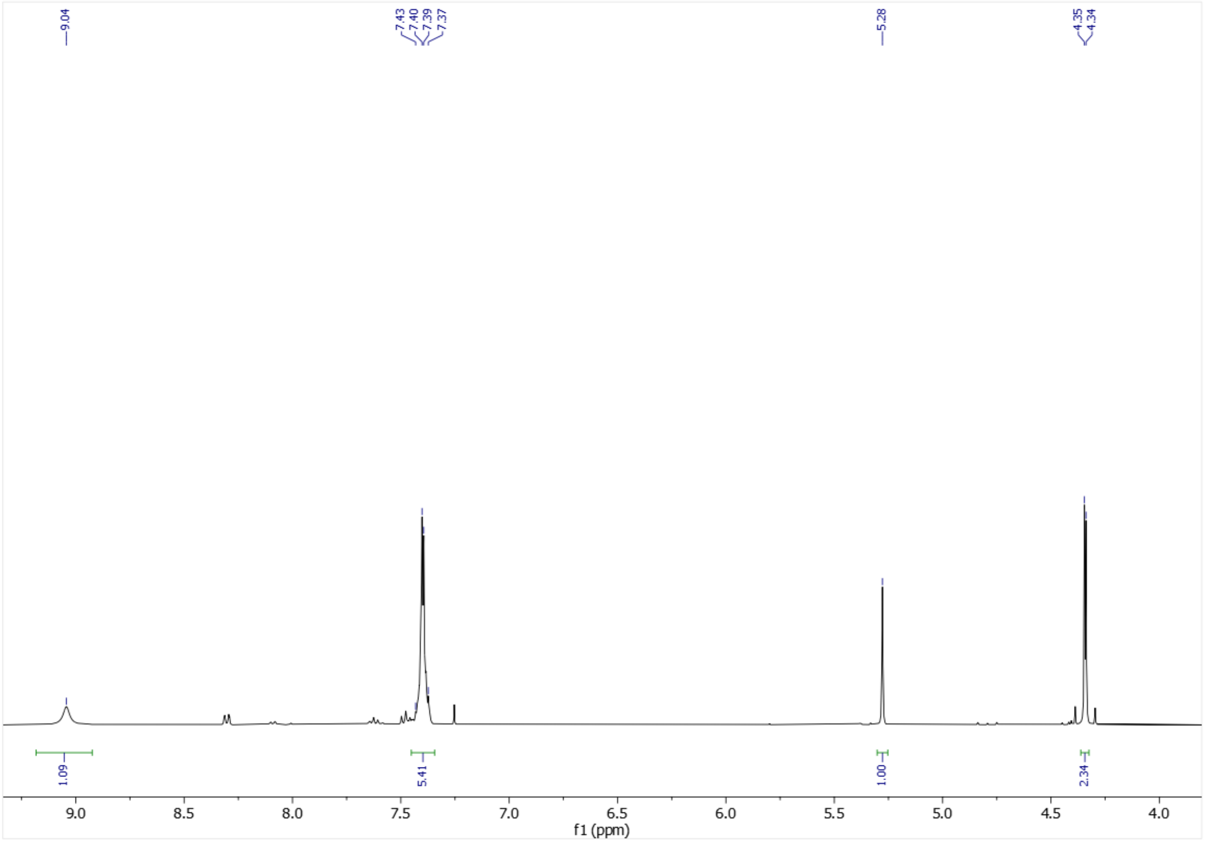

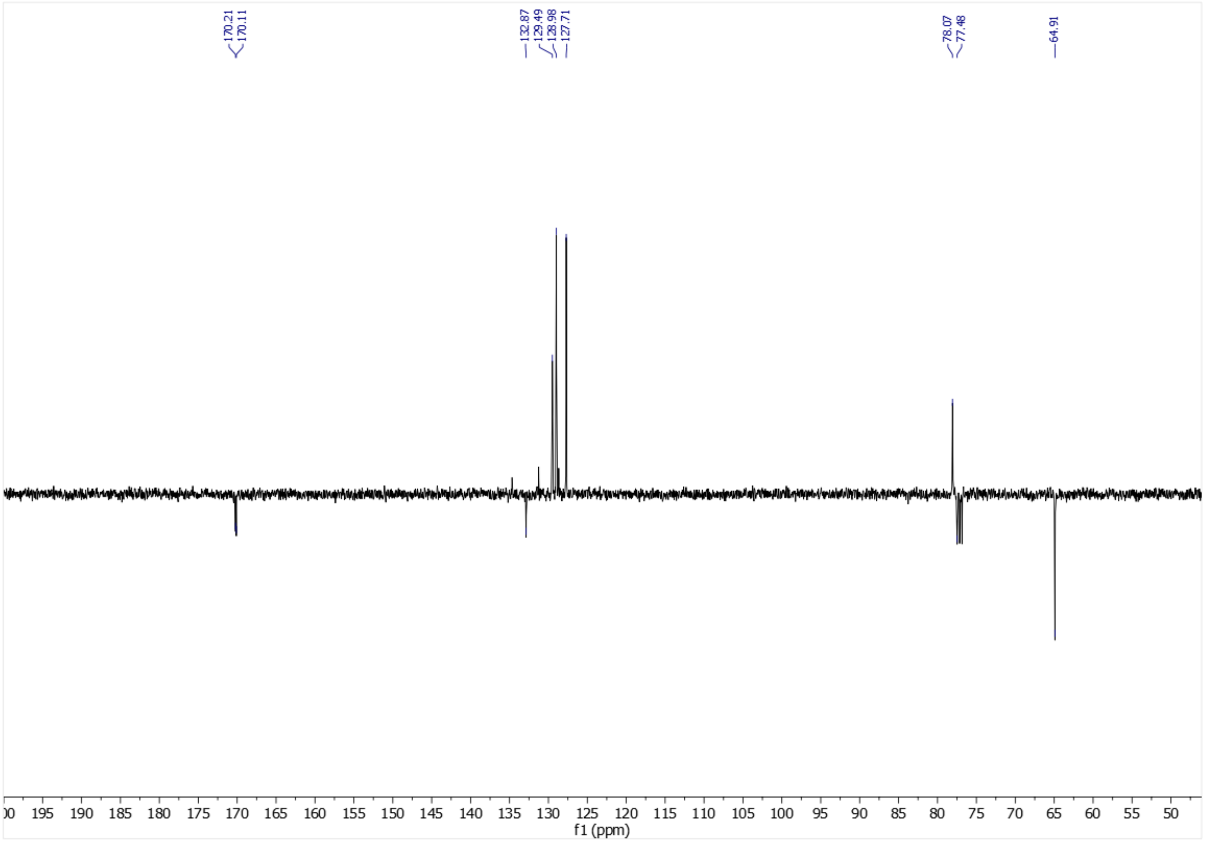
170.21
170.11
132.87
129.49
128.98
127.71
78.07
77.48
64.91
00 195 190 185 180 175 170 165 160 155 150 145 140 135 130 125 120 115 110 105 100 95 90 85 80 75 70 65 60 55 50
f1 (ppm)

¹H (360 MHz) and ¹³C J-MOD (90 MHz) NMR spectra of compound **32** in $CDCl_3$

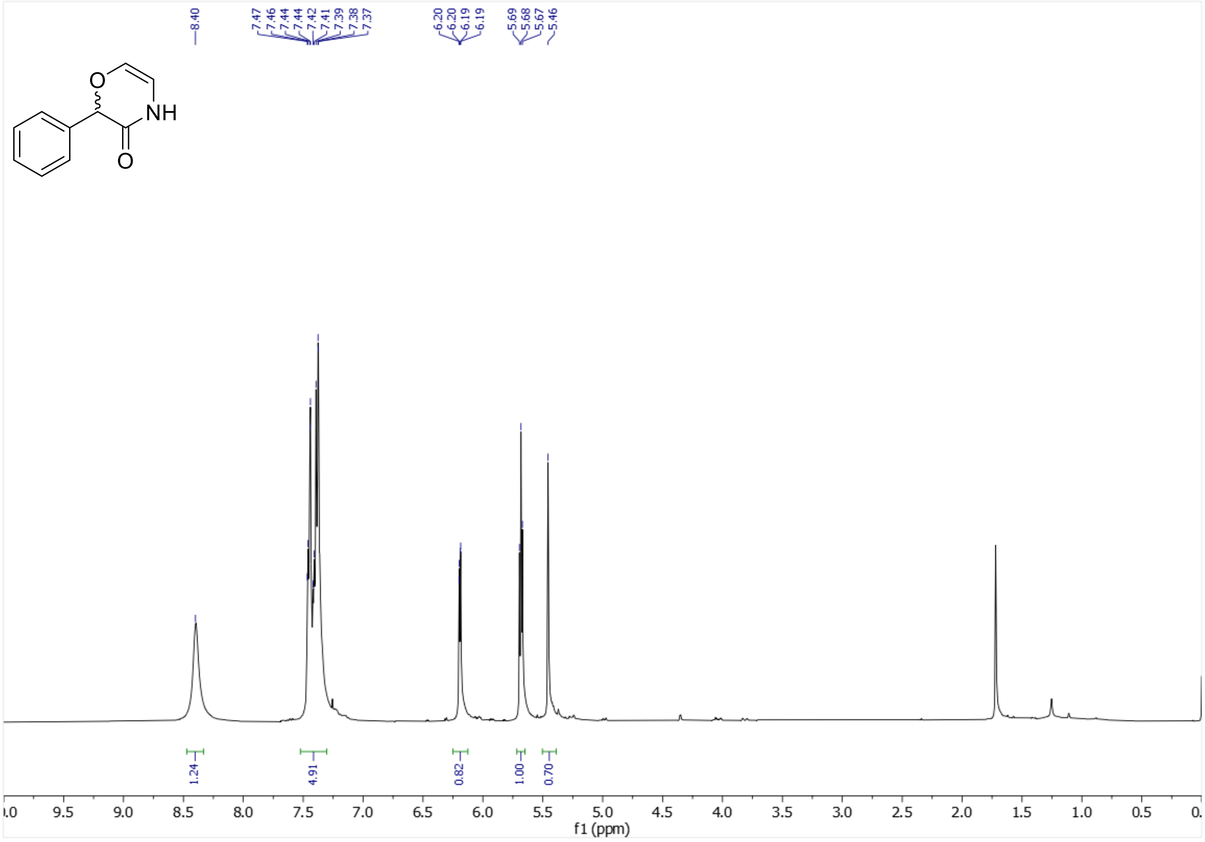

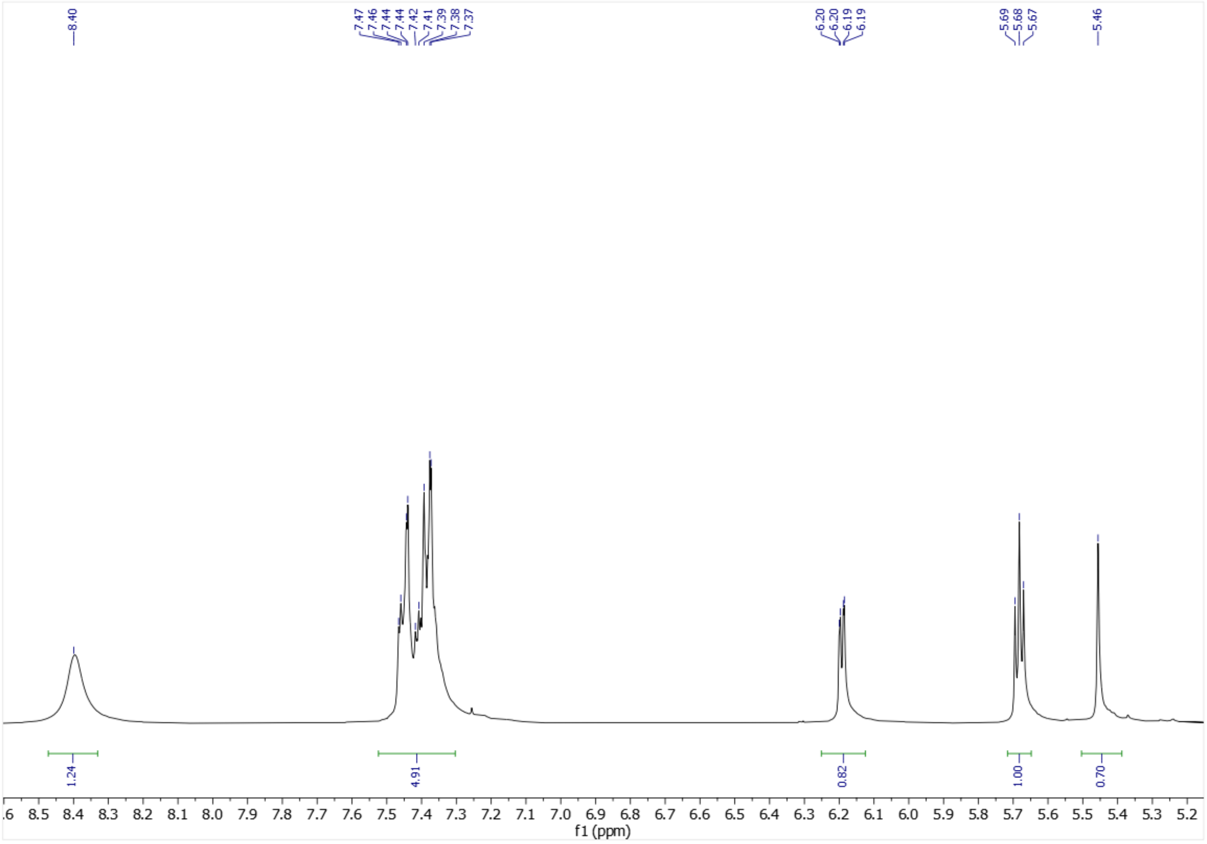

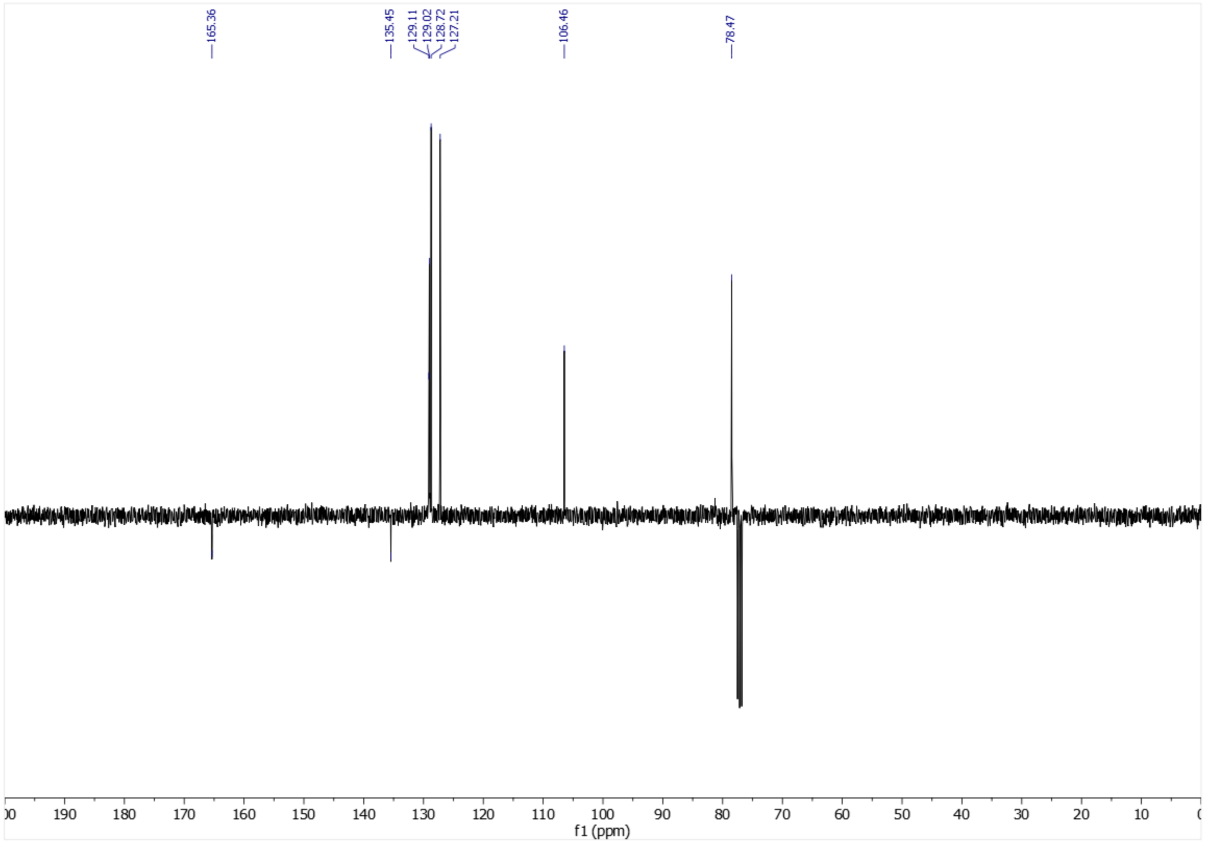
165.36
135.45
129.11
129.02
128.72
127.21
106.46
78.47
f1 (ppm)

^{1}H (400 MHz) and ^{13}C J-MOD (100 MHz) NMR spectra of compound **33** in $CDCl_3$

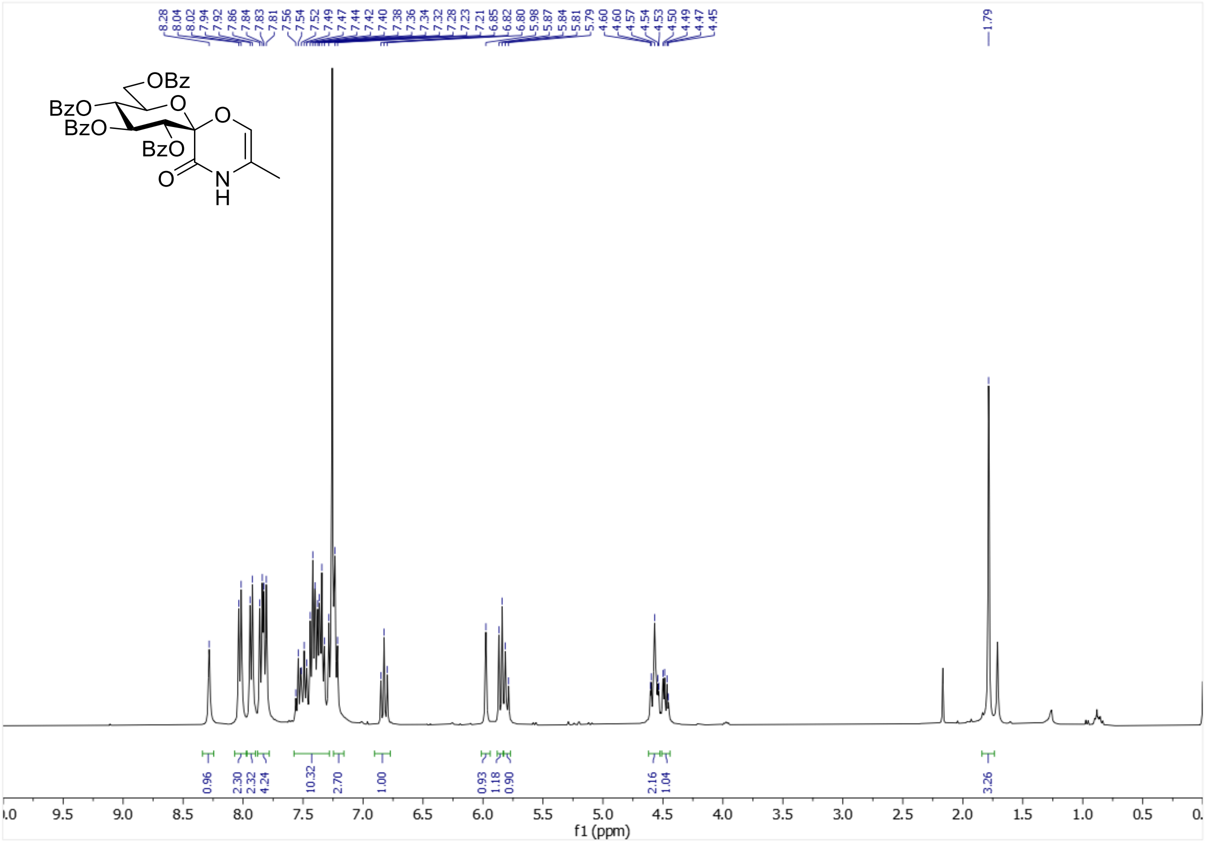

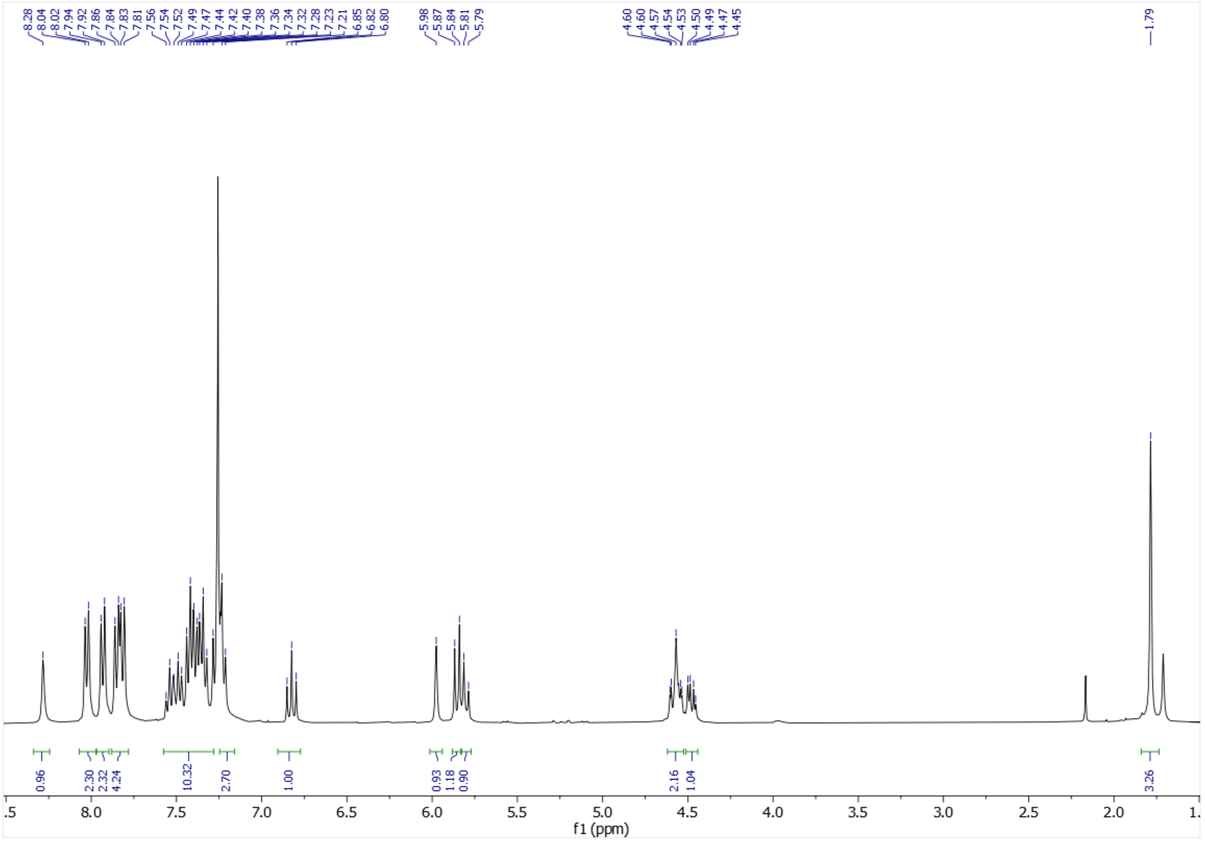

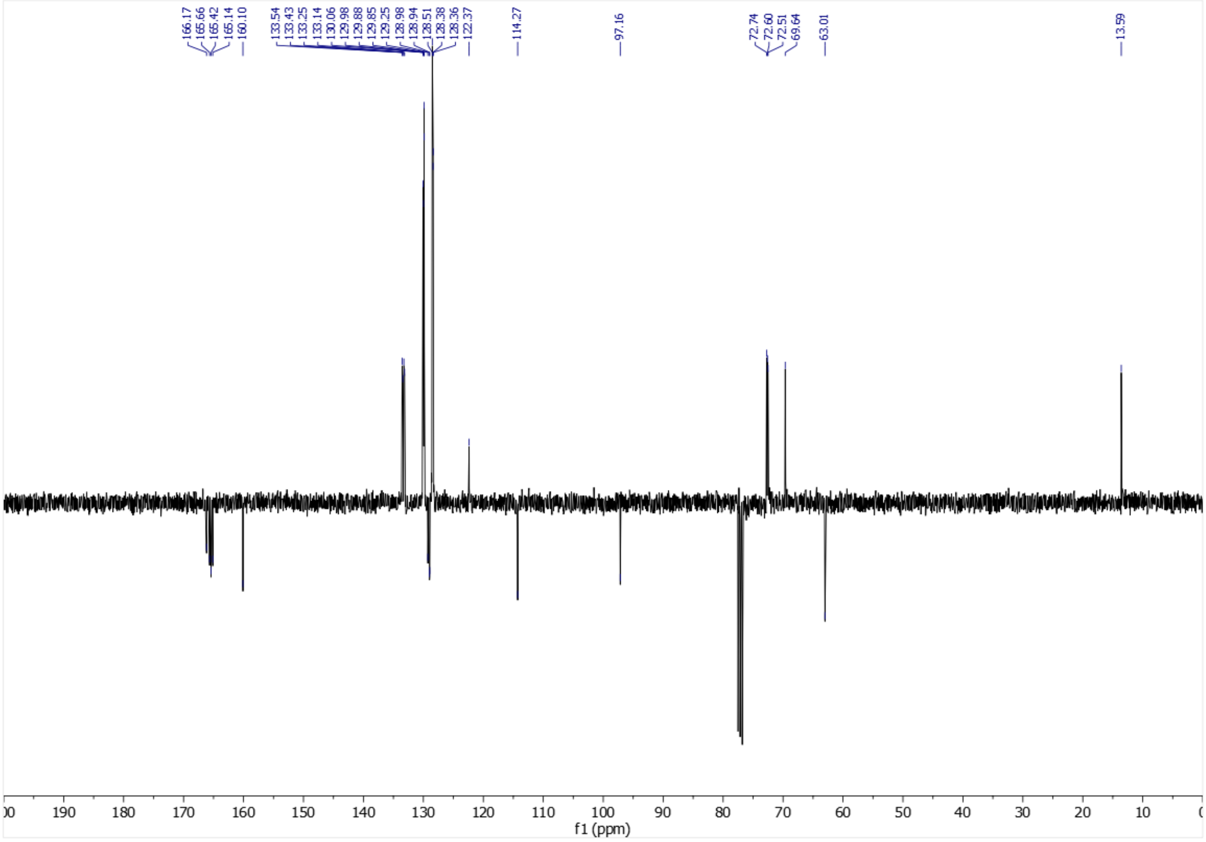

166.17
165.66
165.42
165.14
160.10
133.54
133.43
133.25
133.14
130.06
129.98
129.88
129.85
129.25
128.98
128.94
128.51
128.38
128.36
122.37
114.27
97.16
72.74
72.60
72.51
69.64
63.01
13.59
f1 (ppm)

^{1}H (400 MHz) and ^{13}C J-MOD (100 MHz) NMR spectra of compound **34** in CD_3OD

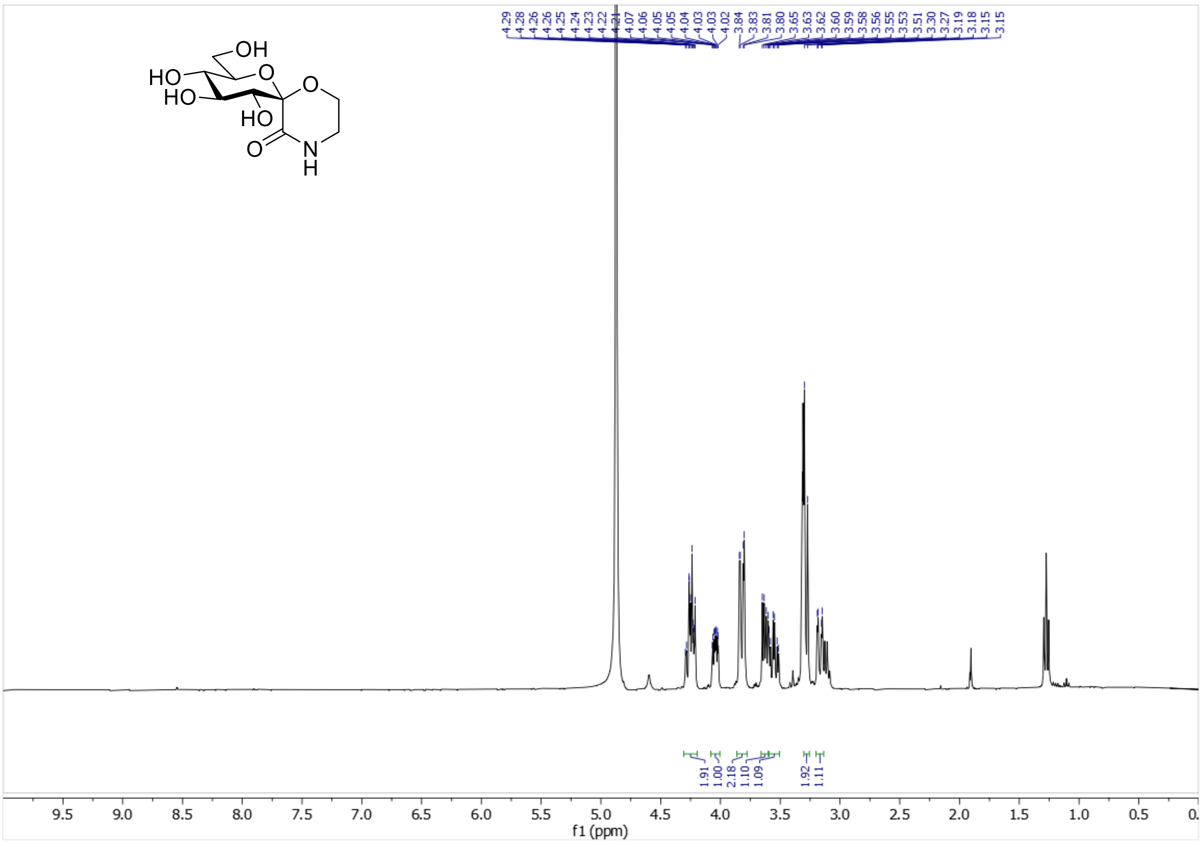

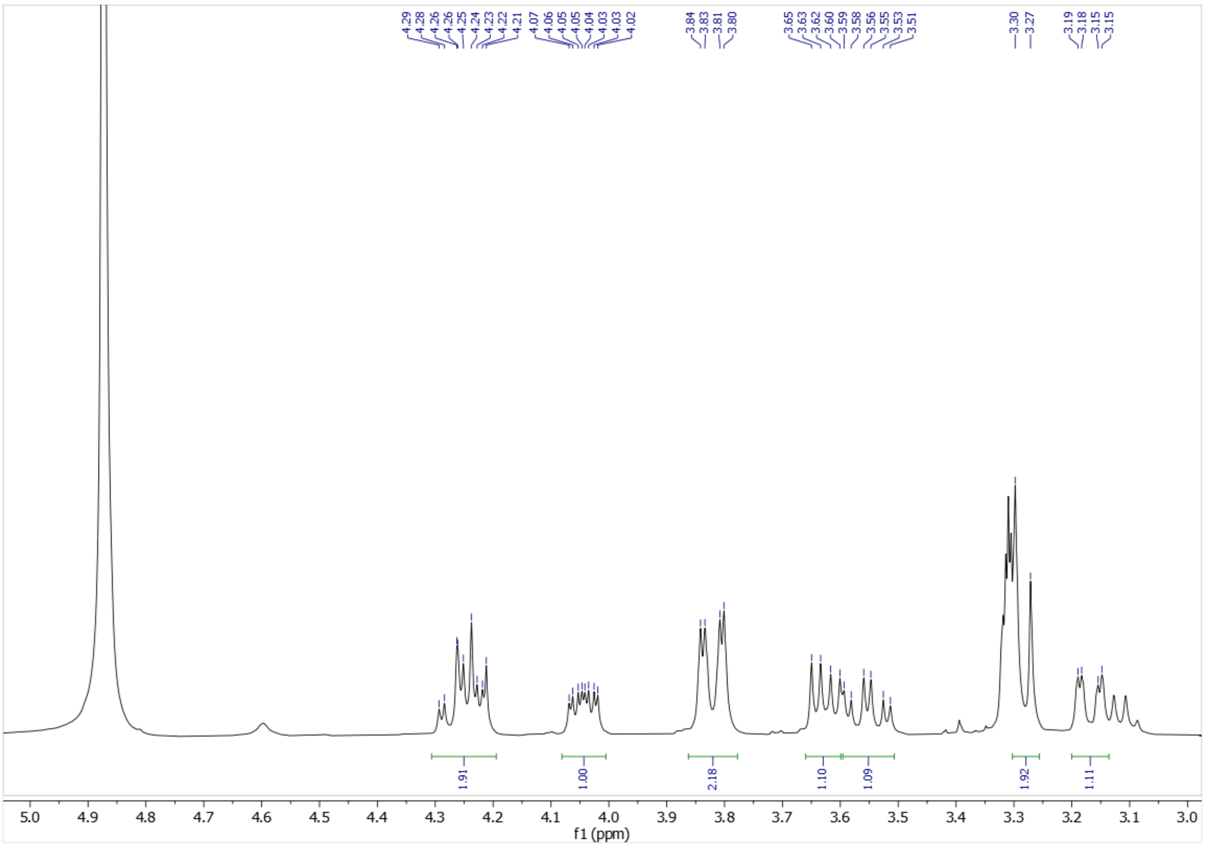

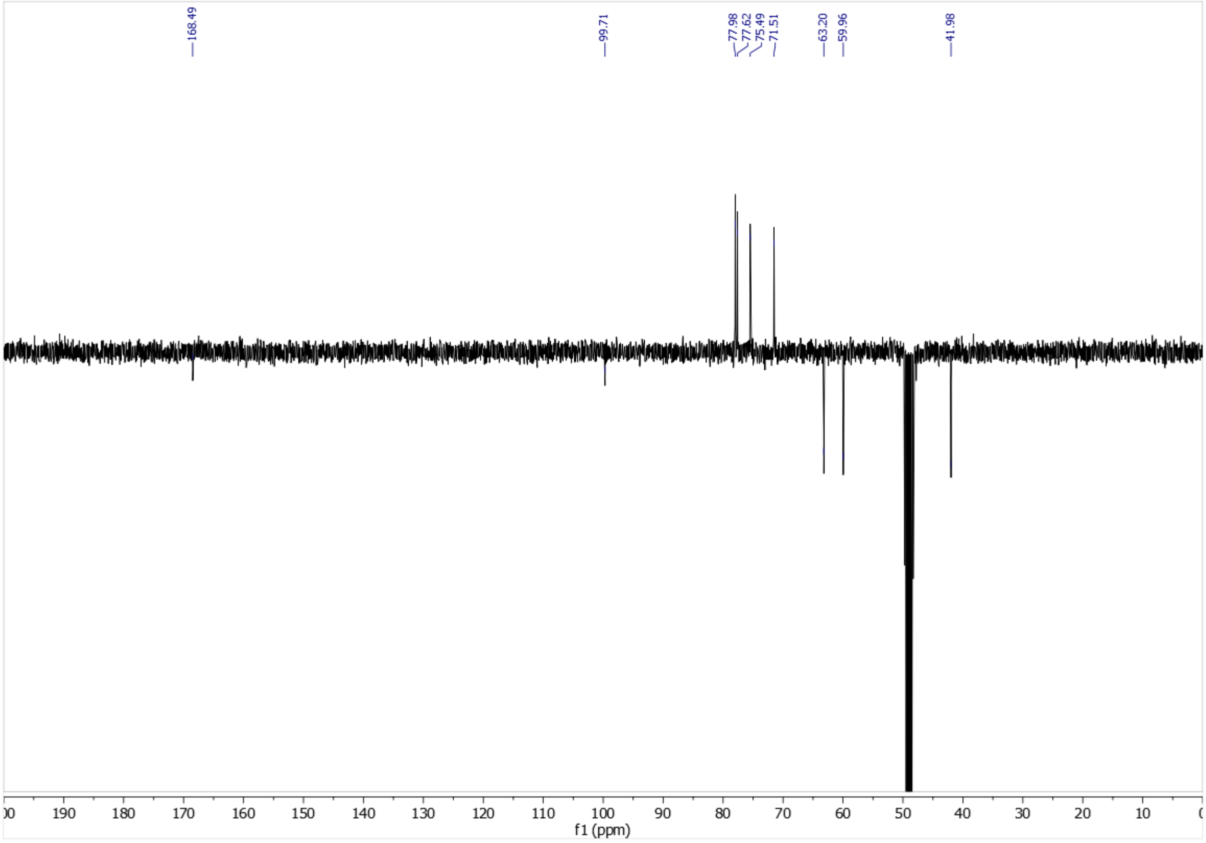

168.49
99.71
77.98
77.62
75.49
71.51
63.20
59.96
41.98
190
180
170
160
150
140
130
120
110
100
90
80
70
60
50
40
30
20
10
f1 (ppm)

^{1}H (400 MHz) and ^{13}C J-MOD (100 MHz) NMR spectra of compound **35** in CD_3OD

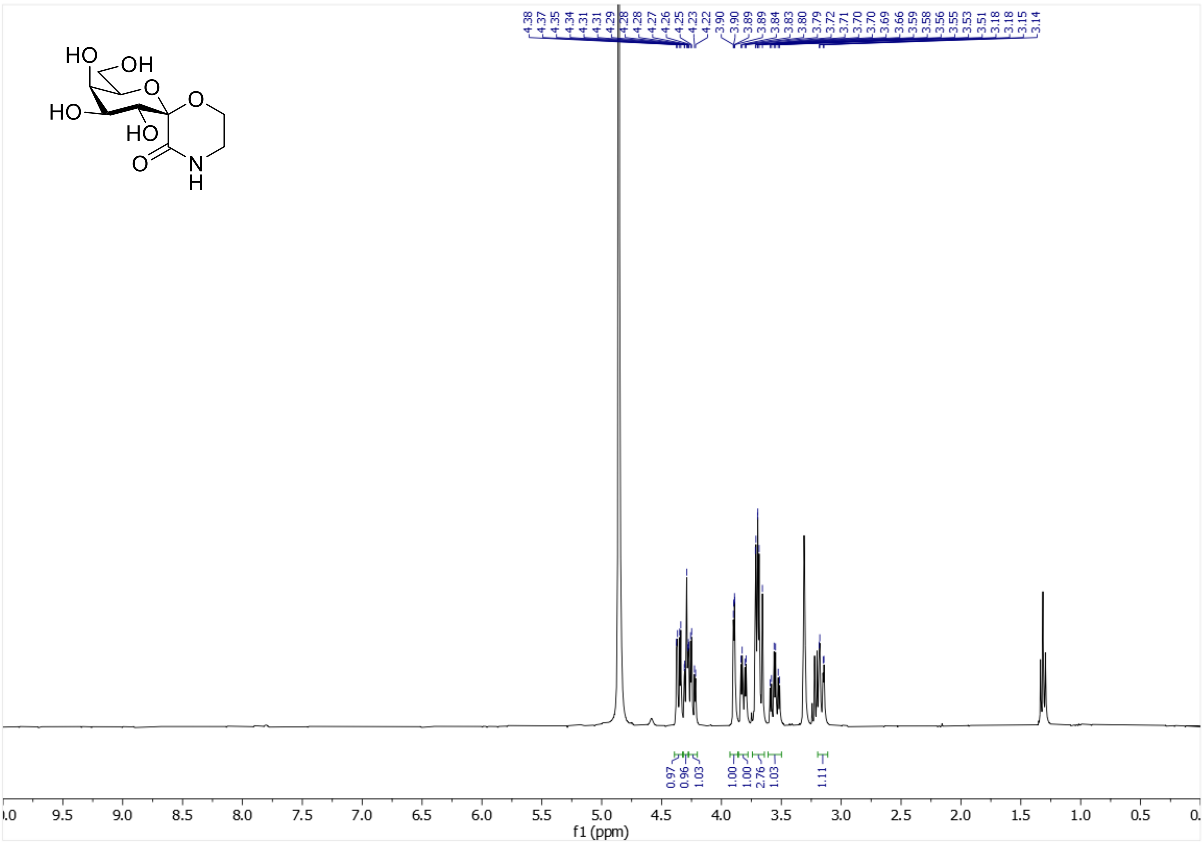

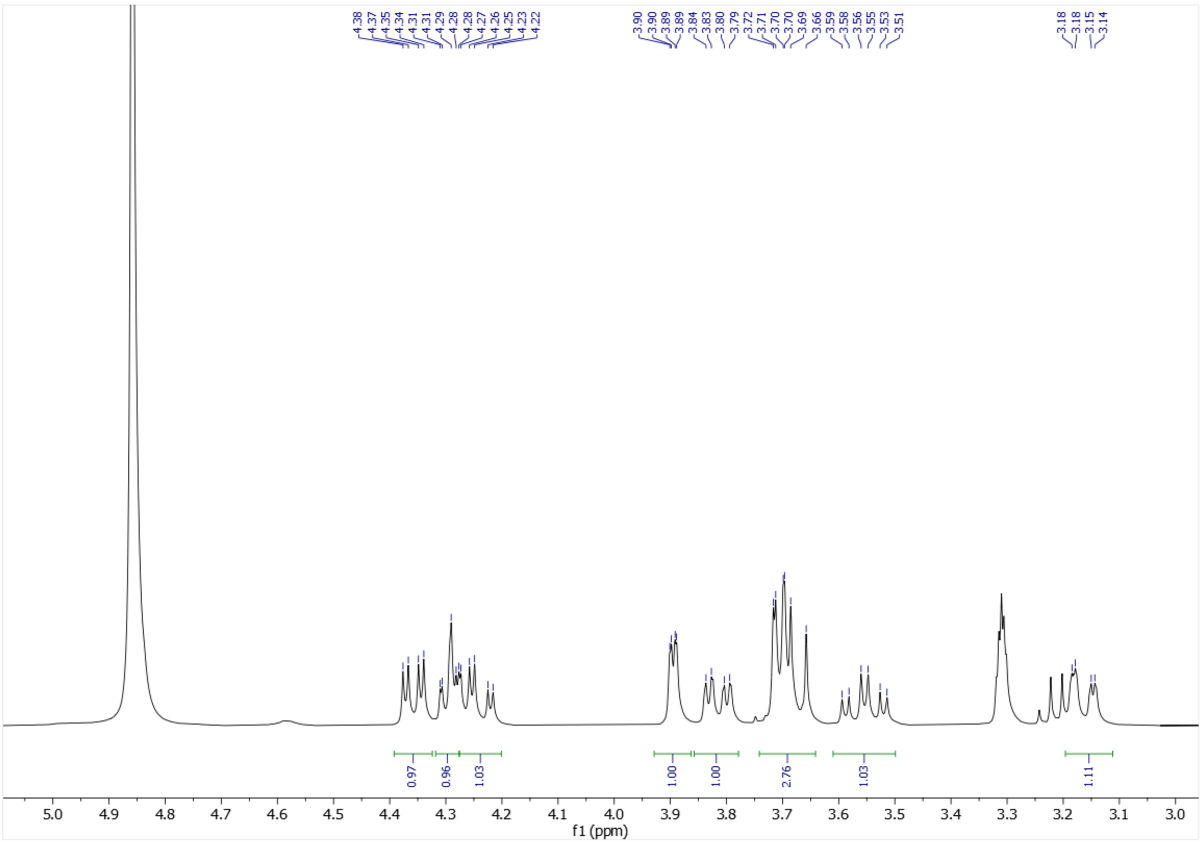

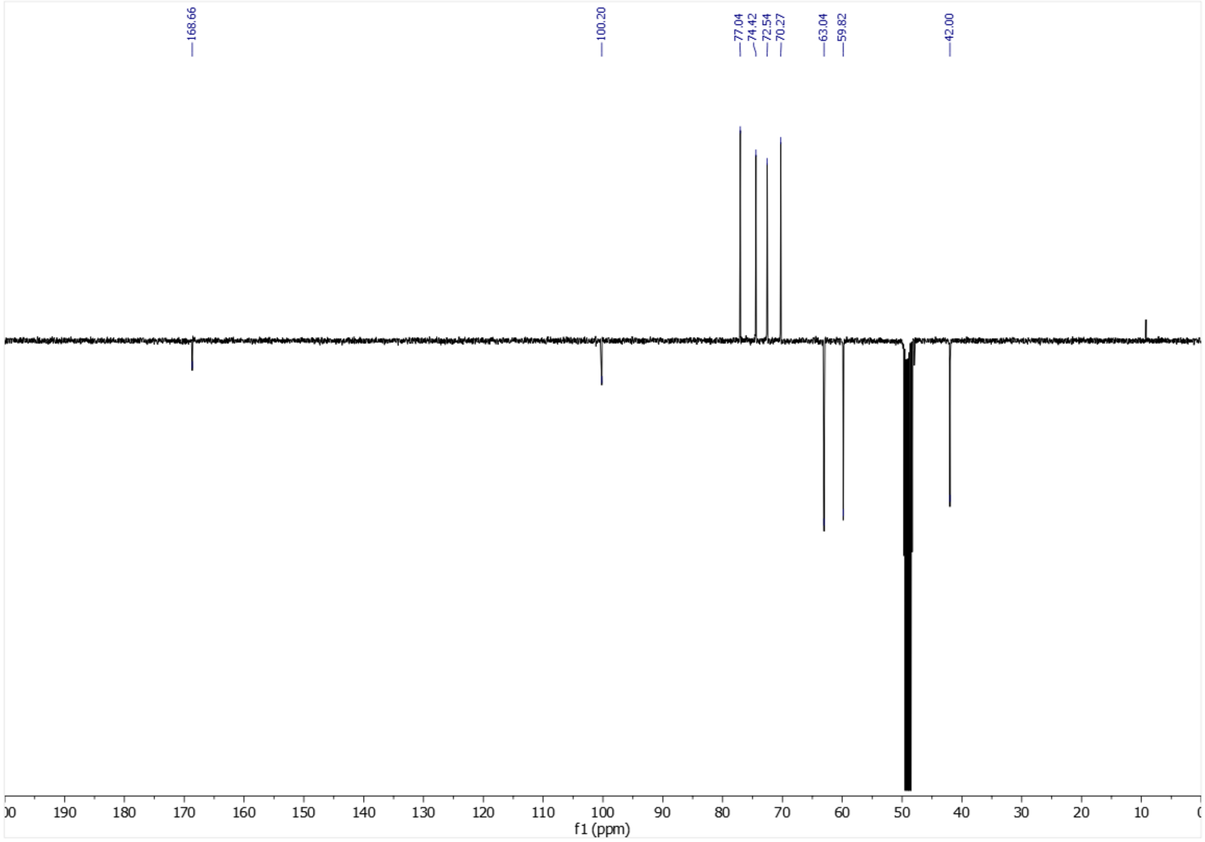

168.66
100.20
77.04
74.42
72.54
70.27
63.04
59.82
42.00
f1 (ppm)

^{1}H (400 MHz) and ^{13}C J-MOD (100 MHz) NMR spectra of compound **36** in CD_3OD

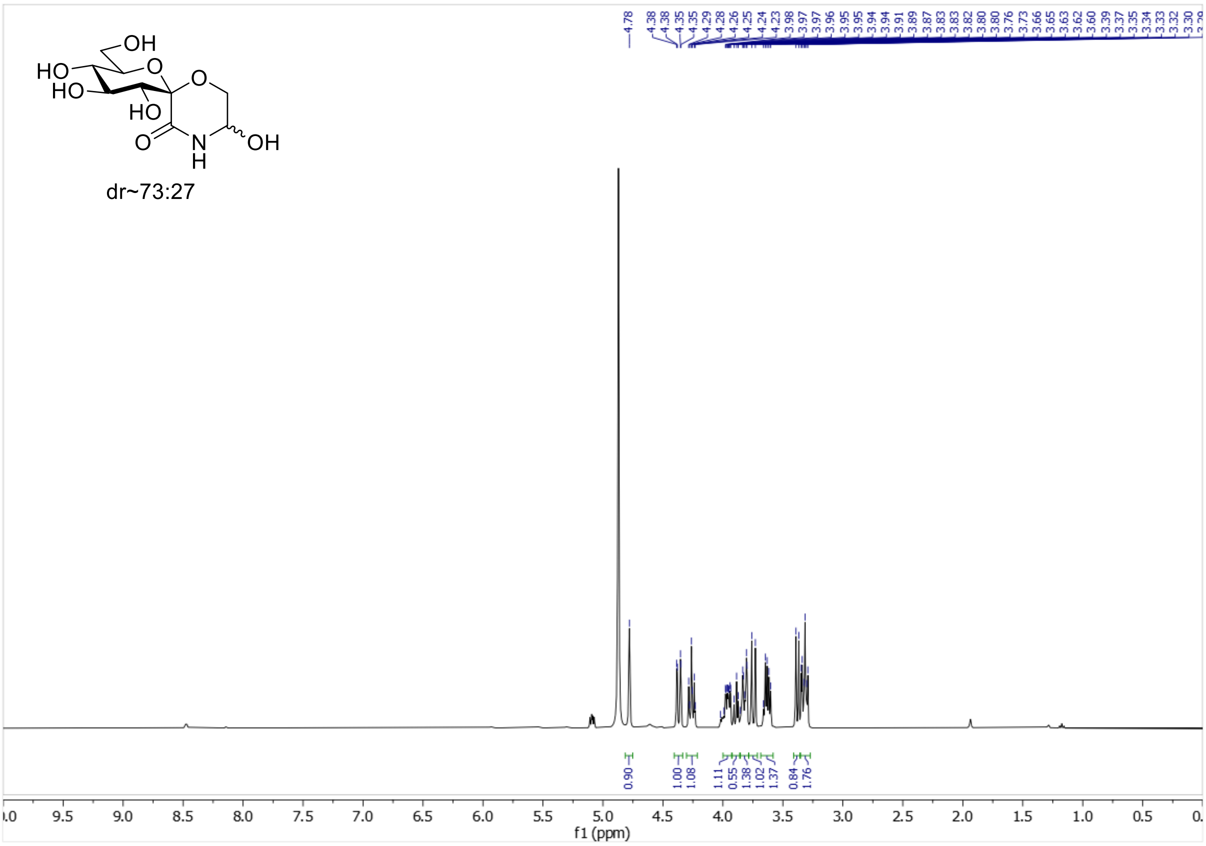

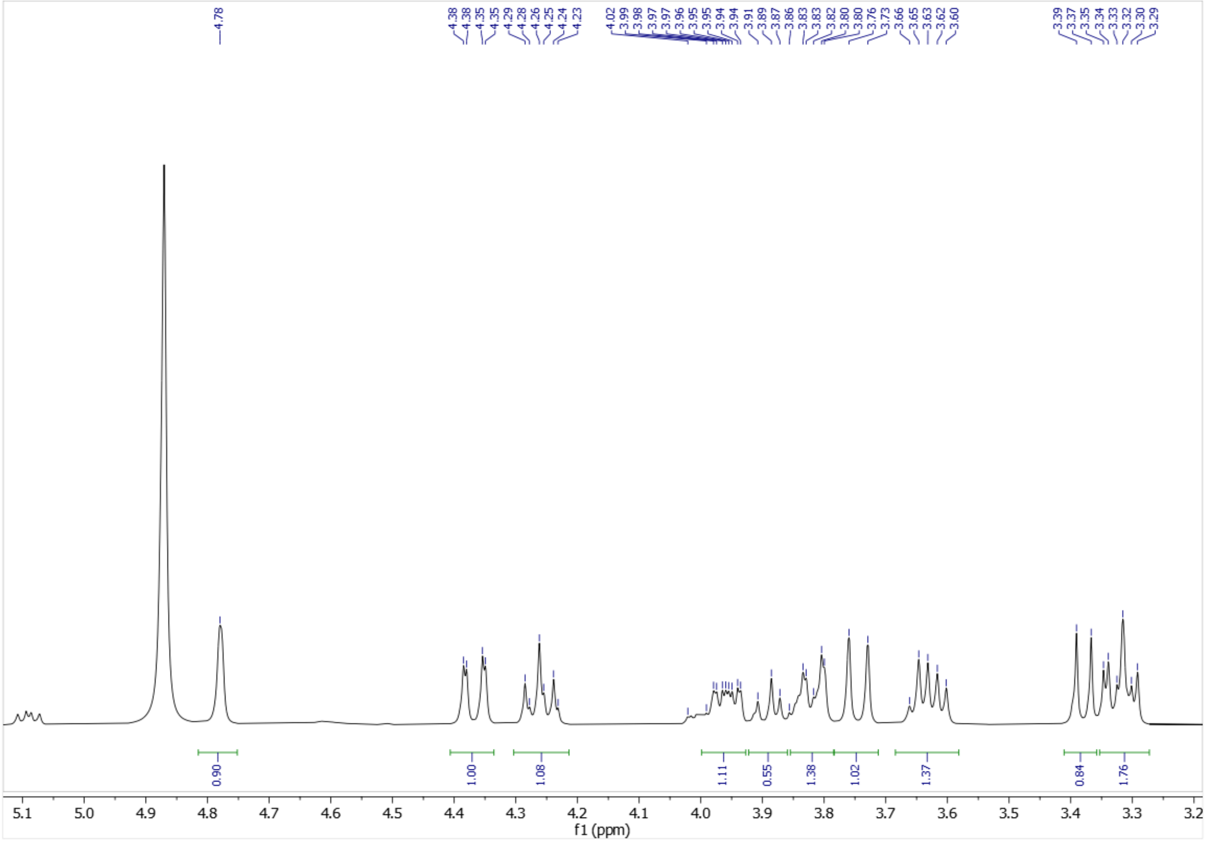

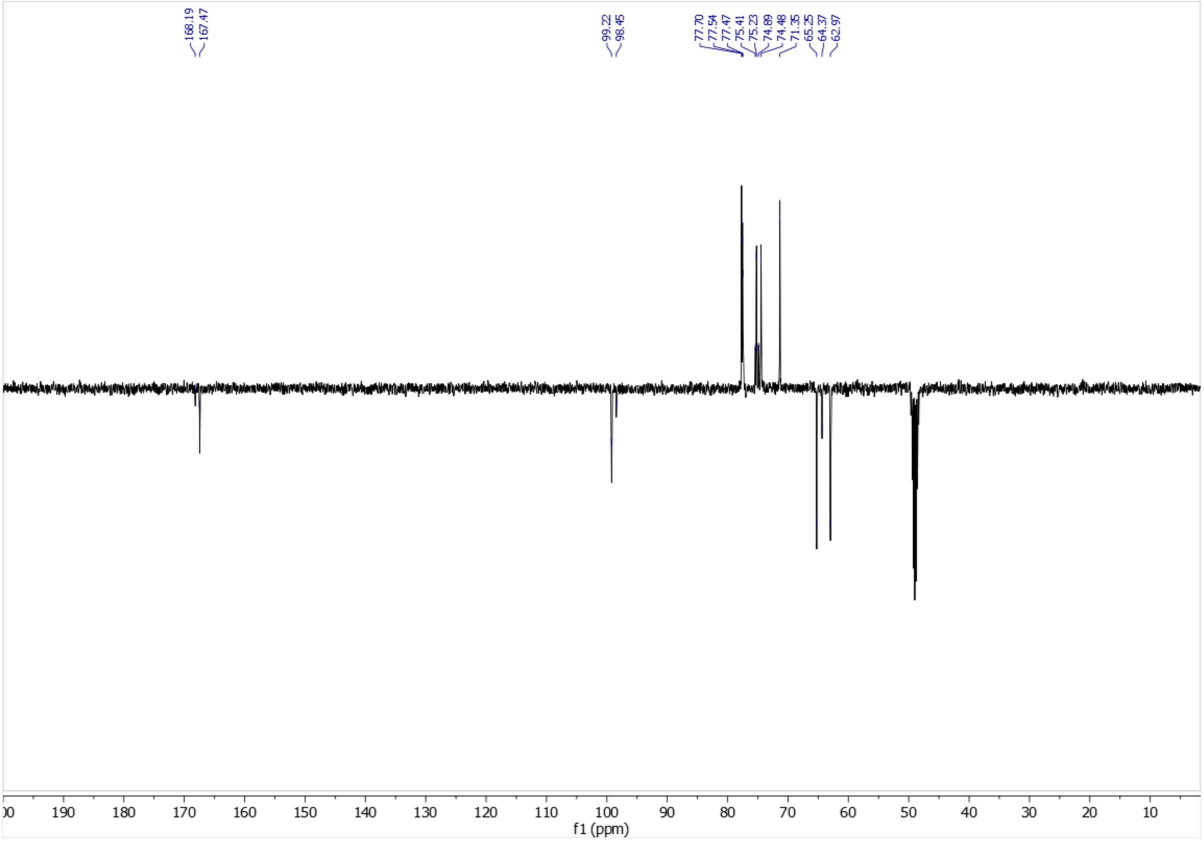

168.19
167.47
99.22
98.45
77.70
77.54
77.47
75.41
75.23
74.89
74.48
71.35
65.25
64.37
62.97
f1 (ppm)

^{1}H (360 MHz) and ^{13}C J-MOD (90 MHz) NMR spectra of compound **37** in CD_3OD

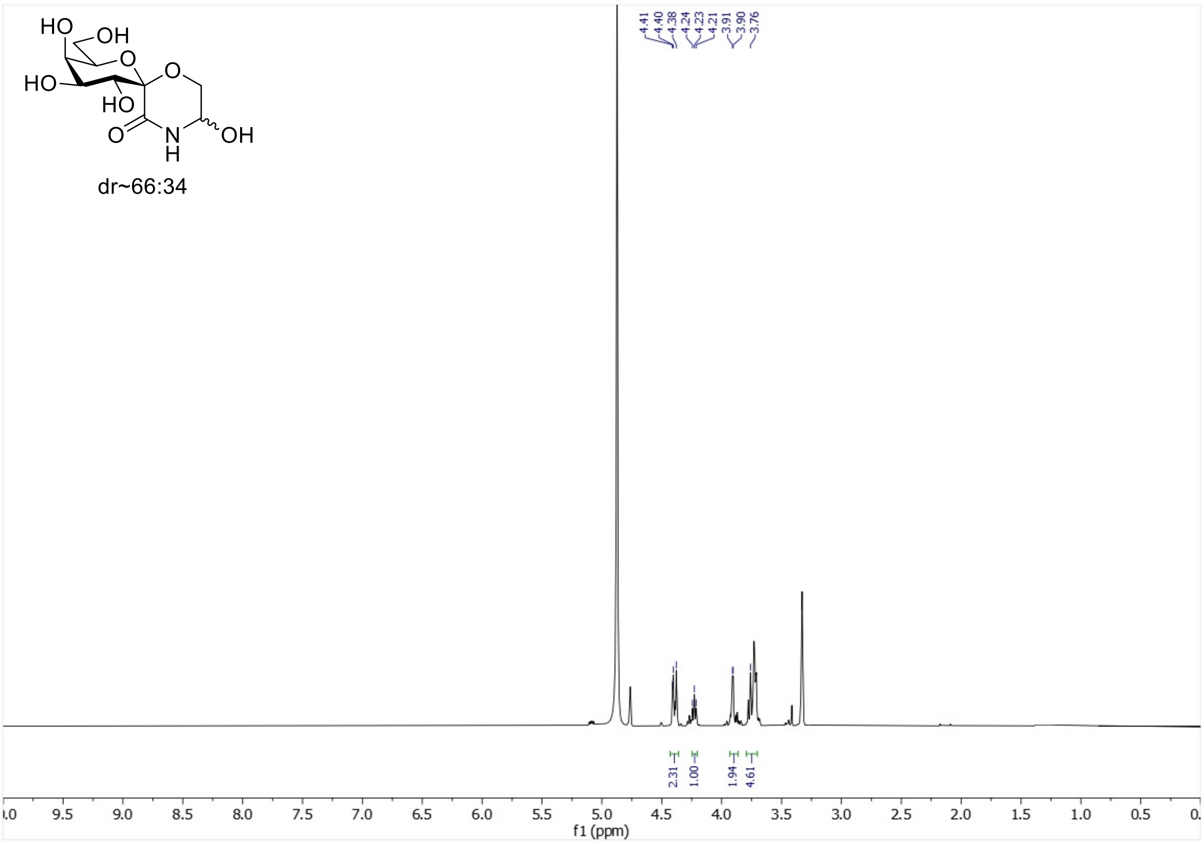

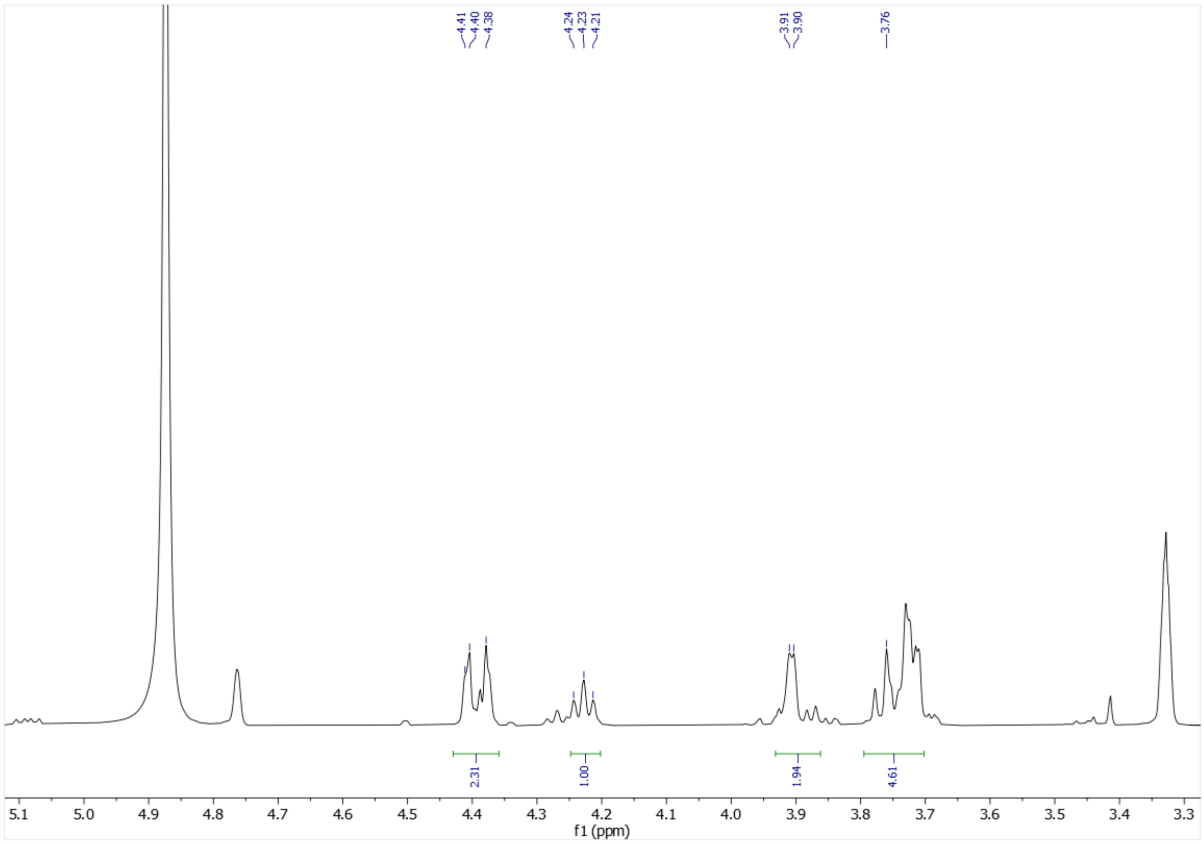

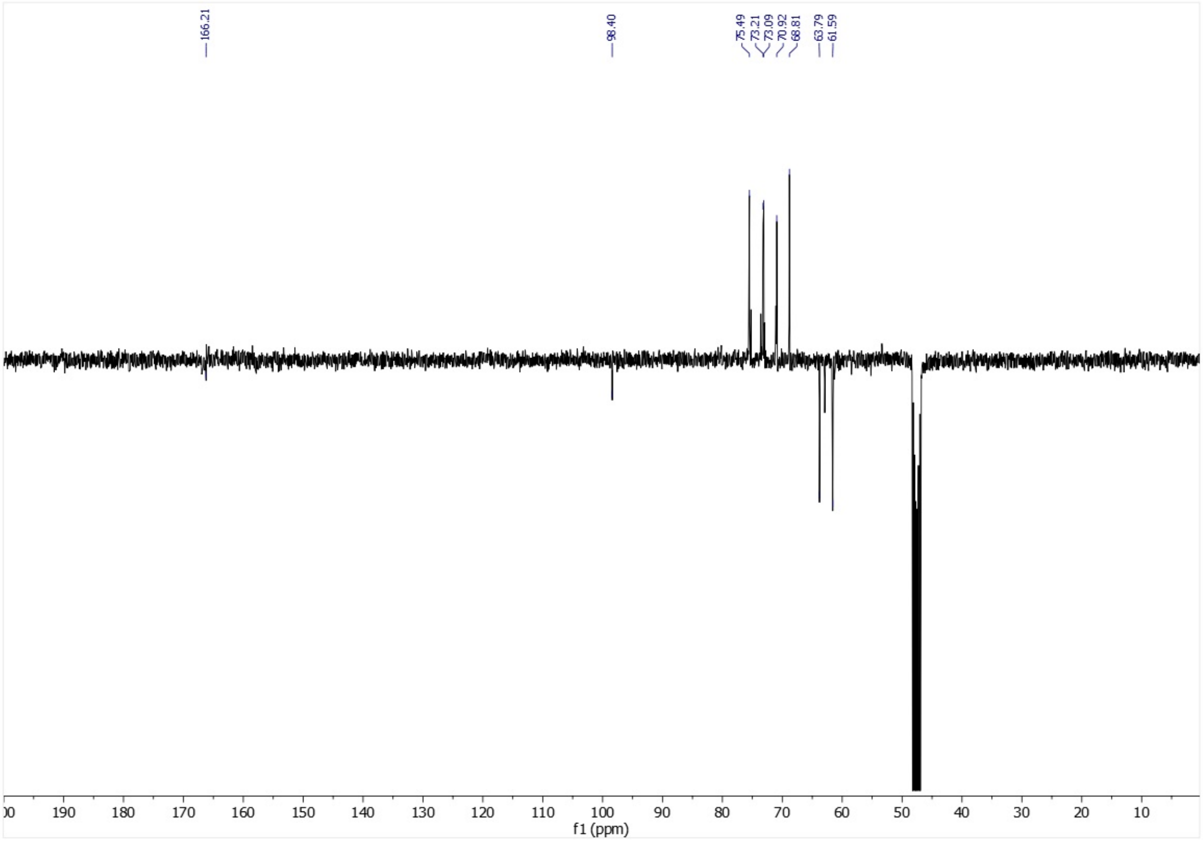
166.21
98.40
75.49
73.21
73.09
70.92
68.81
63.79
61.59
f1 (ppm)

^{1}H (400 MHz) and ^{13}C J-MOD (100 MHz) NMR spectra of compound **38** in CD_3OD

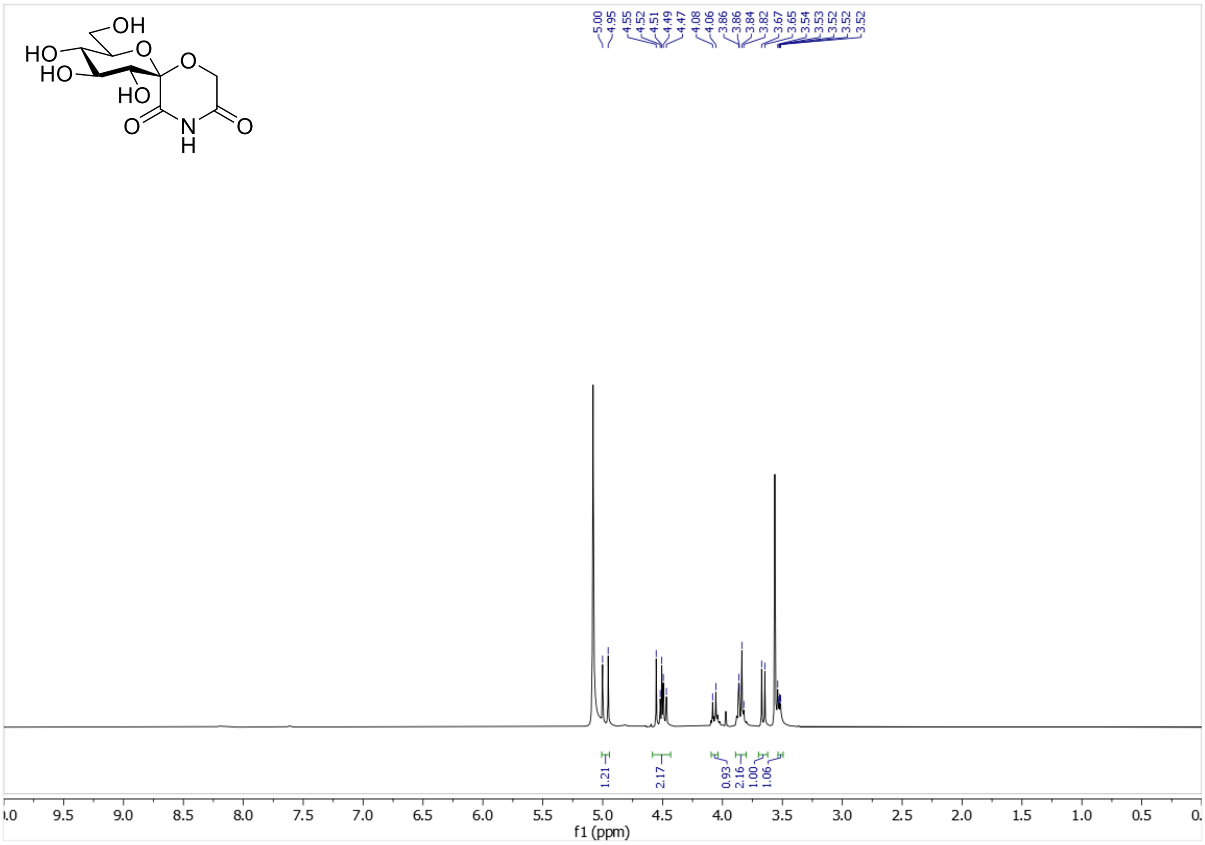

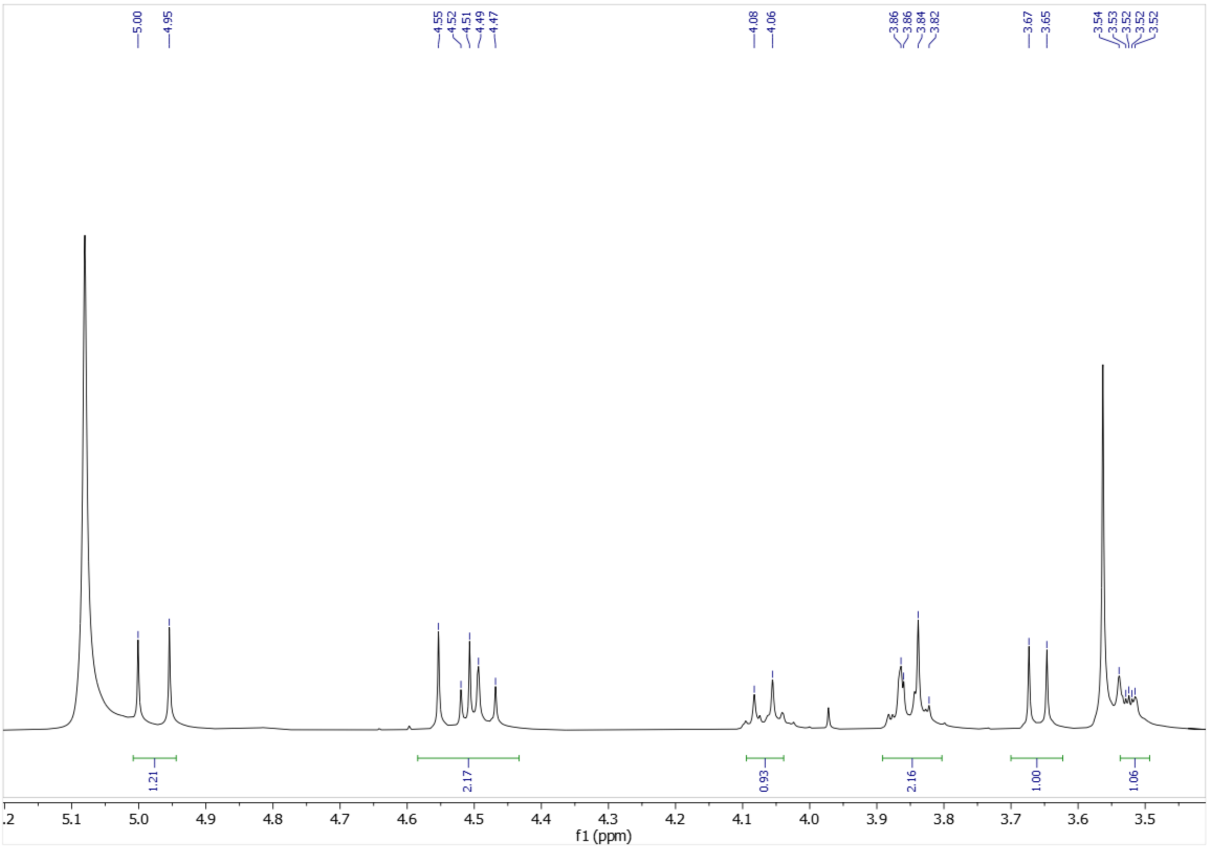

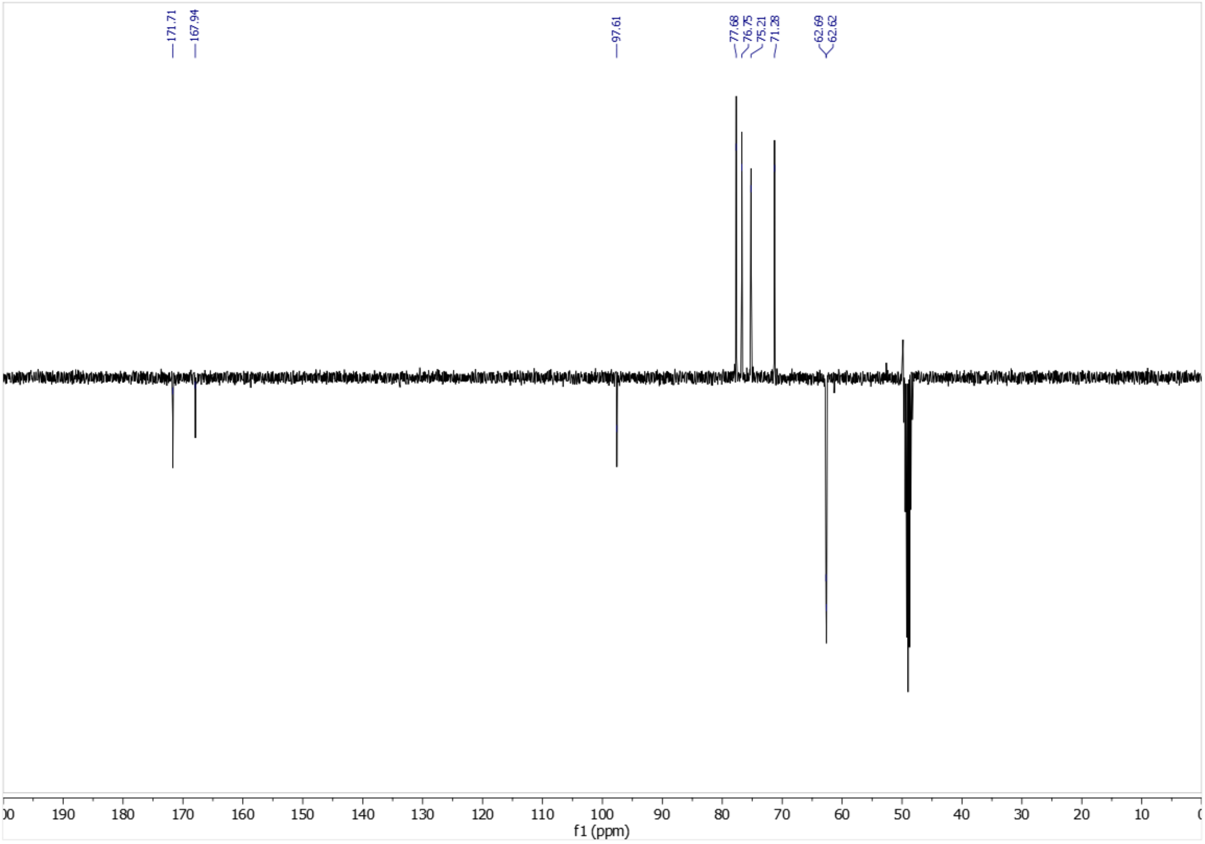

171.71
167.94
97.61
77.68
76.75
75.21
71.28
62.69
62.62
f1 (ppm)

^{1}H (400 MHz) and ^{13}C J-MOD (100 MHz) NMR spectra of compound **39** in CD_3OD

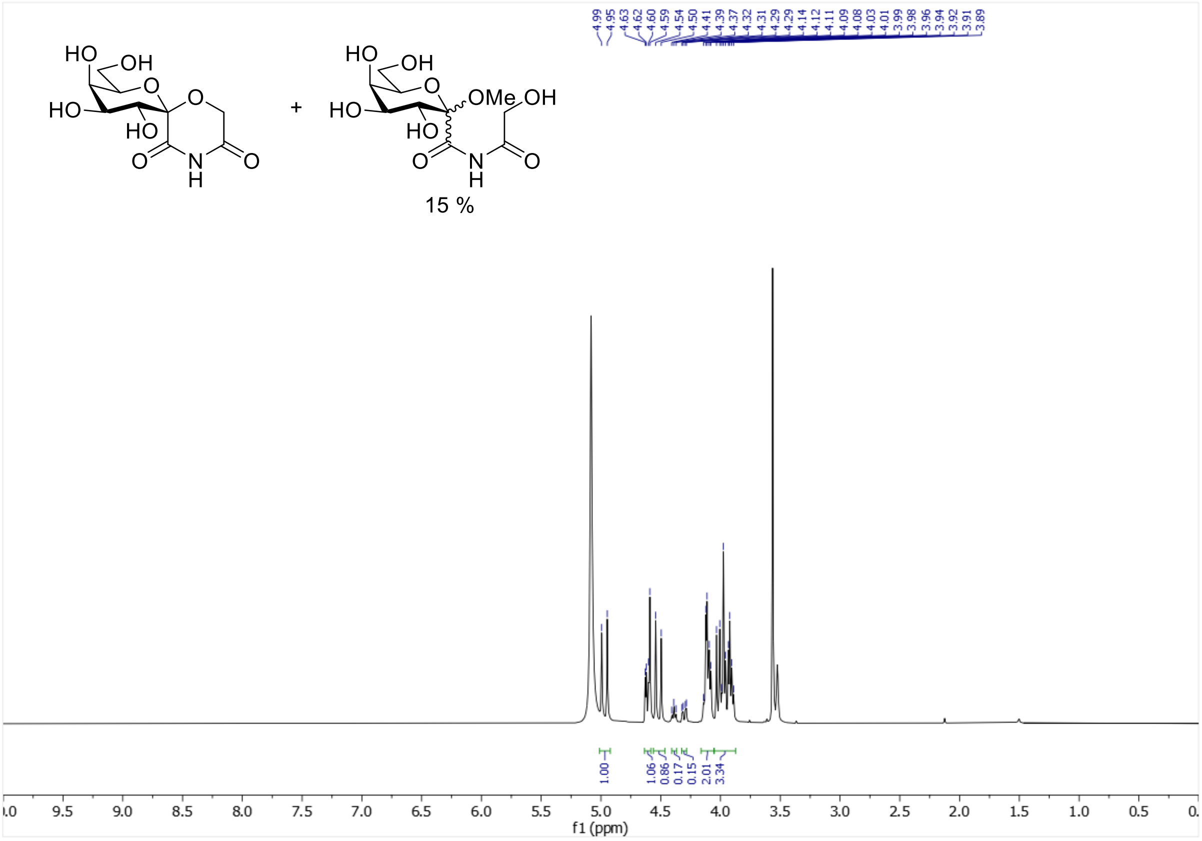

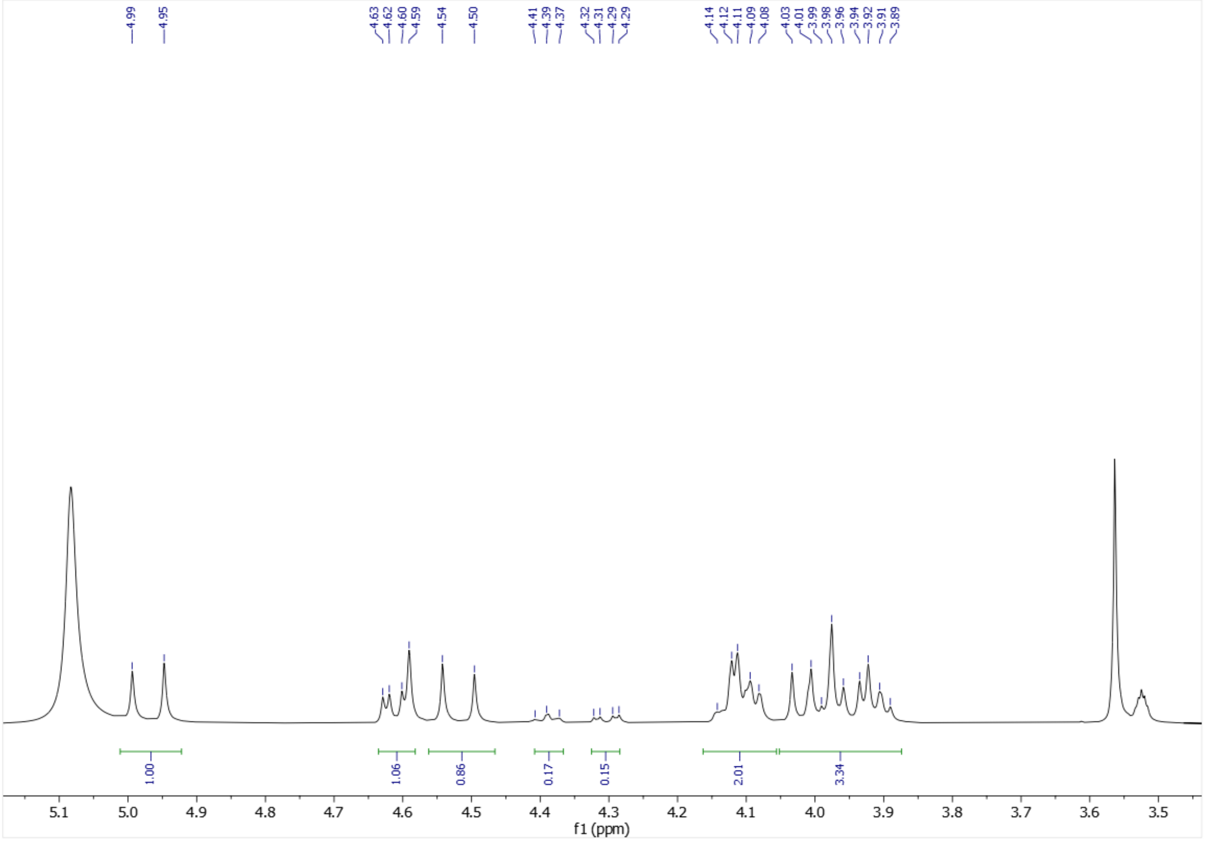

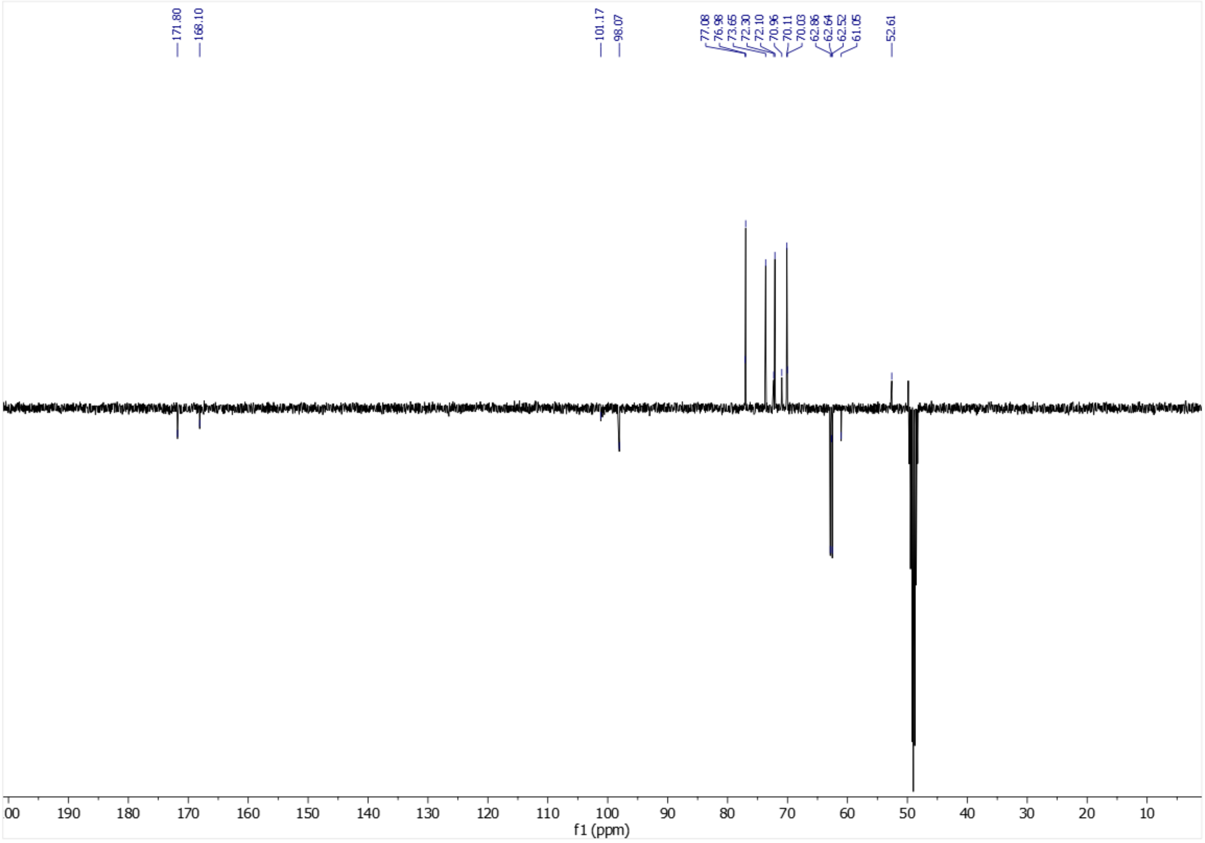

171.80
168.10
101.17
98.07
77.08
76.98
73.65
72.30
72.10
70.96
70.11
70.03
62.86
62.64
62.52
61.05
52.61
00
190
180
170
160
150
140
130
120
110
100
90
80
70
60
50
40
30
20
10
f1 (ppm)

^{1}H (400 MHz) and ^{13}C J-MOD (100 MHz) NMR spectra of compound **40** in CD_3OD

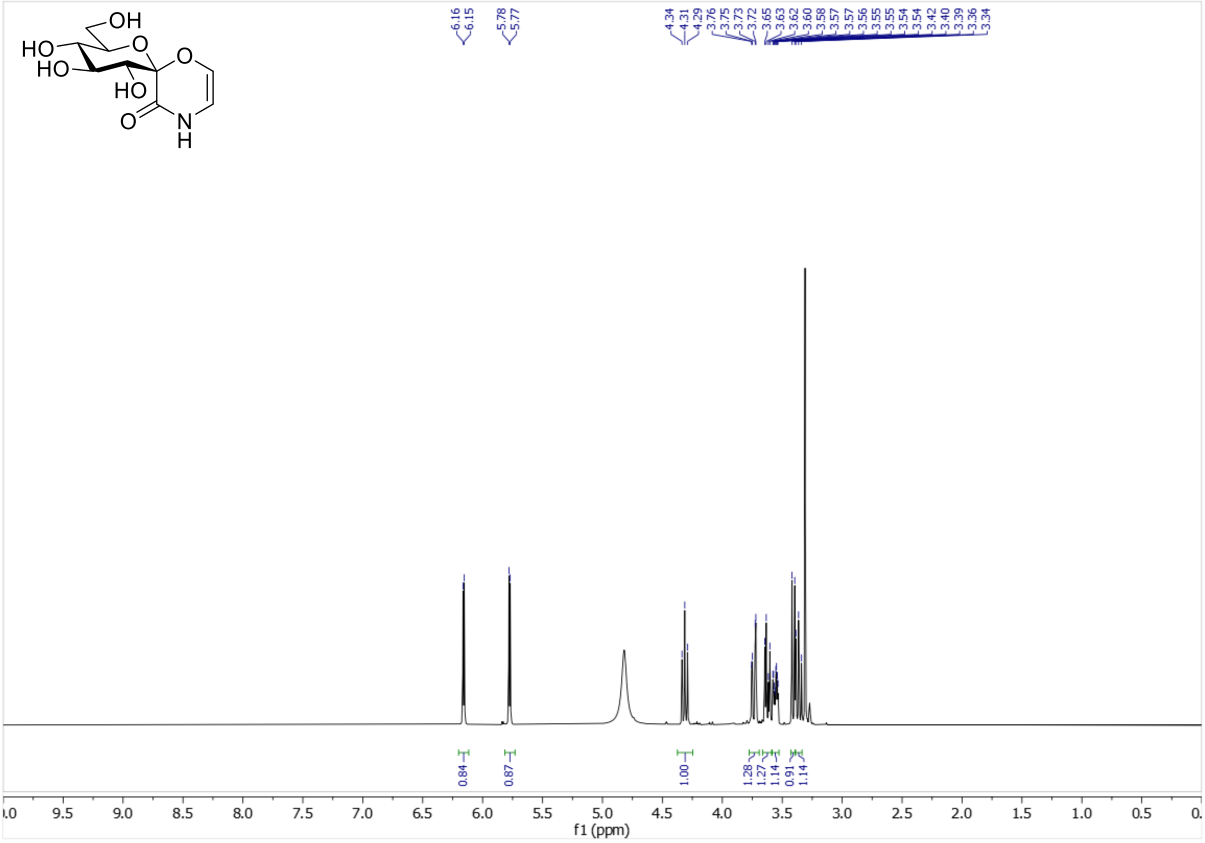

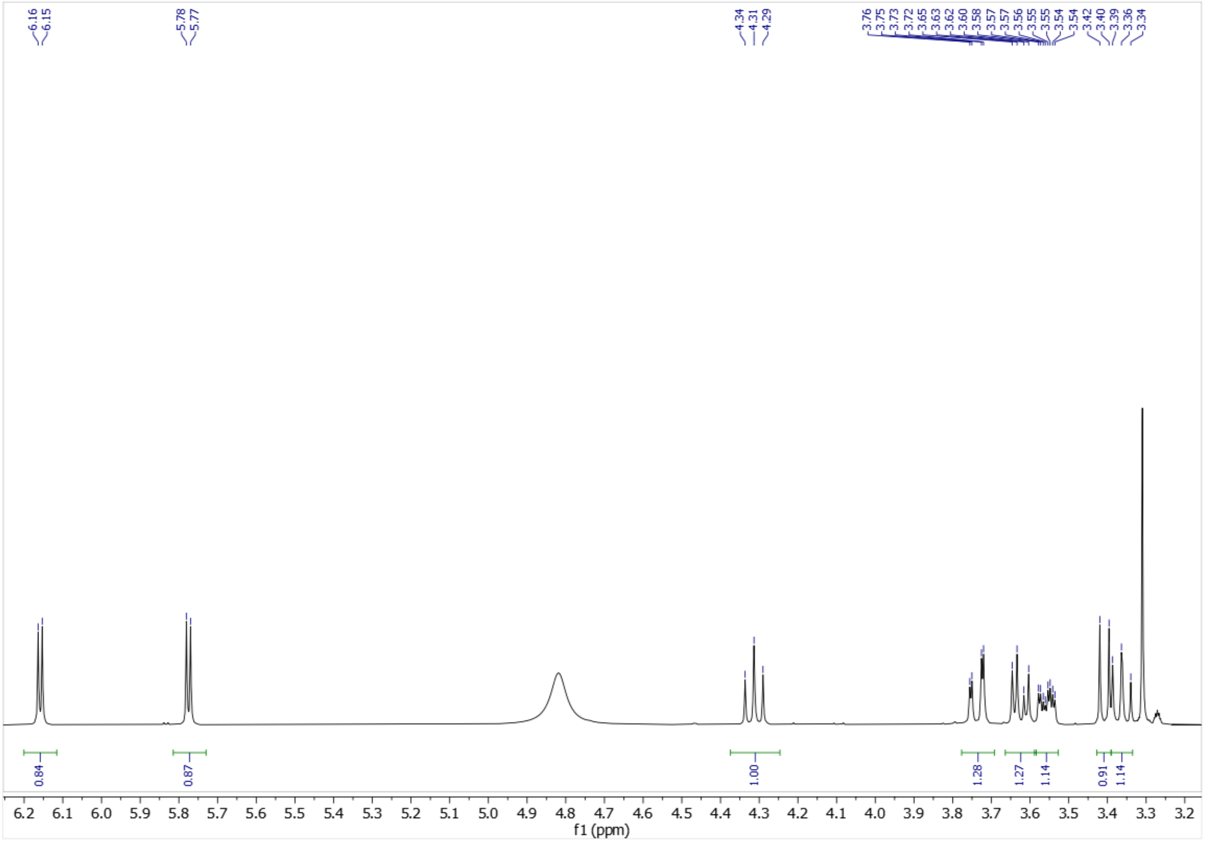

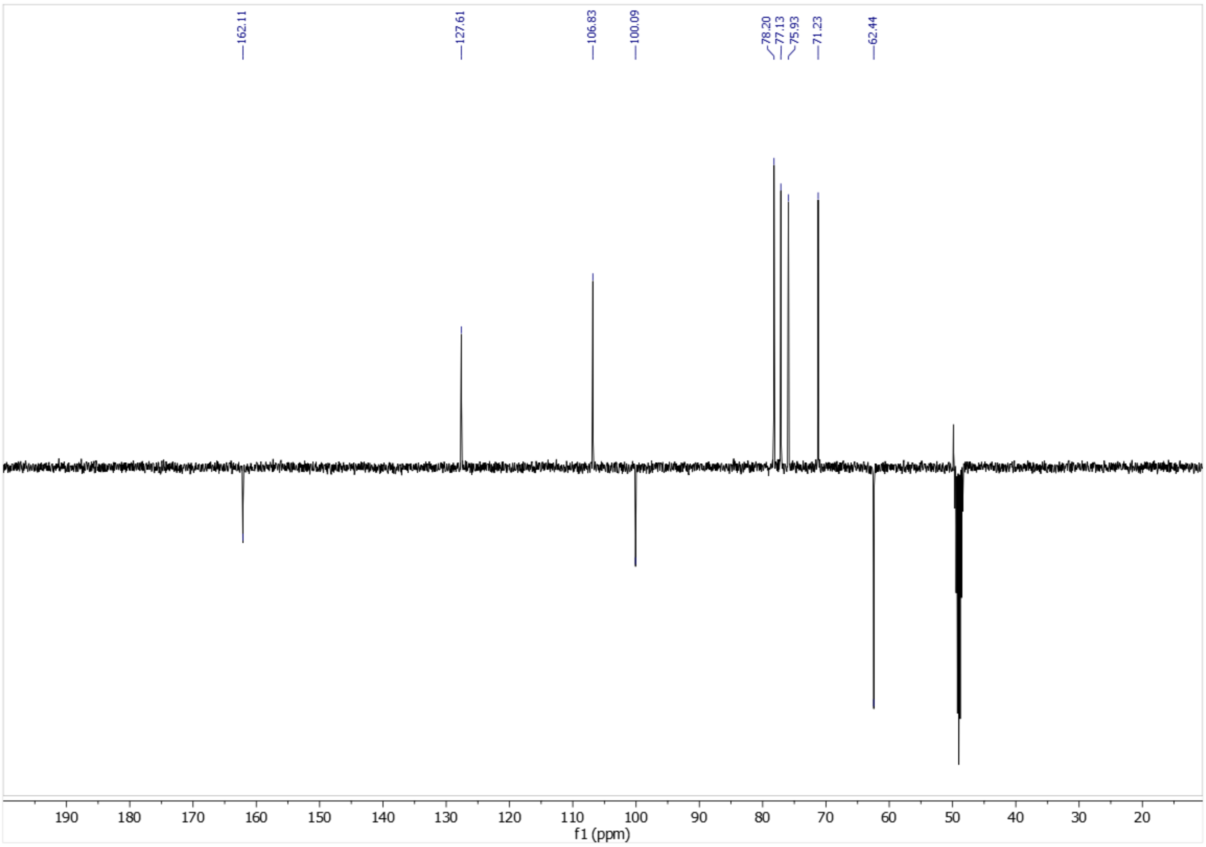

162.11
127.61
106.83
100.09
78.20
77.13
75.93
71.23
62.44
190 180 170 160 150 140 130 120 110 100 90 80 70 60 50 40 30 20
f1 (ppm)

^{1}H (400 MHz) and ^{13}C J-MOD (100 MHz) NMR spectra of compound **41** in CD_3OD

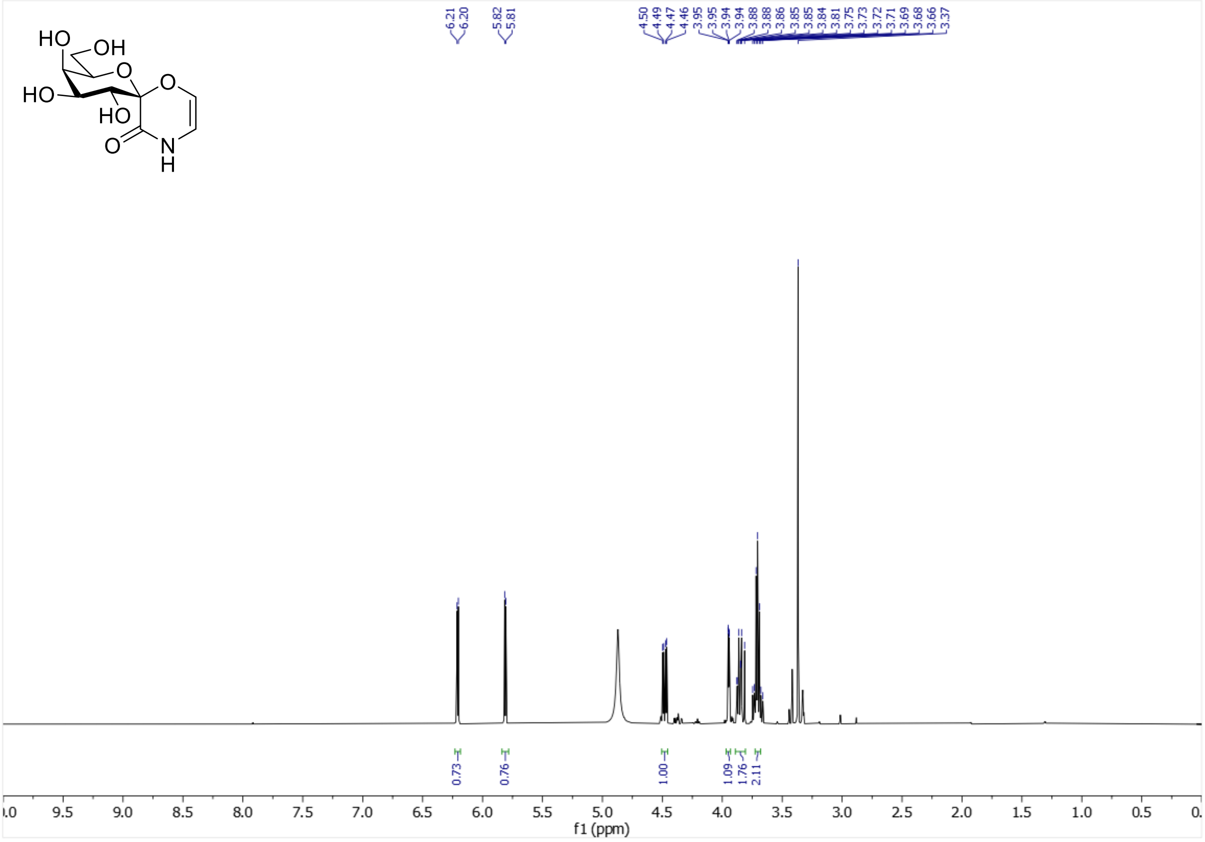

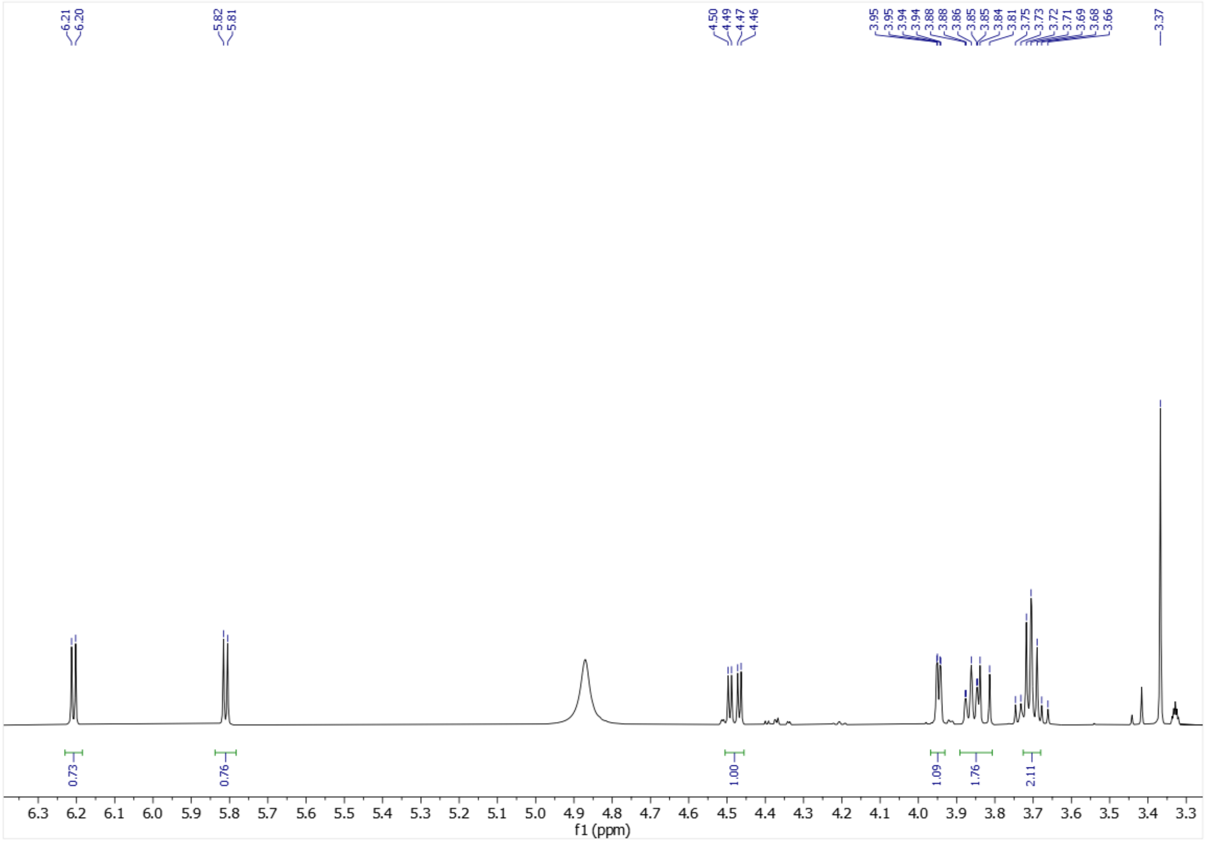

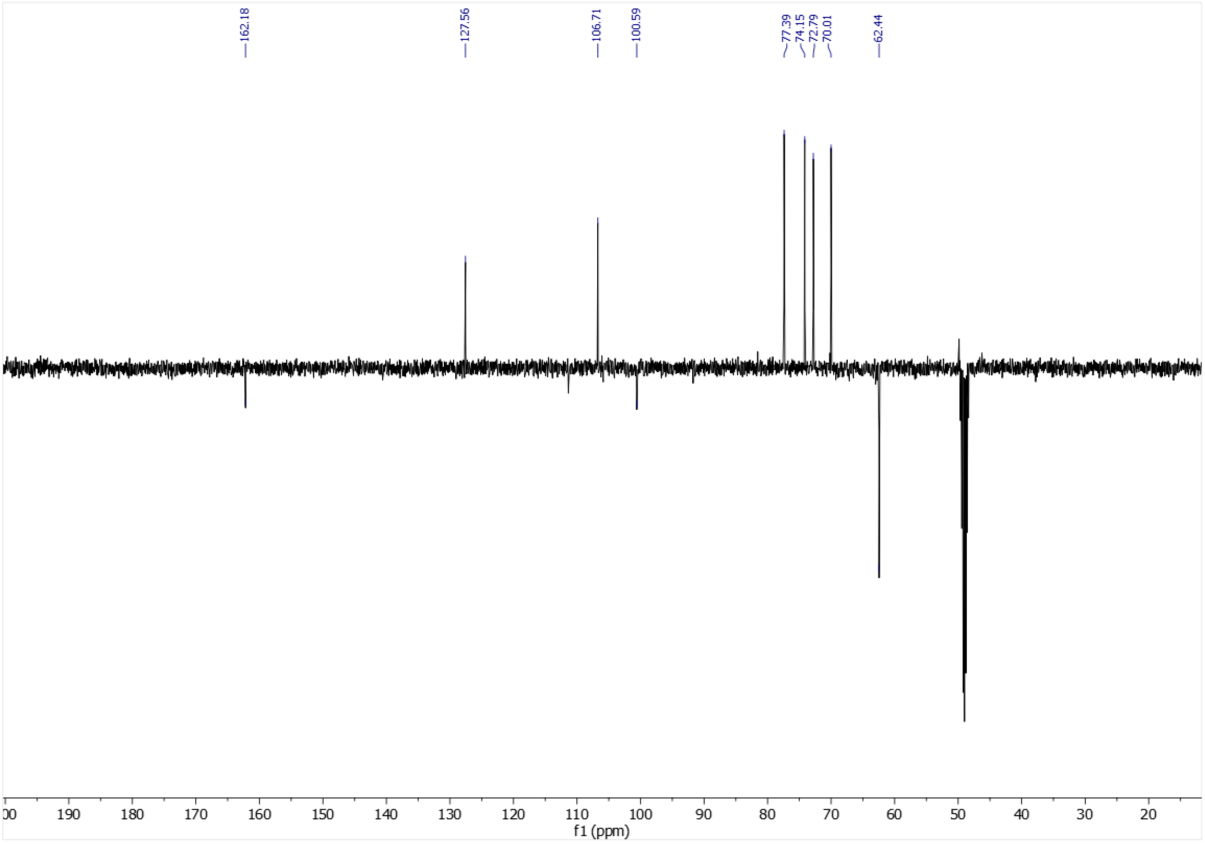

162.18
127.56
106.71
100.59
77.39
74.15
72.79
70.01
62.44
00
190
180
170
160
150
140
130
120
110
100
90
80
70
60
50
40
30
20
f1 (ppm)

^{1}H (400 MHz in CD_3OD) and ^{13}C J-MOD (100 MHz in DMSO-d_6) NMR spectra of compound **42**

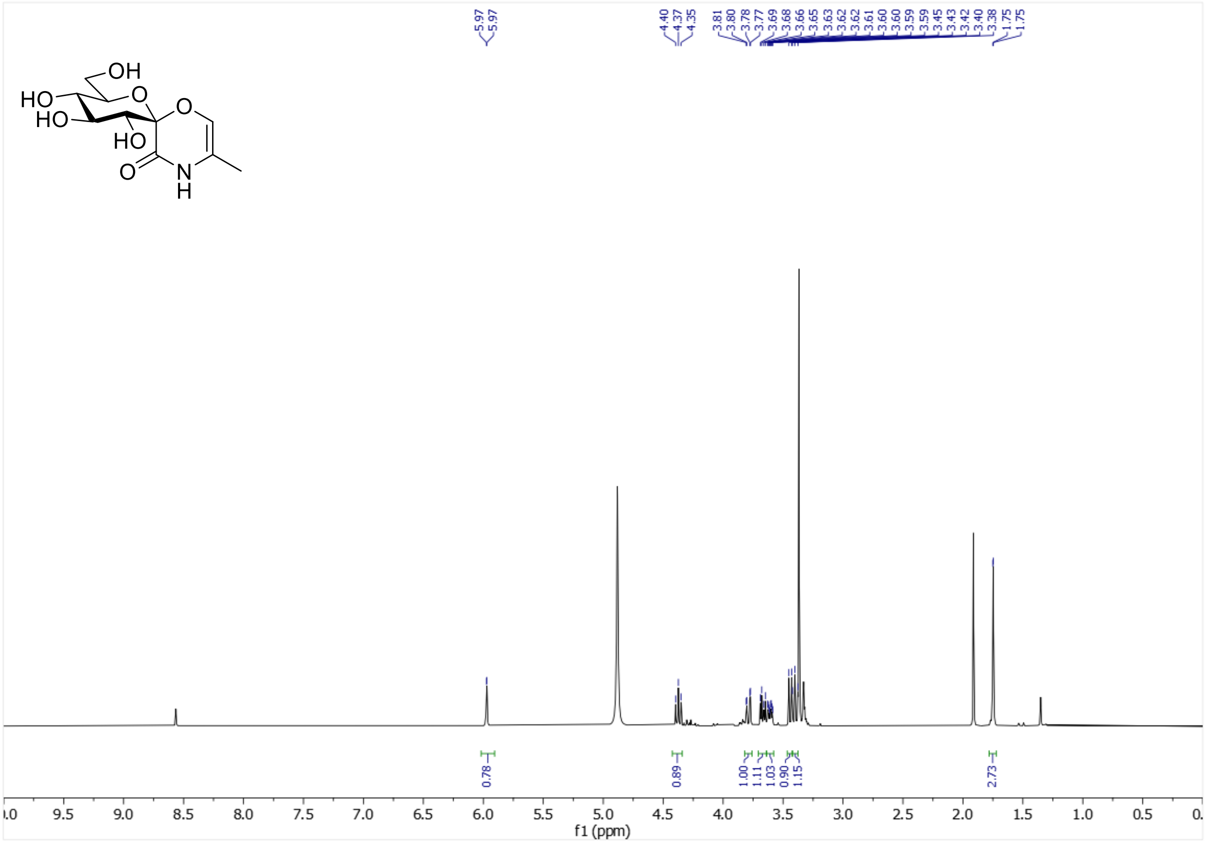

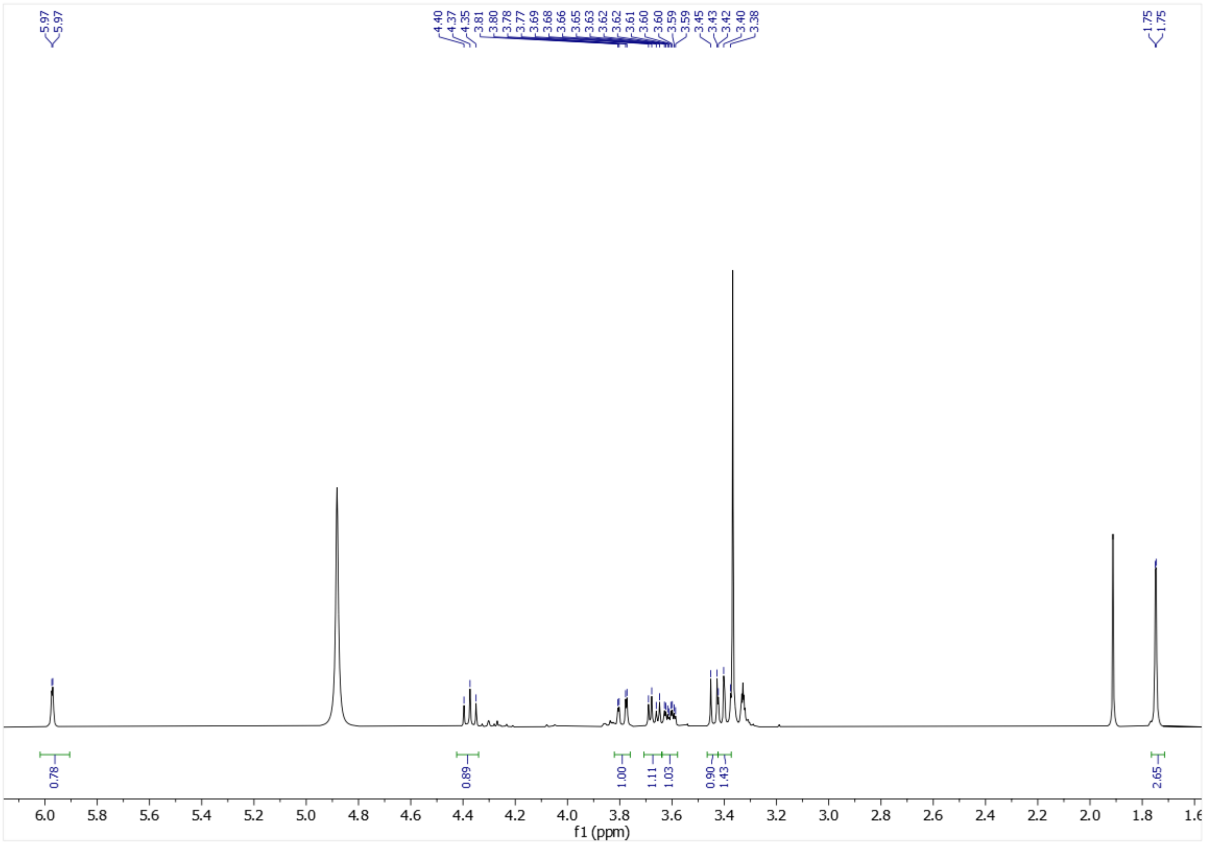

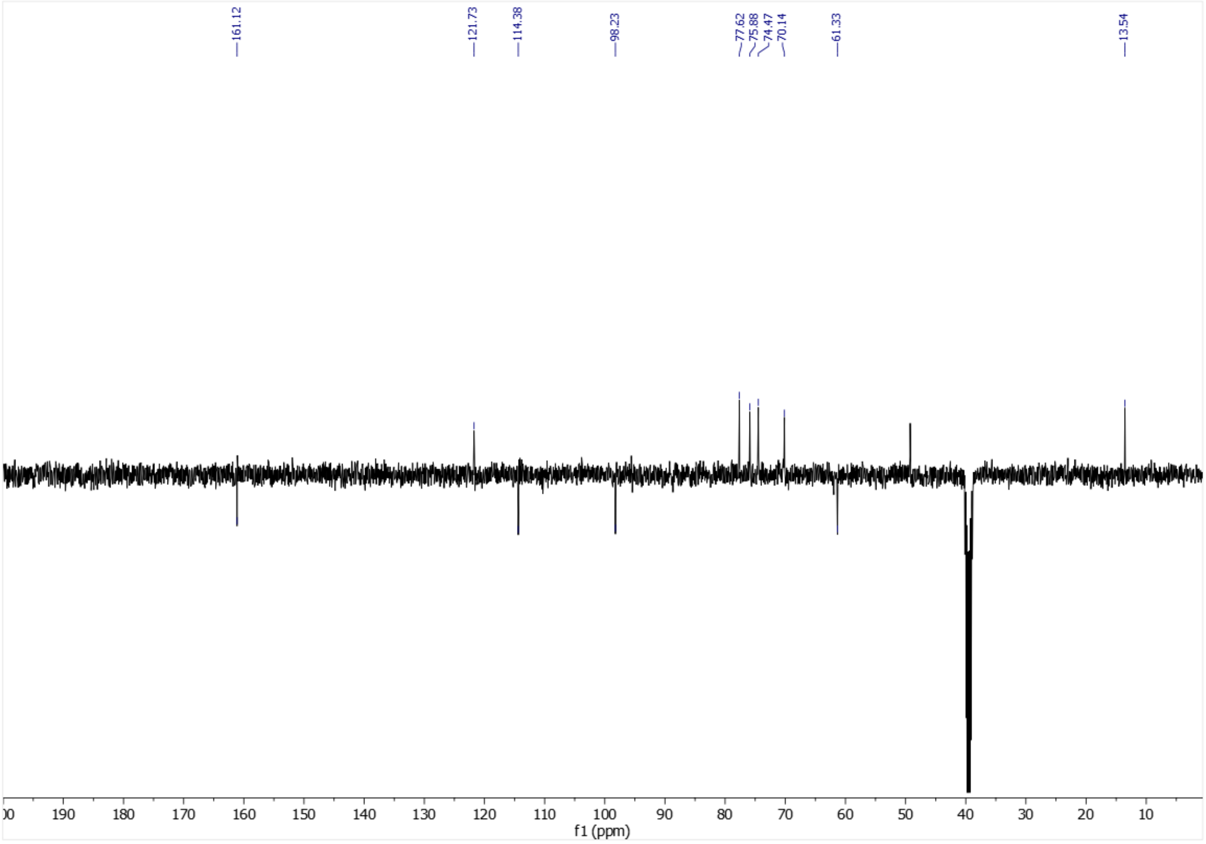

161.12
121.73
114.38
98.23
77.62
75.88
74.47
70.14
61.33
13.54
190 180 170 160 150 140 130 120 110 100 90 80 70 60 50 40 30 20 10
f1 (ppm)
